# The Ugi four-component reaction as a concise modular synthetic tool for photo-induced electron transfer donor-anthraquinone dyads

**DOI:** 10.3762/bjoc.10.100

**Published:** 2014-05-05

**Authors:** Sarah Bay, Gamall Makhloufi, Christoph Janiak, Thomas J J Müller

**Affiliations:** 1 Heinrich-Heine Universität Düsseldorf, Institut für Organische Chemie und Makromolekulare Chemie, Universitätsstraße 1, D-40225 Düsseldorf, Germany; 2 Heinrich-Heine Universität Düsseldorf, Institut für Anorganische Chemie und Strukturchemie, Universitätsstraße 1, D-40225 Düsseldorf, Germany

**Keywords:** absorption spectroscopy, cyclic voltammetry, chromophores, fluorescence, multicomponent reactions, photo-induced electron transfer

## Abstract

Phenothiazinyl and carbazolyl-donor moieties can be covalently coupled to an anthraquinone acceptor unit through an Ugi four-component reaction in a rapid, highly convergent fashion and with moderate to good yields. These novel donor–acceptor dyads are electronically decoupled in the electronic ground state according to UV–vis spectroscopy and cyclic voltammetry. However, in the excited state the inherent donor luminescence is efficiently quenched. Previously performed femtosecond spectroscopic measurements account for a rapid exergonic depopulation of the excited singlet states into a charge-separated state. Calculations of the Gibbs energy of photo-induced electron transfer from readily available UV–vis spectroscopic and cyclovoltammetric data applying the Weller approximation enables a quick evaluation of these novel donor–acceptor dyads. In addition, the X-ray structure of a phenothiazinyl–anthraquinone dyad supports short donor–acceptor distances by an intramolecular π-stacking conformation, an important assumption also implied in the calculations of the Gibbs energies according to the Weller approximation.

## Introduction

**Figure 1:**
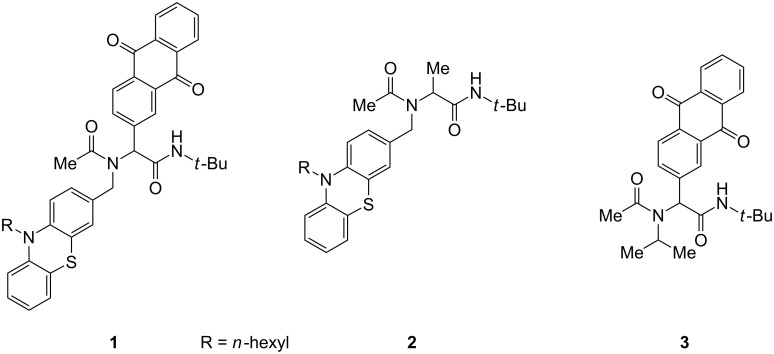
Phenothiazine–anthraquinone dyad **1**, donor-only (**2**) and acceptor-only (**3**) models assembled by Ugi 4CR.

Chromophores, fluorophores, and electrophores, are functional organic materials [1] and constitute active components in molecular electronics [2], photonics [3], and bioanalytics [4–6]. Therefore, the design of well-defined monomolecular structures with electron-donor (Do) and acceptor (Acc) substitution, so called Do–Acc dyads, is a topical field with a paramount academic and technological interest [7–8]. Tailor-made Do–Acc systems represent the fundamental basis for application in molecular electronics and optoelectronics [9–14] and they are employed in organic light-emitting diodes (OLEDs) for a balanced charge transport [15–20] and photovoltaic devices [21–25]. The concept of persistent light-induced charge separation between a donor and an acceptor originates from photosynthesis, nature’s most important process to convert sunlight into chemical energy. One of the most challenging endeavors of mankind is the unlimited generation of electrical energy from sunlight, with great efforts to mimicking photosynthesis by creation of artificial photosynthetic systems [26–27]. The simulation of relevant processes has reached a high level of understanding and the primary process of light-induced charge separation in various types of Do–Acc dyads has been intensively studied [28–29]. This photo-induced electron transfer (PET) [30–34] has been investigated with donors such as porphyrines, polycyclic aromatic hydrocarbons, perylenediimides and (oligo)thiophenes [35–36], tetrathiafulvalenes [37], as well as phenothiazine and its derivatives [22,38–40]. The latter have become attractive electrophores due to their reversible and tunable oxidation potential. Interestingly quenching of the phenothiazine inherent fluorescence offers a facile evidence for the occurrence of intramolecular PET in phenothiazine-containing Do–Acc dyads [41–42]. As suitable acceptor moieties C_60_ fullerene [43–45], and quinones, such as 9,10-anthraquinone as a potential two electron acceptor, have been commonly used in Do–Acc arrangements [46–51]. In previous studies phenothiazine–anthraquinone couples have been introduced into peptide scaffolds [52–54] and rigid Do–Acc dyads [55]. Nevertheless, a modular and rapid access by multicomponent reactions to these types of functional targets has never been explored prior to our recent studies [56]. For instance, the Ugi four-component reaction (Ugi 4CR) [57–60] establishes the chemically robust α-aminoacylamide backbone in one step and with high diversity. Therefore, it is also extensively used in medicinal and combinatorial chemistry for lead finding and optimization [61]. We have recently employed the Ugi 4CR as a one-step process to simultaneously introduce a phenothiazine-functionalized amine and an anthraquinone-substituted aldehyde together with acetic acid and *tert*-butyl isocyanide for rapidly assembling a donor–acceptor conjugate **1** displaying a photo-induced electron transfer leading to a charge-separated state with a lifetime of >2 ns (Figure 1), as elucidated by femtosecond transient absorption spectroscopy [56]. The donor-only (**2**) and acceptor-only (**3**) models for spectroscopic comparison were obtained analogously.

For enabling rapid accesses to functional π-systems, such as Do–Acc dyads for photo-induced charge separation, besides a robust, flexible diversity-oriented one-pot reaction, such as the Ugi 4CR, a quick semiquantitative estimation of the feasibility of the charge-separated state based upon inexpensive analytical methods is highly desirable. Here we present the synthetic versatility of this multicomponent approach to Do–anthraquinone dyads, as exemplified by phenothiazinyl and carbazole moieties as donors, and comprehensive physical organic studies of electronic and electrochemical properties investigated by steady state UV–vis and fluorescence spectroscopy as well as cyclic voltammetry. The obtained data are interpreted in the light of the Weller approximation to estimate the probability for charge separation by photo-induced electron transfer based upon its Gibbs energy calculated from the analytical data and donor–acceptor distances of lowest energy conformers from inexpensive force field computations.

## Results and Discussion

### Synthesis and structure

**Scheme 1:**
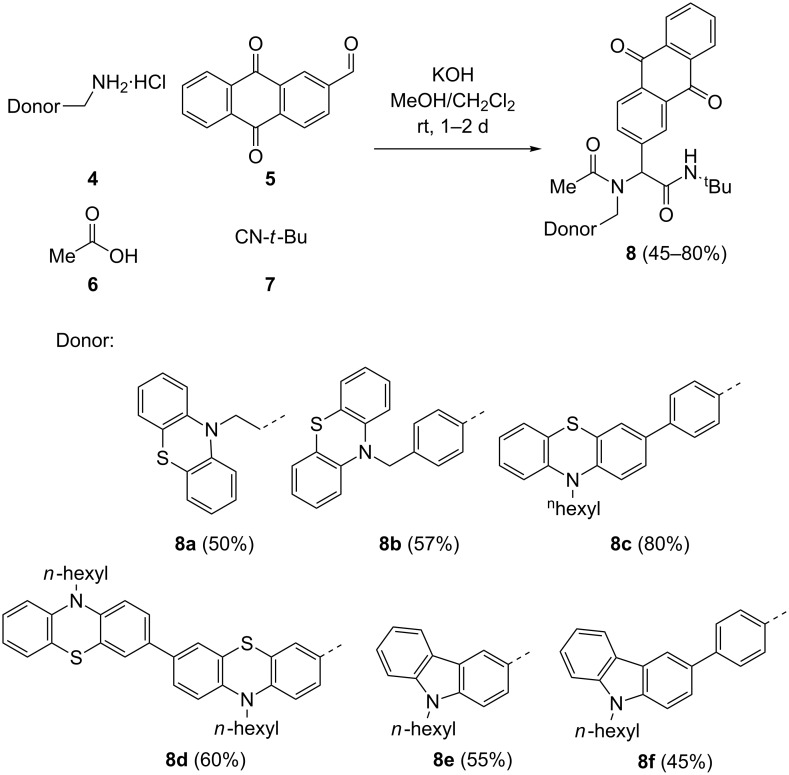
Ugi 4CR synthesis of donor–anthraquinone dyads **8**.

Within the concept of diversity-oriented syntheses of chromophores [62–69] we have established accesses to chromophores in a one-pot fashion based upon transition metal catalysis as an entry to consecutive multicomponent [70–71] and domino reactions [72]. The highly convergent synthetic approach by multicomponent reactions should as well be applicable to functional Do–Acc dyads. Therefore, we set out to place electron-rich phenothiazinyl and carbazolyl derivatives **4** as amino component in the Ugi 4CR, whereas the electron acceptor was introduced as anthraquinone-2-carbaldehyde (**5**). Acetic acid (**6**) and *tert*-butyl isocyanide (**7**) were the two residual components (Scheme 1) [56].

**Scheme 2:**
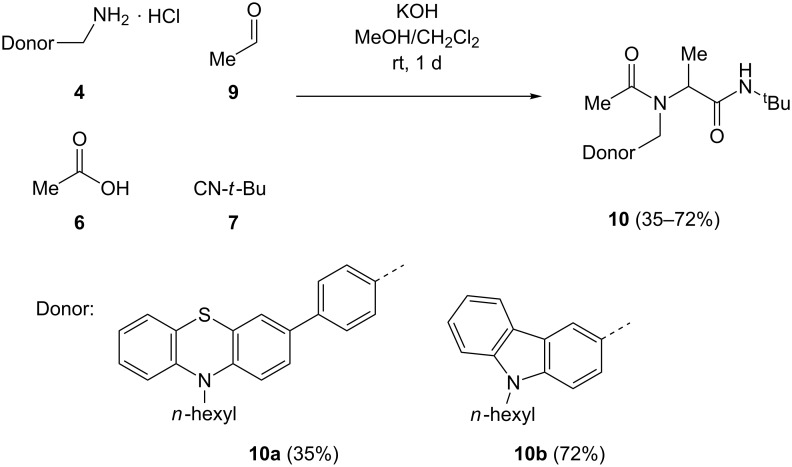
Ugi 4CR synthesis of donor-only reference systems **10**.

The most favorable solvent for Ugi 4CR is methanol. However, to increase solubility portions of dichloromethane were added to assure a homogeneous solution. For liberating the free base from methylamine hydrochlorides **4**, potassium hydroxide was employed as a base. The methylamine hydrochlorides **4**, in turn, were readily available from the corresponding cyano compounds [73] by lithium alanate reduction in diethyl ether [74]. The corresponding donor–anthraquinone dyads **8** were isolated in moderate to good yields. For reference, donor-only systems **10** were also prepared by Ugi 4CR from the amino derivatives **4** and acetaldehyde (**9**), in moderate to good yield, with acetic acid (**6**) and *tert*-butyl isocyanide (**7**) as the corresponding acid and isonitrile components (Scheme 2).

**Figure 2:**
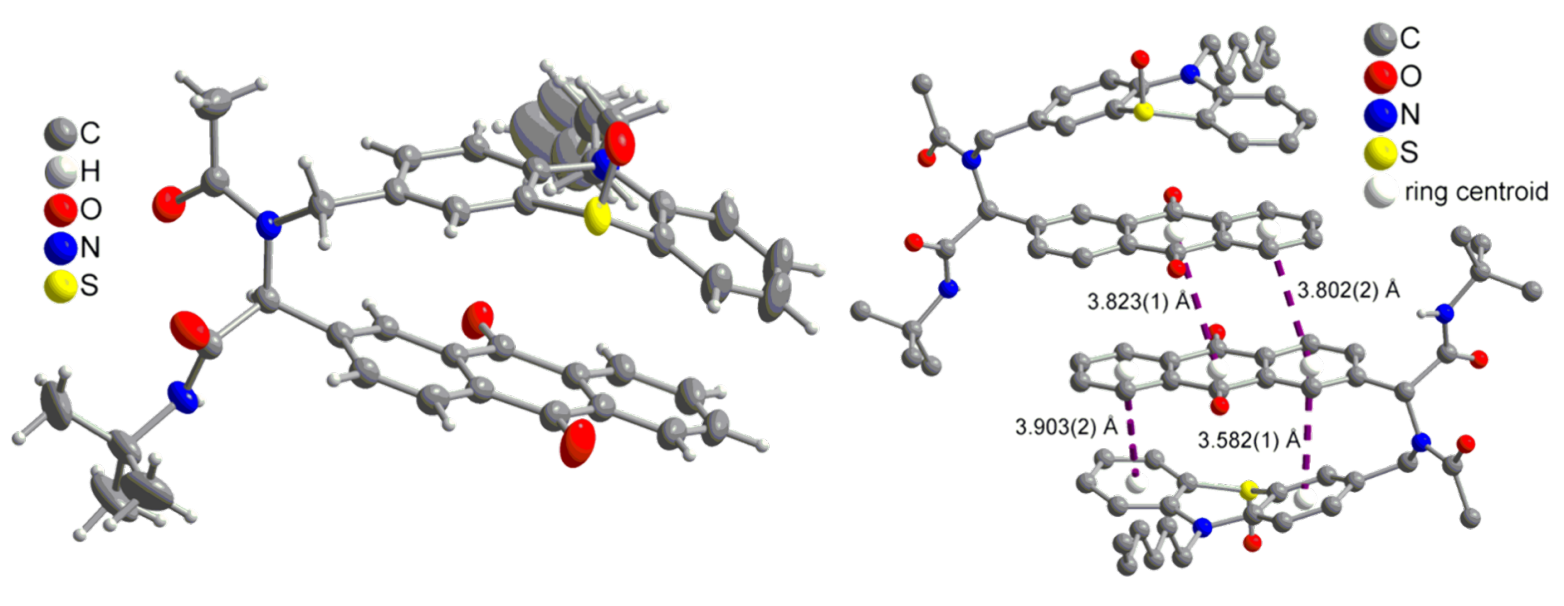
Molecular structure of S(O)-**1** (left) (30% ellipsoids, except for the CH_3_CH_2_ end of the hexyl group, the disordered water molecules were omitted for clarity) and the intra- and intermolecular π–π-stacking interactions between an inversion symmetry related pair (right, hydrogen atoms were omitted for clarity).

The appearance of single signal sets in the ^1^H and ^13^C NMR spectra of **8** and **10** unambiguously supports the structural assignment and that isomeric mixtures due to restricted amide-bond rotation can be excluded. Besides mass spectrometry and combustion analysis the structure of phenothiazine–anthraquinone dyads **8a–d** was additionally supported by an X-ray structure analysis of the partially oxidized derivative of compound S(O)-**1** (Figure 2) [75]. The phenothiazine and anthraquinone moieties are aligned by intramolecular π-stacking with an average distance of ~3.9 Å [76–77]. In the unit cell the *R*- and *S*-enantiomers of a single diastereomer (S-oxide) are arranged in pairs resulting in four stacked (hetero)aromatic units (Figure 2), so that the anthraquinone moieties of two molecules display an average distance of ~3.8 Å. Based upon the X-ray data quantum mechanical computations on this conformer were envisioned (vide infra).

### Electronic properties and electronic structure

**Figure 3:**
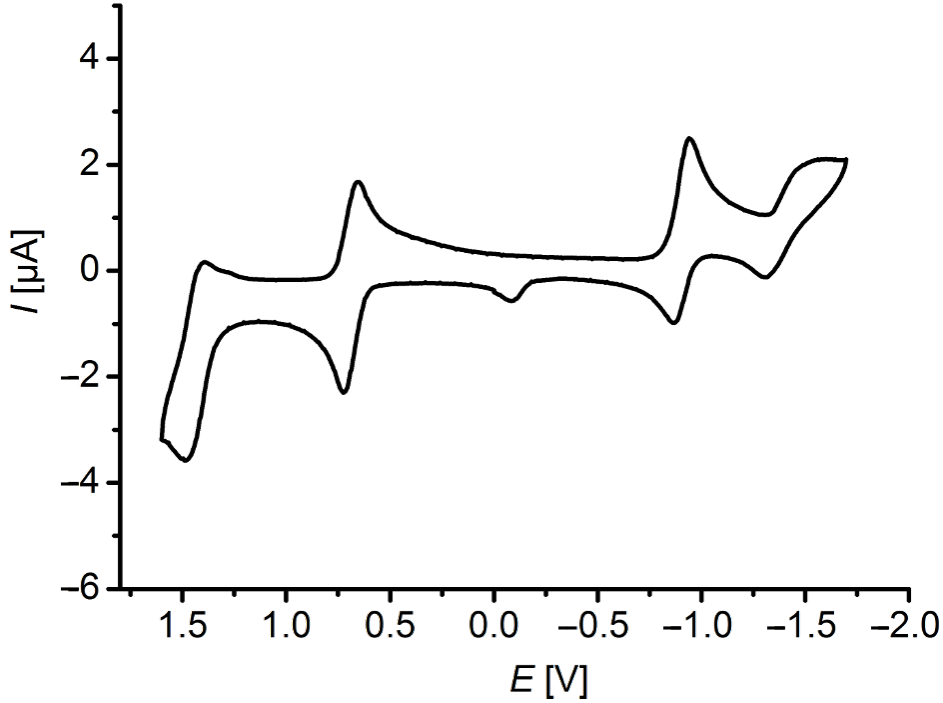
Cyclic voltammogram of dyad **8c** (recorded in CH_2_Cl_2_, *T* = 298 K, *c* (**8c**) = 0.1 mol·L^−1^, Pt working electrode, Pt counter electrode, Ag/AgCl reference electrode, electrolyte N(*n*-Bu)_4_PF_6_, scan rate of 250 mV·s^−1^).

All dyads **8** and **1** as well as the reference systems **2**, **3**, and **10** were studied by cyclic voltammetry in dichloromethane at room temperature (Table 1, Figure 3).

**Table 1:** Cyclovoltammetric data of the dyads **8** and the reference systems **1**, **2**, **3** and **10** (recorded in CH_2_Cl_2_, *T* = 298 K, *c* = 0.1 mol·L**^−^**^1^, Pt working electrode, Pt counter electrode, Ag/AgCl reference electrode, electrolyte N(*n-*Bu)_4_PF_6_, scan rates *v* of 100, 250, 500 and 1000 mV·s**^−^**^1^)^a,b^.

Compound	*E*_1/2_^0/+1^ [mV]	*E*_1/2_^+1/+2^ [mV]	*E*_1/2_**^−^**^1/0^ [mV]	*E*_1/2_**^−^**^2/^**^−^**^1^ [mV]

**8a**	780	–	−850^c^	−1500^c^
**8b**	780	–	−870^c^	−1370^c^
**8c**	710	1450	−900^c^	−1390^c^
**8d**	630	800	−880^c^	−1380^c^
**8e**	1220^c^	–	−940^c^	−1420^c^
**8f**	1170^c^	–	−920^c^	–
**1**	710	–	−870^c^	−1430^c^
**3**	–	–	−920^c^	−1430^c^
**2**	730	–	–	–
**10a**	710	1430	–	–
**10b**	1320^c^	–	–	–

^a^Calibrated against ferrocene as an internal standard (*E*_0_^0/+1^ = 450 mV). ^b^The half-wave potentials *E*_1/2_ were extrapolated to a scan rate of *v* = 0 mV·s**^−^**^1^ from the linear correlation plot of the differences of the anodic and cathodic peak potentials against 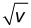. ^c^Quasi-reversible redox wave.

The cyclic voltammograms were recorded at scan rates *v* of 100, 250, 500 and 1000 mV·s^−1^ and the differences of anodic and cathodic peak potentials were plotted against 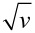 for extrapolating the half-wave potentials *E*_1/2_ for a scan rate *v* = 0 mV·s^−1^ assuming an ideal Nernstian behavior. In the cyclic voltammograms of the phenothiazine–anthraquinone dyads **8a**–**d**, typical for phenothiazine derivatives [41–42,78], first reversible oxidations *E*_1/2_^0/+1^ between 630 and 780 mV are found, and in addition the cyclic voltammograms of **8c** and **8d** display second oxidation waves *E*_1/2_^+1/+2^ at 1450 (**8c**) and 800 mV (**8d**). The direct comparison of the 3-phenyl derivative **8c** with the donor-only reference **10a** clearly indicates that the proximity of the electron-withdrawing anthraquinone moiety does not affect the first and second reversible oxidations of the phenothiazinyl moiety in the Do–Acc dyad **8c**. The system **8d** containing two conjugated phenothiazinyl moieties is particular since the second oxidation wave originates from the electronic coupling within the diphenothiazine unit [79–80]. These values for first and second oxidation can also be found in the donor-only reference compound **10a**. Furthermore two quasi-reversible reductions stemming from the anthraquinone core can be detected for *E*_1/2_^−1/0^ between −850 to −900 mV and for *E*_1/2_^−2/−1^ between −1370 and −1500 mV, in good agreement with literature data [81–82]. Within a margin of 70 mV both reduction waves fall into the same region as for the anthraquinone-only reference **3** with *E*_1/2_^−1/0^ at −920 mV and *E*_1/2_^−2/−1^ at −1430 mV. Cum grano salis the electrochemical behavior of the novel dyads **8a**–**d** are very similar to the parent system **1**. The carbazole-based dyads **8e** and **8f** only show quasi-reversible oxidation waves at an estimated *E*_1/2_^0/+1^ of 1220 and 1170 mV, yet in good agreement with the behavior of the carbazole-only reference **10b** with an estimated *E*_1/2_^0/+1^ at 1320 mV. The carbazole–anthraquinone dyads **8e** and **8f** display the anthraquinone-centered first quasi-reversible reduction wave *E*_1/2_^−1/0^ at −940 (**8e**) and −920 mV (**8f**), the second quasi-reversible reduction wave is only found for dyad **8e** and appears at *E*_1/2_^−2/−1^ = −1420 mV. The comparison of the cyclic voltammograms between all dyads **8** and the reference systems **2**, **3**, and **10** clearly shows that the donor and anthraquinone moieties are essentially electronically decoupled in the electronic ground state. Therefore, in the electronic ground state the electronic effects should behave additively, i.e., as if the donors and anthraquinones were placed at large distances.

**Figure 4:**
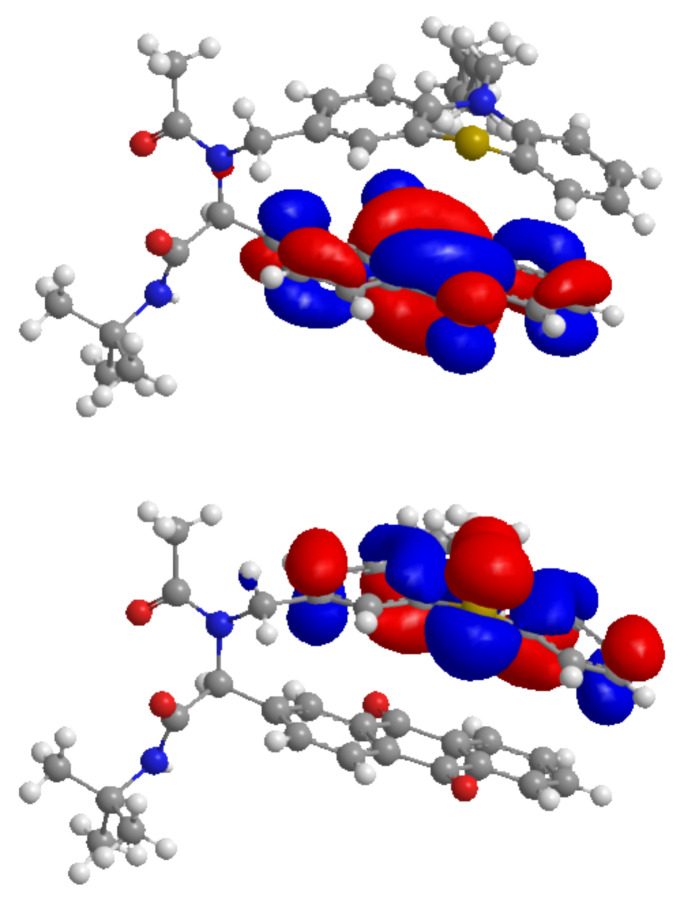
DFT-computed (B3LYP, 6-311G*) frontier molecular orbitals HOMO (bottom) and LUMO (top) of the phenothiazine–anthraquinone dyad **1**.

Based on the starting geometry from the X-ray structure analysis of S(O)-**1** the frontier molecular orbitals (FMO) of **1** were calculated on the DFT level of theory with the B3LYP functional and the Pople basis set 6-311G* (Figure 4) [83–86]. It is noticeable that the coefficient density of the HOMO is almost completely localized on the phenothiazine unit whereas the coefficient density of the LUMO resides on the anthraquinone core, supporting the electronic decoupling of the donor and the acceptor in the electronic ground state. In conclusion the computation underlines that in dyad **1** an electronic prerequisite for electronically favored electron-transfer processes in donor–acceptor systems is the spatial proximity of PT and AQ. This conformer is additionally stabilized by π-stacking of the donor and the acceptor, which is adopted in the solid state and results in a strong bathochromically shifted absorption of the solid.

**Figure 5:**
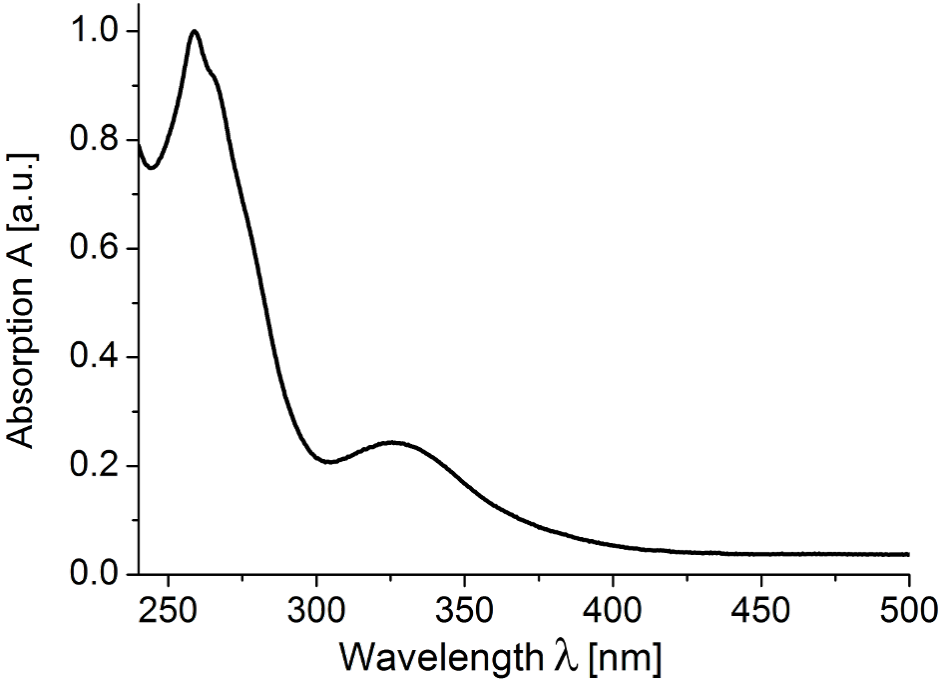
Normalized absorption spectra of the phenothiazine–anthraquinone dyad **8c** (recorded in CH_2_Cl_2_, *c* (**8c**) = 2.5∙10^−5^ mol·L^−1^, *T* = 298 K).

Furthermore, the electronic properties of the donor-anthraquinone dyads **8** were studied by absorption and emission spectroscopy (Table 2). All phenothiazine-based dyads **8a–d** show very similar absorption characteristics with a major absorption band around 259 nm and a lower intensity band around 325 nm (Figure 5). According to the phenothiazine-only (**10a**) and anthraquinone-only (**3**) references phenothiazine as well as anthraquinone absorb in the same region. In the spectrum of the carbazole dyad **8e** the carbazole-typical absorption maxima can be found (cf reference systems **3** and **10b**), whereas the spectrum of **8f** displays just two absorption bands at 255 and 285 nm, originating from the 3-phenylcarbazole moiety.

**Table 2:** Absorption and emission characteristics of the dyads **8**, **1**, and the reference systems **2**, **3**, and **10** (recorded in CH_2_Cl_2_, *c* = 1.4–3.1∙10^−5^ mol·L^−1^, *T* = 298 K).

Compound	Absorption	Emission	Stokes shift
	λ_max,abs_ [nm] (ε [L·mol·cm^−1^])	λ_max,em_ [nm]	Δ 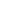 [cm^−1^]^a^

**8a**	257 (44000), 319 (5000)	–^b^	–
**8b**	258 (87000), 325 (10000)	–^b^	–
**8c**	258 (92000), 326 (19000)	–^b^	–
**8d**	259 (145000), 326 (40000)	–^b^	–
**8e**	250 (58000), 265 (69000), 298 (21000), 335 (11000)	–^b^	–
**8f**	255 (85000), 285 (62000)	–^b^	–
**1**	258 (69000), 323 (11000)	–^b^	–
**3**	259 (49000), 329 (6000)	–^b^	–
**2**	259 (39000), 311 (6000)	450^c^	9900
**10a**	269 (61000), 320 (18000)	463^d^	9700
**10b**	240 (47000), 267 (29000), 298 (17000), 336 (4000), 352 (4000)	362, 377^e^	800

^a^Δ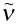 = 1/λ_max,abs_ − 1/λ_max,em_ [cm^−1^]. ^b^The residual fluorescence is only detectable in the base line, i.e., less than 5% in a.u., vide infra. ^c^λ_exc_ = 311 nm. ^d^λ_exc_ = 320 nm. ^e^λ_exc_ = 298 nm.

**Figure 6:**
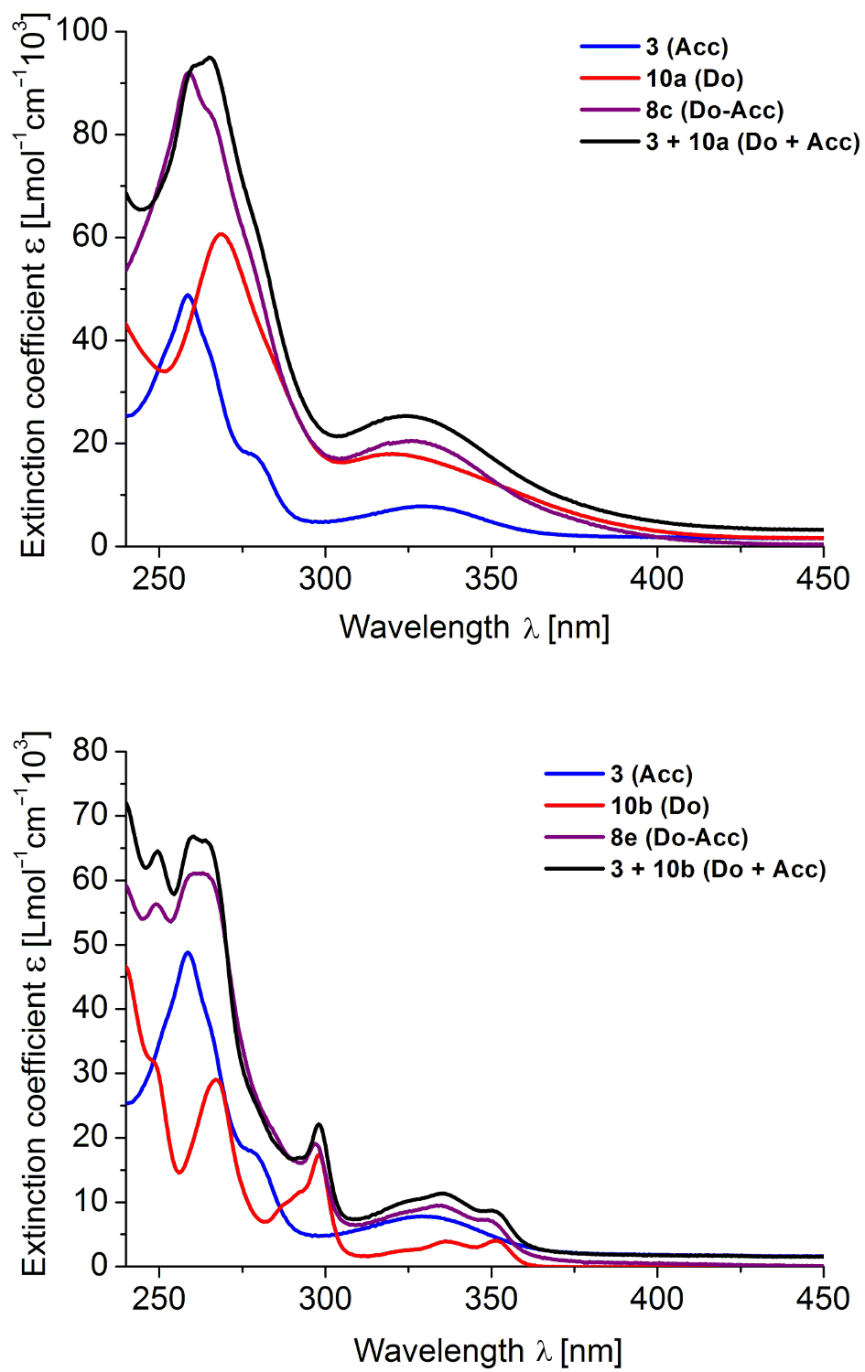
Absorption spectra of Do–anthraquinone dyads **8c** (top) and **8e** (bottom) with the corresponding references **3** and **10**, and their addition spectra (Do + Acc) (recorded in CH_2_Cl_2_, *T* = 298 K).

The extinction coefficients of the Do–anthraquinone dyads **8** are expectedly larger than those of the donor-only (**10**) or anthraquinone-only (**3**) compounds. Plotting the extinction coefficient against the wavelength it becomes evident that the Do–anthraquinone dyads behave additively with respect to the constituent reference chromophores (Figure 6). Absorption spectroscopy as a probe for the electronic ground state also supports that in Do–anthraquinone dyads **8** the donor and anthraquinone moieties are electronically decoupled in the electronic ground state.

**Figure 7:**
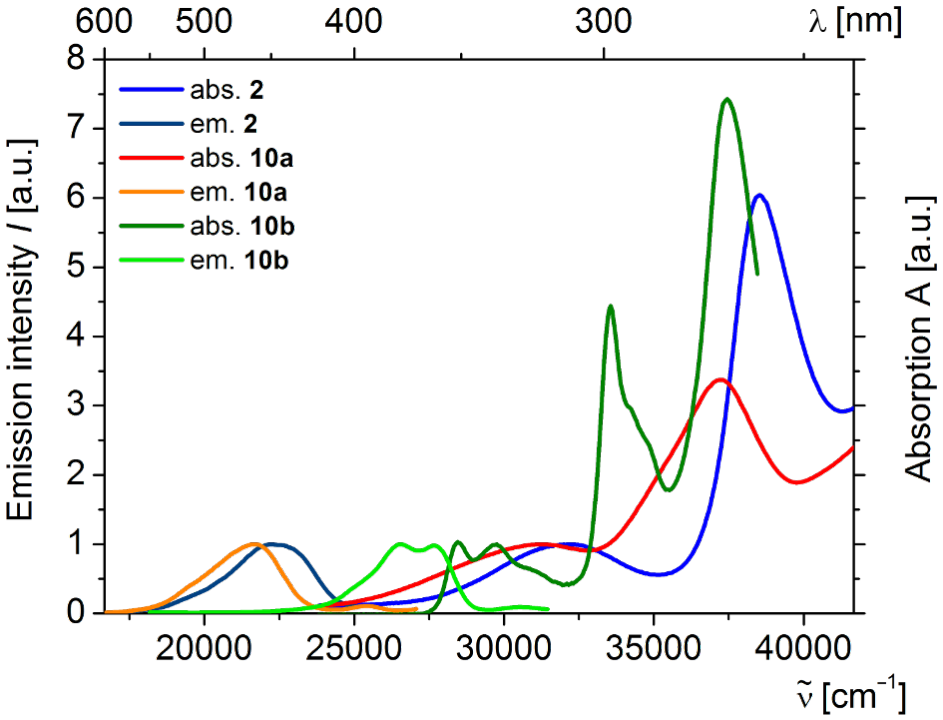
Normalized absorption and emission spectra of Ugi-donor compounds **2** and **10** (recorded in CH_2_Cl_2_, *T* = 298 K, λ_max,exc_ (**2**) = 311 nm, λ_max,exc_ (**10a**) = 320 nm, λ_max,exc_ (**10b**) = 298 nm).

**Figure 8:**
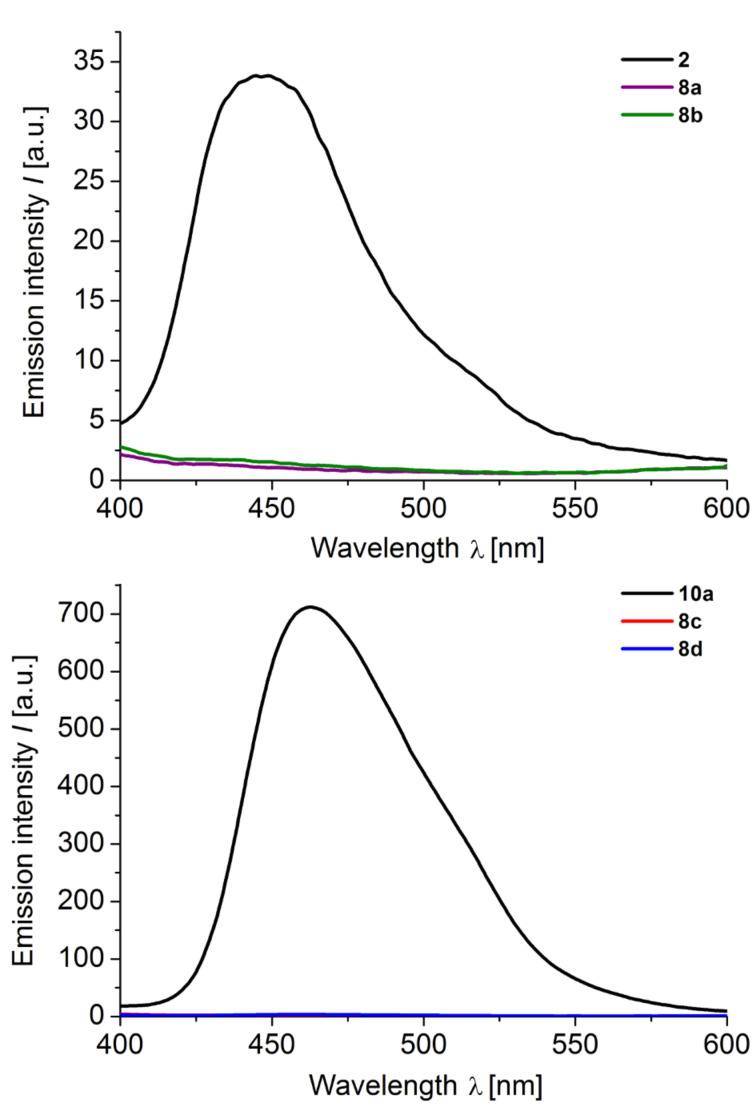
Emission spectra of donor-only system **2** phenothiazine–anthraquinone dyads **8a**,**b** (top), and the donor-only system **10a** and the phenothiazine–anthraquinone dyads **8c**,**d** (bottom) (recorded in CH_2_Cl_2_, *T* = 298 K, *c* = 0.7–2.9∙10^−6^ mol·L^−1^).

Fluorescence is an excited-state phenomenon and, therefore, steady-state emission spectra of the donor-only reference systems **2** (Figure 7) and **10** and the donor–anthraquinone dyads **8** were recorded (Figure 8). For the donor–anthraquinone dyads **8** the emission is significantly quenched in comparison to the corresponding donor-only model systems **2** and **10** at the same concentrations according to relative fluorescence quantum yields Φ_f,rel_ (Table 3) and only residual weak emission traces from the donor and/or the anthraquinone excitations can be detected (for spectra of the residual emissions of the dyads **8** see Supporting Information File 1). Since the relative fluorescence quantum yields Φ_f,rel_ are attenuated by 95–99% in comparison to the donors’ fluorescence an efficient and rapid nonradiative depopulation of the excited state can be assumed by an electron transfer. This rationale is additionally supported by our previous transient absorption spectroscopic study of the dyad **1** [56].

**Table 3:** Relative quantum yields Φ_f,rel_ of Do-anthraquinone dyads **8** in comparison to their donor-only reference systems **2** and **10**.

Donor-only reference	**2**		**10a**		**10b**	

Donor-anthraquinone dyad	**8a**	**8b**	**8c**	**8d**	**8e**	**8f**
Φ_f,rel_^a^	0.04	0.05	<0.01	<0.01	0.01	0.04

^a^Determined in CH_2_Cl_2_, *T* = 298 K, the quantum yield of the corresponding reference was set to 1.0.

According to the Weller approximation [87] the driving force for a photo-induced electron transfer leading to a charge-separated state that is responsible for the observed fluorescence quenching can be calculated from the measured electrochemical and spectroscopic data. Among several representations for calculating the Gibbs free energy of the electron transfer 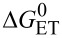 [88–89] can be described by Equation 1 [90]

[1]
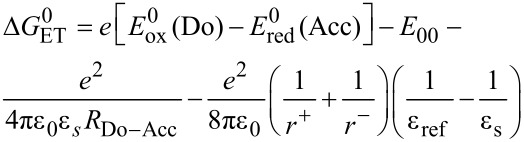


Where 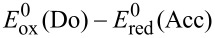 is the difference of the first oxidation potential of the donor and first reduction potential of the acceptor, respectively, *E*_00_ expresses the energy of the photonic excitation, *R*_Do–Acc_ delineates the distance between the centers of the donor and acceptor moieties, ε_s_ and ε_ref_ are the dielectric constants of the solvent applied in spectroscopy (ε_s_) and reference solvent used in electrochemistry (ε_ref_), and *r*^+^ and *r*^−^ are indicating the effective ionic radii of the donor radical cation and acceptor radical anion, respectively. It is allowed to neglect the forth term, if spectroscopic and electrochemical measurements are performed in the same solvent. Therefore, Equation 1 simplifies to Equation 2 for calculating 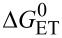


[2]
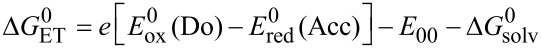


Where the first two terms indicate the free energy of the charge-separated state calculated from the spectroscopic and electrochemical measurements (in eV) and 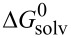 represents the correction term of the solvent polarity and the effect of the distance of the donor and acceptor moieties according to 
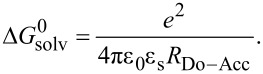


Indeed, for all Do-anthraquinone dyads **8** exergonic Gibbs free energies for the electron transfer are found, both for the simplified free enthalpy Δ*G*_ET,simpl_, i.e., the first two terms of Equation 2, and upon taking solvation into account with the term 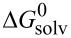. Therefore, the extent of the thermodynamically favored charge separation by an intramolecular photo-induced electron transfer (PET), plausibly explaining the observed fluorescence quenching, can be easily determined and compared within the series of related dyad systems (Table 4).

**Table 4:** Calculation of the Gibbs free energies for the simple electron transfer Δ*G*_ET,simpl_, for the solvation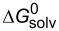, and the electron transfer 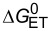 according to the Weller approximation of the dyads **8**.

Compound	*e*[*E*^0^_ox_ (Do) − *E*^0^_red_ (Acc)]	*E*_00_	*∆G*_ET,simpl_	*R*_Do−Acc_	Δ*G*^0^_solv_	Δ*G*^0^_ET_
	[eV]^a^	[eV]^b^	[eV]^c^	[nm]^d^	[eV]	[eV]

**8a**	1.60	3.08	−1.46	0.39	0.40	−1.78
**8b**	1.65	3.08	−1.43	0.43	0.37	−1.66
**8c**	1.51	2.93	−1.42	0.53	0.30	−1.68
**8d**	1.61	2.93	−1.32	0.38	0.41	−1.64
**8e**	2.16	3.49	−1.34	0.40	0.39	−1.70
**8f**	2.09	3.49	−1.41	0.46	0.34	−1.69

^a^Calculated from (*E*_1/2_^0/+1^ − *E*_1/2_^−1/0^) [V] obtained from cyclic voltammetry (see Table 1). ^b^*E*_00_ [eV] of donor-only reference compounds **2** (*E*_00_ = 402 nm), **10a** (*E*_00_ = 423 nm), and **10b** (*E*_00_ = 355 nm) were estimated by the intersection of normalized absorption and emission spectra. ^c^*∆G*_ET,simpl_ = *e*[*E*^0^_ox_ (Do) − *E*^0^_red_ (Acc)] − *E*_00_. ^d^The distances *R*_Do−Acc_ [nm] of the donor and acceptor centers were estimated from lowest energy conformers by optimized MM2FF calculations [91] taking the distances between the centroid of the anthraquinone and the nitrogen atom of the donor moiety. For the diphenothiazine derivative **8d** the more electron-rich inner phenothiazine was assumed to be oxidized first.

The negative free enthalpies of PET are numerically very similar for **8b**, **8c**, **8e**, and **8f**, however, larger in quantity for dyad **8a** and smaller for dyad **8d**. A diminishing of the distance between donor and anthraquinone, e.g., by adopting flexible close-contact conformations as for dyad **8a** causes an increase in the driving force of the PET. Smaller excitation energies *E*_00_ and lower oxidation potentials as for the diphenothiazine dyad **8d** cause a smaller PET driving force 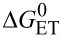. All phenothiazine systems **8a–d** are excited at longer wavelengths, i.e., at lower energies, than the carbazole dyads **8e** and **8f**. Eventually, the absorption characteristics of phenothiazine dyads can be more easily red-shifted and, therefore, charge separation by PET should be accessible with visible light by fine-tuning the donor chromophore towards lower HOMO–LUMO gaps.

## Conclusion

The Ugi four-component reaction represents a rapid and excellent modular and diversity-oriented synthesis of donor-anthraquinone dyads with various phenothiazine and carbazole model donors. Cyclic voltammetry and UV–vis spectroscopy clearly indicate an electronic decoupling of the donor and the acceptor substituents in the electronic ground state, whereas the emission of the donor moieties is efficiently quenched according to static fluorescence spectroscopy. The observed peculiar fluorescence quenching was previously studied by femtosecond transient absorption spectroscopy of a related model dyad indicating a photo-induced electron transfer (PET) process into a dark, i.e., non-emissive, charge-separated state. The Gibbs free energies of the PET into the charge-separated states are exergonic and can be quickly calculated from absorption and electrochemical data applying the Weller approximation. The concise synthetic concept to donor–acceptor systems is very general, easy to perform and readily expandable to all kinds of functional π-electron systems. The Weller approximation of the Gibbs free energies of the PET allows a semiquantitative evaluation and optimization of photo-induced charge-separation systems. Studies directed towards multicomponent syntheses of more complex light harvesting and charge separation systems are currently underway.

## Experimental

### Synthesis of compounds **8** and **10** via Ugi four-component reaction (General Procedure) in a manner similar to [56])

In a 25 mL Schlenk tube 0.50 mmol of the donor hydrochloride **4** and potassium hydroxide (28 mg, 0.50 mmol) were dissolved in methanol and the mixture was stirred for 30 min (for experimental details see Table 5). A solution of aldehyde **5** (118 mg, 0.50 mmol) in dichloromethane (2 mL) or the neat aldehyde **9** (22 mg, 0.50 mmol) were added to the reaction mixture and the solution was stirred at rt for 1 h, followed by the addition of 1 equiv of acetic acid (**6**) (30 mg, 0.50 mmol) and 1 equiv of *tert*-butyl isocyanide (**7**) (42 mg, 0.50 mmol) by syringe. The reaction mixture was stirred overnight at rt. The solvents were removed in vacuo and the crude product was purified by column chromatography on silica gel to give the analytically pure Ugi products **8** and **10**.

**Table 5:** Experimental details for the synthesis of the Ugi products **8** and **10**.

Entry	MeOH [mL]	CH_2_Cl_2_ [mL]	Reaction time *t* [d]	Ugi 4CR products **8** or **10** (yield)

1	3	2	2	153 mg (50 %) of **8a**
2	2	2	1	193 mg(57 %) of **8b**
3	2	2	1	299 mg(80 %) of **8c**
4	2	2	1	287 mg (60 %) of **8d**
5	2	2.2	1	187 mg (55 %) of **8e**
6	2	2	1	162 mg (45 %) of **8f**
7	3	–	1	105 mg (35 %) of **10a**
8	2	–	1	162 mg (72 %) of **10b**

## Supporting Information

File 1 ^1^H NMR, ^13^C NMR, UV–vis, fluorescence spectra and cyclic voltammograms of compounds **8** and **10**, a summary of the X-ray crystallographic data of S(O)-**1**, computed xyz-coordinates of the structure **1** and HOMO and LUMO energies.
