# Supplementary material for: The Ugi four-component reaction as a concise modular synthetic tool for photo-induced electron transfer donor-anthraquinone dyads
Source: Beilstein J Org Chem. 2014 May 5;10:1006–16. doi: 10.3762/bjoc.10.100 (PMC4077531; doi:10.3762/bjoc.10.100)
Supplement: File 1 — 1H NMR, 13C NMR, UV–vis, fluorescence spectra and cyclic voltammograms of compounds 8 and 10, a summary of the X-ray crystallographic data of S(O)-1, computed xyz-coordinates of the structure 1 and HOMO and LUMO energies. [file Beilstein_J_Org_Chem-10-1006-s001.pdf]

Supporting Information

for

The Ugi four-component reaction as a concise modular synthetic tool for photo-induced electron transfer donor-anthraquinone dyads

Sarah Bay[1], Gamall Makhloufi[2], Christoph Janiak[2], and Thomas J. J. Müller[1,*]

Address: [1]Heinrich-Heine Universität Düsseldorf, Institut für Organische Chemie und Makromolekulare Chemie, Universitätsstraße 1, D-40225 Düsseldorf, Germany and [2]Heinrich-Heine Universität Düsseldorf, Institut für Anorganische Chemie und Strukturchemie, Universitätsstraße 1, D-40225 Düsseldorf, Germany

Email: Thomas J. J. Müller - ThomasJJ.Mueller@uni-duesseldorf.de

*Corresponding author

^{1}H NMR, ^{13}C NMR, UV–vis, fluorescence spectra and cyclic voltammograms of compounds **8** and **10**, a summary of the X-ray crystallographic data of S(O)-**1**, computed xyz-coordinates of the structure **1** and HOMO and LUMO energies.

Table of Contents

1 General considerations S3

2 References S5

3 Analytical data of Ugi compounds **8** and **10** S6

3.1 2-(*N*-(3-(10*H*-Phenothiazin-10-yl)propyl)acetamido)-*N*-(*tert*-butyl)-2-(9,10-dioxo-9,10-dihydroanthracen-2-yl) acetamide (**8a**) S6

3.2 2-(*N*-(4-((10*H*-Phenothiazin-10-yl)methyl)benzyl)acetamido)-*N*-(*tert*-butyl)-2-(9,10-dioxo-9,10-dihydroanthracen-2-yl) acetamide (**8b**) S9

3.3 *N*-(*tert*-Butyl)-2-(9,10-dioxo-9,10-dihydroanthracen-2-yl)-2-(*N*-(4-(10-hexyl-10*H*-phenothiazin-3-yl)benzyl)acetamido) acetamide (**8c**) S12

3.4 *N*-(*tert*-Butyl)-2-(*N*-((10,10'-dihexyl-10*H*,10'*H*-[3,3'-biphenothiazin]-7-yl)methyl)acetamido)-2-(9,10-dioxo-9,10-dihydroanthracen-2-yl) acetamide (**8d**) S15

3.5 *N*-(*tert*-Butyl)-2-(9,10-dioxo-9,10-dihydroanthracen-2-yl)-2-(*N*-((9-hexyl-9*H*-carbazol-3-yl)methyl)acetamido) acetamide (**8e**) S18

3.6 *N*-(*tert*-Butyl)-2-(9,10-dioxo-9,10-dihydroanthracen-2-yl)-2-(*N*-(4-(9-hexyl-9*H*-carbazol-3-yl)benzyl)acetamido) acetamide (**8f**) 21

3.7 *N*-(*tert*-Butyl)-2-(*N*-(4-(10-hexyl-10*H*-phenothiazin-3-yl)benzyl)-acetamido) propanamide (**10a**) 24

3.8 *N*-(*tert*-Butyl)-2-(*N*-((9-hexyl-9*H*-carbazol-3-yl)methyl)-acetamido) propanamide (**10b**) S27

4 Cyclic voltammetry of Ugi compounds **8** and **10** S30

4.1 2-(*N*-(3-(10*H*-Phenothiazin-10-yl)propyl)acetamido)-*N*-(*tert*-butyl)-2-(9,10-dioxo-9,10-dihydroanthracen-2-yl) acetamide (**8a**) S30

4.2 2-(*N*-(4-((10*H*-Phenothiazin-10-yl)methyl)benzyl)acetamido)-*N*-(*tert*-butyl)-2-(9,10-dioxo-9,10-dihydroanthracen-2-yl) acetamide (**8b**) S30

4.3 *N*-(*tert*-Butyl)-2-(9,10-dioxo-9,10-dihydroanthracen-2-yl)-2-(*N*-(4-(10-hexyl-10*H*-phenothiazin-3-yl)benzyl)acetamido) acetamide (**8c**) S31

4.4 *N*-(*tert*-Butyl)-2-(*N*-((10,10'-dihexyl-10*H*,10'*H*-[3,3'-biphenothiazin]-7-yl)methyl)acetamido)-2-(9,10-dioxo-9,10-dihydroanthracen-2-yl) acetamide (**8d**) S31

4.5 *N*-(*tert*-Butyl)-2-(9,10-dioxo-9,10-dihydroanthracen-2-yl)-2-(*N*-((9-hexyl-9*H*-carbazol-3-yl)methyl)acetamido) acetamide (**8e**) S32

4.6 *N*-(*tert*-Butyl)-2-(9,10-dioxo-9,10-dihydroanthracen-2-yl)-2-(*N*-(4-(9-hexyl-9*H*-carbazol-3-yl)benzyl)acetamido) acetamide (**8f**) S32

4.7 *N*-(*tert*-Butyl)-2-(*N*-(4-(10-hexyl-10*H*-phenothiazin-3-yl)benzyl)-acetamido) propanamide (**10a**) S33

4.8 *N*-(*tert*-Butyl)-2-(*N*-((9-hexyl-9*H*-carbazol-3-yl)methyl)acetamido) propanamide (**10b**) S33

5 Absorption spectroscopy of compounds **8** and **10** S34

5.1 2-(*N*-(3-(10*H*-Phenothiazin-10-yl)propyl)acetamido)-*N*-(*tert*-butyl)-2-(9,10-dioxo-9,10-dihydroanthracen-2-yl) acetamide (**8a**) S34

5.2 2-(*N*-(4-((10*H*-Phenothiazin-10-yl)methyl)benzyl)acetamido)-*N*-(*tert*-butyl)-2-(9,10-dioxo-9,10-dihydroanthracen-2-yl) acetamide (**8b**) S34
5.3 *N*-(*tert*-Butyl)-2-(9,10-dioxo-9,10-dihydroanthracen-2-yl)-2-(*N*-(4-(10-hexyl-10*H*-phenothiazin-3-yl)benzyl)acetamido) acetamide (**8c**) S35
5.4 *N*-(*tert*-Butyl)-2-(*N*-((10,10'-dihexyl-10*H*,10'*H*-[3,3'-biphenothiazin]-7-yl)methyl)acetamido)-2-(9,10-dioxo-9,10-dihydroanthracen-2-yl) acetamide (**8d**) S35
5.5 *N*-(*tert*-Butyl)-2-(9,10-dioxo-9,10-dihydroanthracen-2-yl)-2-(*N*-((9-hexyl-9*H*-carbazol-3-yl)methyl)acetamido) acetamide (**8e**) S36
5.6 *N*-(*tert*-Butyl)-2-(9,10-dioxo-9,10-dihydroanthracen-2-yl)-2-(*N*-(4-(9-hexyl-9*H*-carbazol-3-yl)benzyl)acetamido) acetamide (**8f**) S36
6 Emission Spectroscopy of **2**, **10** and **8** S37
6.1 *N*-(*tert*-Butyl)-2-(*N*-((10-hexyl-10*H*-phenothiazin-3-yl)methyl)-acetamido) propanamide (**2**) 37
6.2 2-(*N*-(3-(10*H*-Phenothiazin-10-yl)propyl)acetamido)-*N*-(*tert*-butyl)-2-(9,10-dioxo-9,10-dihydroanthracen-2-yl) acetamide (**8a**) S37
6.3 2-(*N*-(4-((10*H*-Phenothiazin-10-yl)methyl)benzyl)acetamido)-*N*-(*tert*-butyl)-2-(9,10-dioxo-9,10-dihydroanthracen-2-yl) acetamide (**8b**) S38
6.4 *N*-(*tert*-Butyl)-2-(*N*-(4-(10-hexyl-10*H*-phenothiazin-3-yl)benzyl)-acetamido) propanamide (**10a**) S38
6.5 *N*-(*tert*-Butyl)-2-(9,10-dioxo-9,10-dihydroanthracen-2-yl)-2-(*N*-(4-(10-hexyl-10*H*-phenothiazin-3-yl)benzyl)acetamido) acetamide (**8c**) S39
6.6 *N*-(*tert*-Butyl)-2-(*N*-((10,10'-dihexyl-10*H*,10'*H*-[3,3'-biphenothiazin]-7-yl)methyl)acetamido)-2-(9,10-dioxo-9,10-dihydroanthracen-2-yl) acetamide (**8d**) S39
6.7 *N*-(*tert*-Butyl)-2-(*N*-((9-hexyl-9*H*-carbazol-3-yl)methyl)acetamido) propanamide (**10b**) S40
6.8 *N*-(*tert*-Butyl)-2-(9,10-dioxo-9,10-dihydroanthracen-2-yl)-2-(*N*-((9-hexyl-9*H*-carbazol-3-yl)methyl)acetamido) acetamide (**8e**) S40
6.9 *N*-(*tert*-Butyl)-2-(9,10-dioxo-9,10-dihydroanthracen-2-yl)-2-(*N*-(4-(9-hexyl-9*H*-carbazol-3-yl)benzyl)acetamido) acetamide (**8f**) S41
7 Crystallographic Data of S(O)-1 .... S42
8 XYZ-coordinates and FMO energies of compound **1** S55

1 General considerations

Commercial grade reagents were used as supplied without further purification and were purchased from abcr GmbH & Co. KG, Acros Organics, Alfa Aesar GmbH & Co. KG, Sigma-Aldrich Chemie GmbH.

The purification of Ugi compounds was performed column chromatographically on silica gel 60 M (0.04–0.063 mm) from Macherey-Nagel GmbH & Co. KG using flash technique under pressure of 2 bar. The crude mixtures were adsorbed on Celite® 545 from Carl Roth GmbH Co. KG before chromatographic purification. The reaction progress was monitored

qualitatively using TLC Silica gel 60 F254 aluminium sheets obtained from Merck KGaA, Darmstadt. The spots were detected with UV light at 254 nm and using an iodine chamber.

^{1}H, ^{13}C, and 135-DEPT ^{13}C NMR spectra were recorded on Bruker Avance DRX 500 or Bruker AV III 600. $CDCl_3$, and CD_2Cl_2 were used as deuterated solvents. The resonances of the solvents were locked as internal standards ($CDCl_3$: ^{1}H δ 7.24, ^{13}C δ 77.2; CD_2Cl_2: ^{1}H δ 5.32, ^{13}C δ 54.0). The muliplicities of the signals were abbreviated as follows: s: singlet; d: doublet; dd: doublet of doublets; t: triplet; m: multiplet. The type of carbon atoms was determined on the basis of 135-DEPT ^{13}C NMR spectra. For the description of the ^{13}C NMR spectra primary carbon nuclei are abbreviated with CH_3, secondary carbon nuclei with CH_2, tertiary carbon nuclei with CH, and quaternary carbon nuclei with $C_{quat.}$.

MALDI mass spectra were measured on a Bruker Ultraflex spectrometer, ESI mass spectra were measured on an Ion-Trap-API-mass spectrometer of Finnigan LCQ Deca (Thermo Quest).

IR spectra were obtained on a Bruker Vector 22 FT-IR using a potassium bromide pellet or on a Shimadzu IRAffinity-1 which works with the attenuated total reflection (ATR) method. The intensity of signals is abbreviated as follows: s (strong), m (medium), w (weak). The melting points (uncorrected) were measured on Büchi Melting Point B-540.

UV–vis spectra were recorded on 84252 Diode Array spectrometer by Hewlett Packard in dichloromethane at T = 293 K.

Fluorescence spectra were recorded on Perkin Elmer LS55 in dichloromethane and concentrations of 10^{-6} $molL^{-1}$. Data analysis was done with the software FL Winlab by Perkin Elmer.

Combustion analyses were carried out on Perkin Elmer Series II Analyser 2400 in the micro analytical laboratory of the Institut für Pharmazeutische und Medizinische Chemie der Heinrich-Heine-Universität Düsseldorf.

Cyclic voltammetry experiments were performed with 263A E&G Princeton Applied Research as potentiostatic instrumentation under argon in dry and degassed dichloromethane at T = 298 K and at scan rates of 100, 250, 500 and 1000 mVs^{-1}. The electrolyte was tetrabutylammonium hexafluorophosphate at a concentration of c = 0.1 $molL^{-1}$. The working electrode was a 1 mm platinum disk, the counter electrode was a platinum wire and the reference electrode was a silver/silverchloride electrode filled with aqueous saturated sodium chloride solution. The potentials were calibrated using $[FeCp_2]/[FeCp_2]^+$ ($E_0^{0/+1}$ = 450 mV)[1] as an internal potential standard.

X-ray crystallography

Single-crystals of S(O)-**1** were carefully selected under a polarizing microscope and mounted on a loop. Data collection: Bruker Kappa APEX2 CCD diffractometer with microfocus sealed tube, MoKα radiation (λ = 0.71073 Å), multi-layer mirror system, ω-scans. Data collection and

cell refinement with APEX2,[2] data reduction with SAINT (Bruker).[2] Structure analysis and refinement: The structures were solved by direct methods (SHELXS-97),[3] refinement was done by full-matrix least squares on F^2 using the SHELXL-97 program suite,[3] empirical (multi-scan) absorption correction with SADABS (Bruker).[4] All non-hydrogen positions were refined with anisotropic temperature factors. Hydrogen atoms for aromatic CH, aliphatic CH, CH_2 and CH_3 groups were positioned geometrically. The (N-)H atom which was found and refined. The structure contains a partially (0.88) occupied water molecule disordered over two positions. The H atoms of this crystal water could not be located. Graphics were drawn with DIAMOND (Version 3.2).[5] Computations on the supramolecular interactions were carried out with PLATON for Windows.[6] The structural data for this paper has been deposited with the Cambridge Crystallographic Data Center (CCDC-number 976231). These data can be obtained free of charge via www.ccdc.cam.ac.uk/data_request/cif.

2 References

[1] Zanello, P. in *Ferrocenes* (Togni, A.; Hayashi, T., eds), VCH, Weinheim, **1995**, pp. 317-430.

[2] Apex2, Data Collection Program for the CCD Area-Detector System; SAINT, Data Reduction and Frame Integration Program for the CCD Area-Detector System. Bruker Analytical X-ray Systems, Madison, Wisconsin, USA, **1997-2006**.

[3] Sheldrick, G. M. *Acta Crystallogr. A* **2008**, *64*, 112-122.

[4] Sheldrick, G. Program SADABS: Area-detector absorption correction, University of Göttingen, Germany, **1996**.

5 Brandenburg, K. Diamond (Version 3.2), Crystal and Molecular Structure Visualization, Crystal Impact – K. Brandenburg & H. Putz Gbr, Bonn (Germany) **2009**.

[6] a) Spek, A. L. *Acta Crystallogr. D* **2009**, *65*, 148-155. b) Spek, A. L. PLATON – A Multipurpose Crystallographic Tool, Utrecht University, Utrecht, The Netherlands, **2008**; Windows implementation: L. J. Farrugia, University of Glasgow, Scotland, Version 40608, **2008**.

3 Analytical data of Ugi compounds 8 and 10

3.1 2-(*N*-(3-(10*H*-Phenothiazin-10-yl)propyl)acetamido)-*N*-(*tert*-butyl)-2-(9,10-dioxo-9,10-dihydroanthracen-2-yl) acetamide (8a)

Purification by column chromatography on silica gel (*n*-hexane/ethyl acetate 1:1) gave 153 mg (50%) of compound **8a** as a red solid.

Mp 204 °C. R_f (*n*-hexane/ethyl acetate 1:1) = 0.13. ^{1}H NMR (500 MHz, CD_2Cl_2): δ = 1.33 (s, 9 H), 1.56-1.66 (m, 2 H), 1.92 (s, 3 H), 3.42-3.55 (m, 2 H), 3.68-3.86 (m, 2 H), 5.75 (s, 1 H), 6.08 (s, 1 H), 6.76 (d, *J* = 8.2 Hz, 2 H), 6.84 (t, *J* = 7.4 Hz, 2 H), 7.04-7.13 (m, 4 H), 7.70 (d, *J* = 8.1 Hz, 1 H), 7.79-7.90 (m, 2 H), 8.10-8.22 (m, 2 H), 8.25-8.33 (m, 2 H). ^{13}C NMR (125.8 MHz, CD_2Cl_2): δ = 21.7 (CH_3), 27.4 (CH_2), 28.9 ($3CH_3$), 44.6 (CH_2), 46.3 (CH_2), 52.1 (C_{quat}), 63.4 (CH); 116.2 (CH), 123.3 (CH), 126.5 (C_{quat}), 127.59 (CH), 127.64 (CH), 127.8 (CH), 127.9 (CH), 128.0 (CH), 128.1 (CH), 133.4 (C_{quat}), 134.0 (C_{quat}), 134.05 (C_{quat}), 134.1 (C_{quat}), 134.7 (CH), 134.77 (CH), 134.8 (CH), 143.4 (C_{quat}), 145.5 (C_{quat}), 168.6 (C_{quat}), 172.3 (C_{quat}), 183.1 (2 C_{quat}). IR (KBr) $\tilde{\nu}$ [cm^{-1}] = 3287 (w), 3059 (w), 2961 (w), 1672 (m), 1624 (s), 1591 (m), 1572 (w), 1547 (m), 1458 (s), 1429 (m), 1383 (w), 1366 (m), 1325 (m), 1296 (s), 1263 (m), 1227 (m), 1211 (m), 1173 (w), 1128 (m) 1109 (w), 1057 (w), 1040 (m), 951 (w), 932 (m), 907 (w), 857 (w), 800 (w), 754 (s), 729 (w), 708 (s), 675 (w), 634 (w). UV–vis (CH_2Cl_2) λ_{max} (ε) [nm] = 257 (43600), 319 (5100). MALDI-MS: *m*/*z* = 617.1 ($[M]^+$). Anal. calcd. for $C_{37}H_{35}N_3O_4S$ (617.2) : C 71.94, H 5.71, N 6.80; Found: C 71.86, H 5.52, N 6.71.

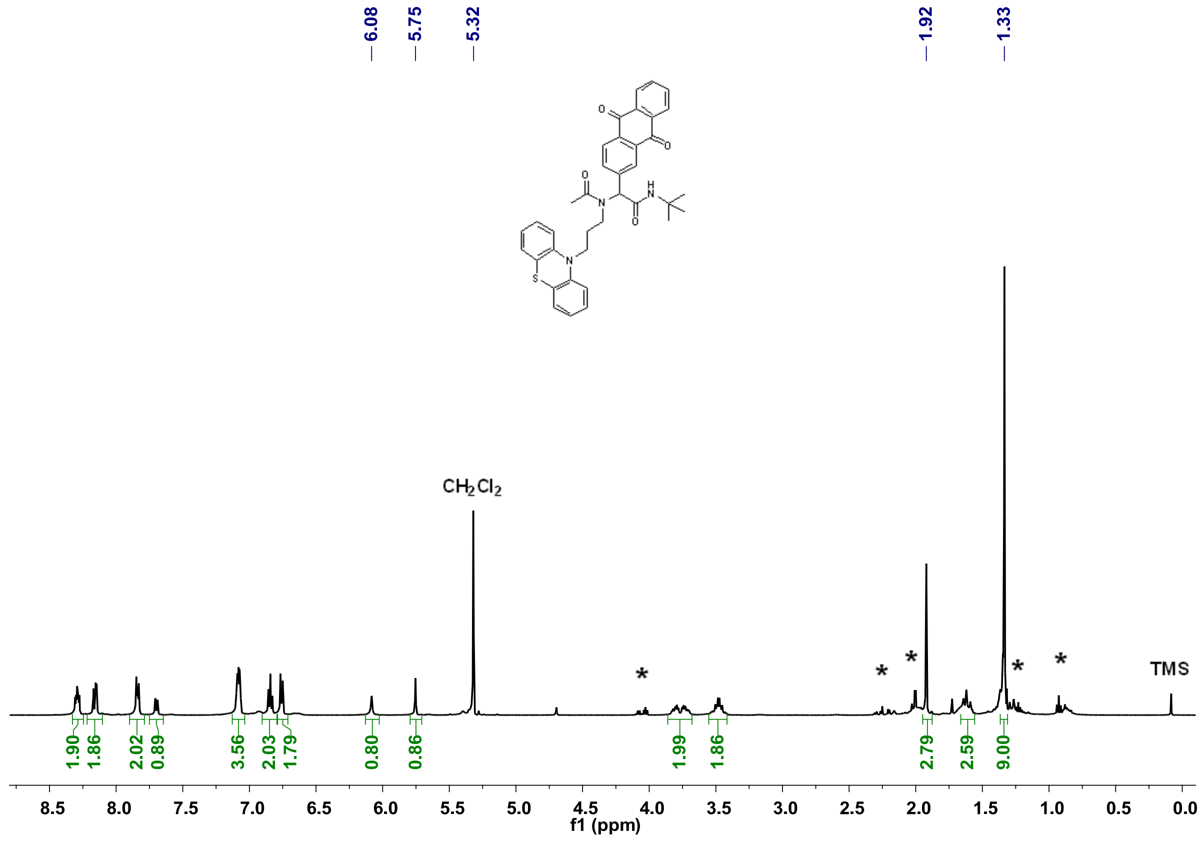

^{1}H NMR of **8a** (CD_2Cl_2, 298 K, 500 MHz, δ in ppm). * Impurities from residual solvents.

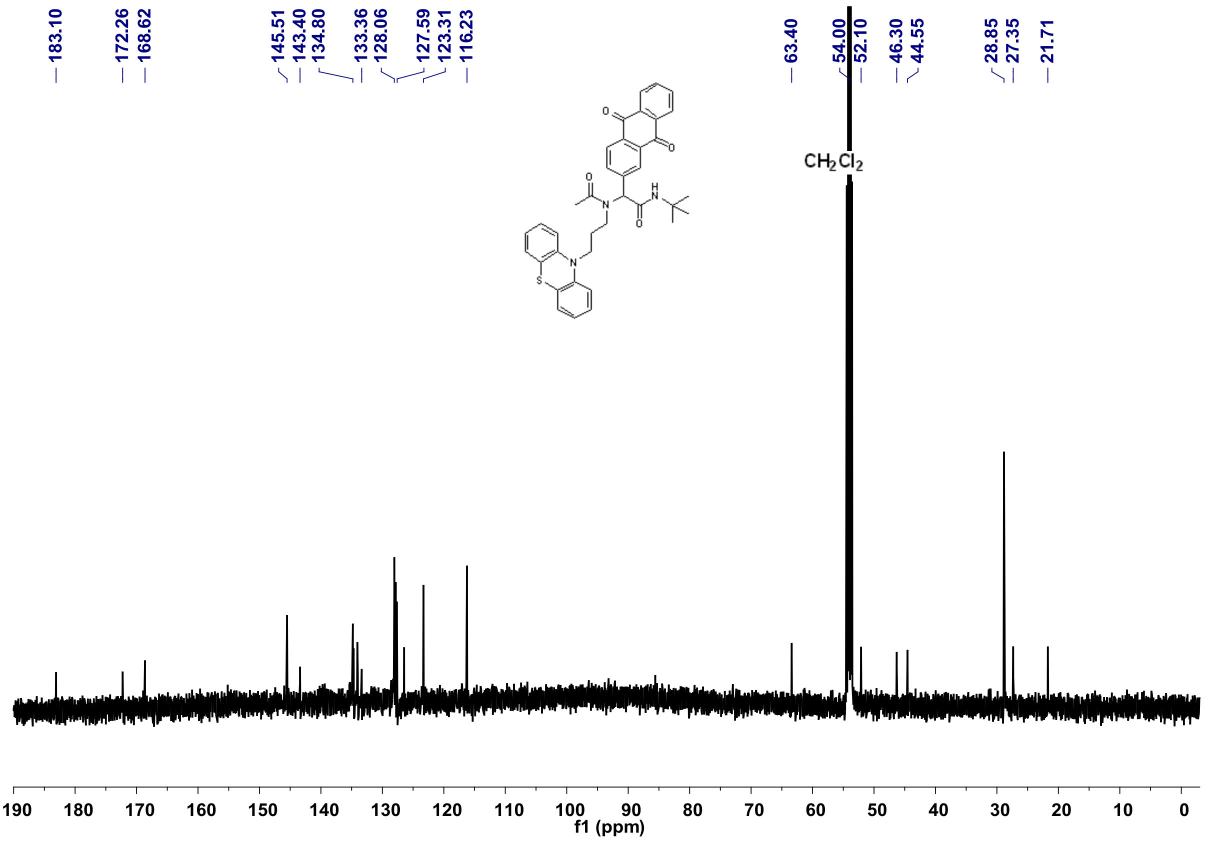

^{13}C NMR of **8a** (CD_2Cl_2, 298 K, 126 MHz, δ in ppm).

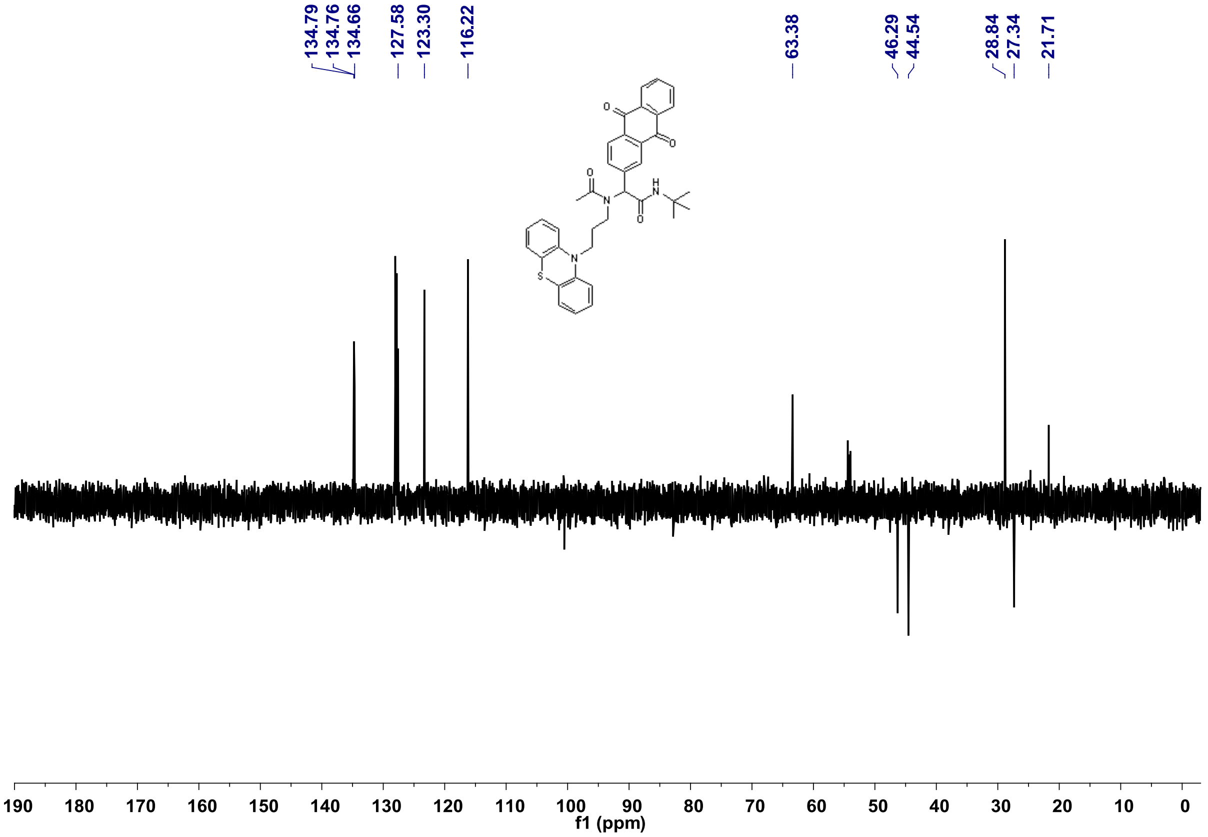

135-DEPT of **8a** (CD_2Cl_2, 298 K, 126 MHz, δ in ppm).

3.2 2-(*N*-(4-((10*H*-Phenothiazin-10-yl)methyl)benzyl)acetamido)-*N*-(*tert*-butyl)-2-(9,10-dioxo-9,10-dihydroanthracen-2-yl) acetamide (8b)

Purification by column chromatography on silica gel (*n*-hexane/ethyl acetate 2:1) gave 193 mg (57%) of compound **8b** as a light brown solid.

Mp 177 °C. R_f (*n*-hexane/ethyl acetate 2:1) = 0.08. ^{1}H NMR (500 MHz, $CDCl_3$): δ = 1.30 (s, 9 H), 2.16 (s, 3 H), 4.61 (d, J = 17.7 Hz, 1 H), 4.79 (d, J = 17.7 Hz, 1 H), 4.87 (s, 2 H), 6.02 (s, 1 H), 6.10 (s, 1 H), 6.44 (d, J = 8.1 Hz, 2 H), 6.79 (t, J = 7.4 Hz, 2 H), 6.88 (t, J = 7.6 Hz, 2 H), 6.94 (d, J = 7.8 Hz, 2 H), 7.01 (d, J = 7.4 Hz, 2 H), 7.08 (d, J = 7.8 Hz, 2H), 7.71 (d, J = 7.8 Hz, 1H), 7.74-7.79 (m, 2 H), 8.09-8.16 (m, 2 H), 8.20-8.24 (m, 2 H). ^{13}C NMR (125.8 MHz, $CDCl_3$): δ = 22.7 (CH_3), 28.8 (CH_3), 51.1 (CH_2), 52.1 (C_{quat}), 52.3 (CH_2), 62.8 (CH), 115.5 (CH), 122.7 (CH), 123.4 (C_{quat}), 126.8 (CH), 127.0 (CH), 127.3 (CH), 127.4 (CH), 127.7 (CH), 128.0 (CH), 133.0 (C_{quat}), 133.50 (C_{quat}), 133.53 (C_{quat}), 134.5 (CH), 134.7 (CH), 136.0 (C_{quat}), 142.5 (C_{quat}), 144.5 (C_{quat}), 168.0 (C_{quat}), 172.8 (C_{quat}), 182.6 (C_{quat}), 182.7 (C_{quat}). IR (KBr) $\tilde{\nu}$ [cm^{-1}] = 3311 (m), 2959 (w), 1736 (w), 1677 (s), 1619 (s), 1560 (m), 1543 (m), 1509 (w), 1491 (w), 1409 (s), 1359 (s), 1324 (s), 1294 (s), 1247 (m), 1209 (s), 1052 (w), 971 (w), 931 (m), 858 (m), 794 (w), 747 (m), 712 (m), 679 (w), 556 (w). UV–vis (CH_2Cl_2) λ_{max} (ε) [nm] = 258 (86600), 325 (10000). MALDI-MS: m/z = 679.2 ([M]$^+$). Anal. calcd. for $C_{42}H_{37}N_3O_4S$ (679.3): C 74.20, H 5.49, N 6.18; Found: C 74.42, H 5.72, N 5.89.

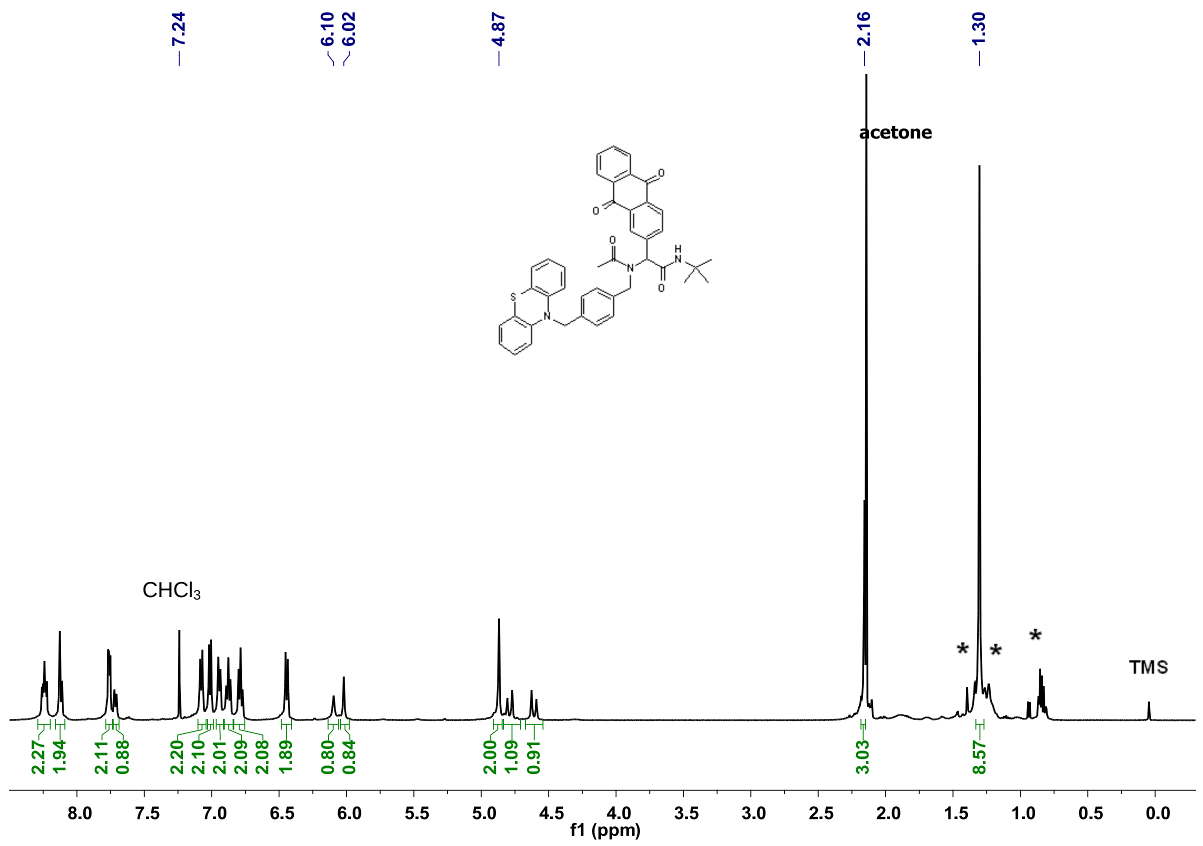

^{1}H NMR of **8b** ($CDCl_3$, 298 K, 500 MHz, δ in ppm). * Impurities from residual solvents.

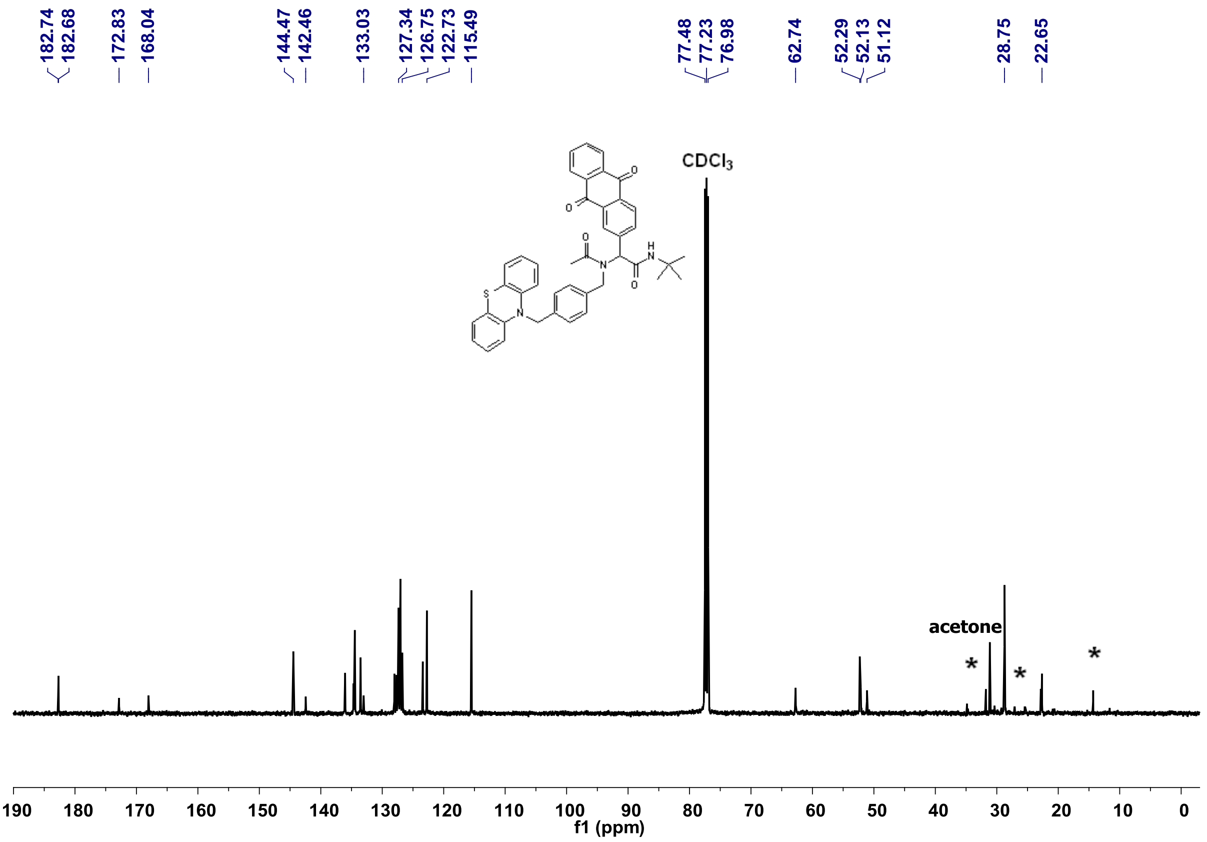

^{13}C NMR of **8b** ($CDCl_3$, 298 K, 126 MHz, δ in ppm).

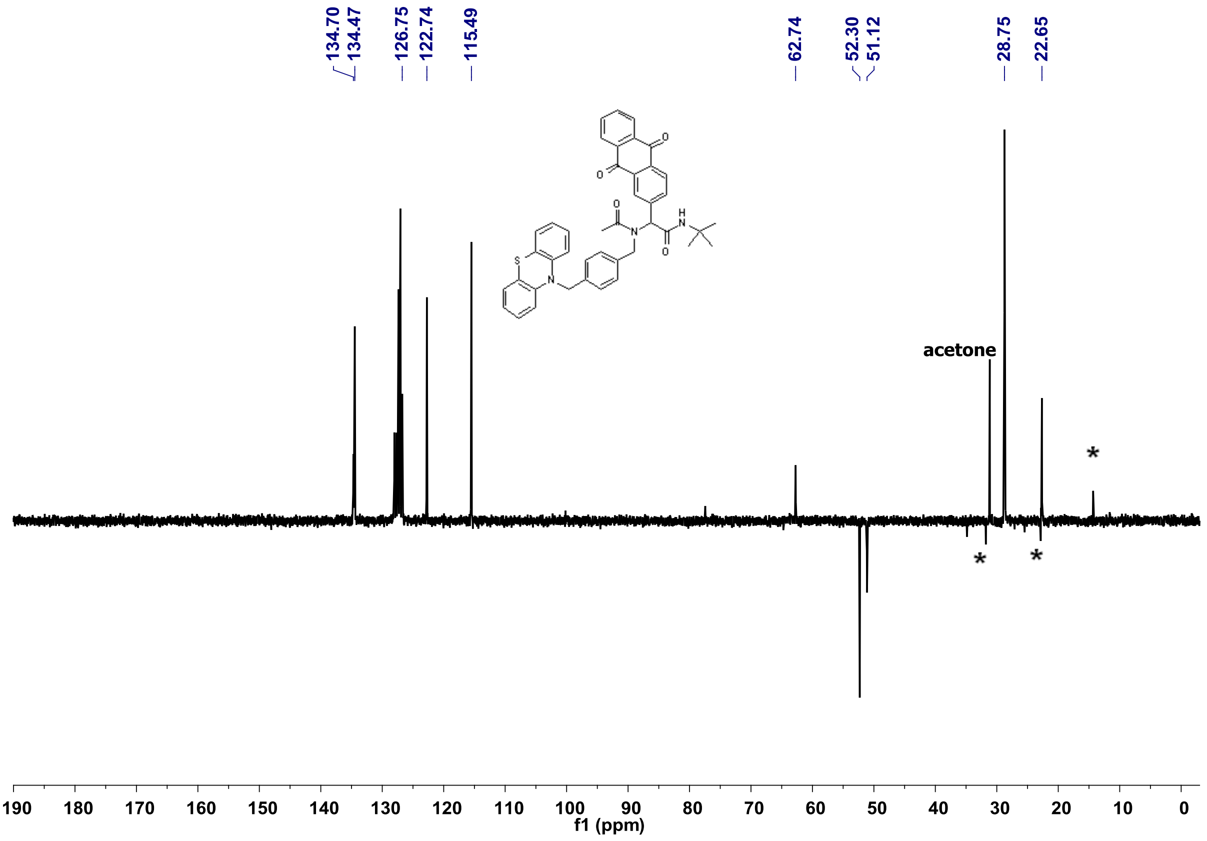

135-DEPT of **8b** ($CDCl_3$, 298 K, 126 MHz, δ in ppm).

3.3 *N*-(*tert*-Butyl)-2-(9,10-dioxo-9,10-dihydroanthracen-2-yl)-2-(*N*-(4-(10-hexyl-10*H*-phenothiazin-3-yl)benzyl)acetamido) acetamide (8c)

Purification by column chromatography on silica gel (*n*-hexane/ethyl acetate 2:1) gave 299 mg (80%) of compound **8c** as a deep red solid.

Mp 146 °C. R_f (*n*-hexane/ethyl acetate 2:1) = 0.11. ^{1}H NMR (500 MHz, $CDCl_3$): δ = 0.85 (t, *J* = 7.0 Hz, 3 H), 1.26-1.31 (m, H), 1.35 (s, 9 H), 1.38-1.43 (m, 2 H), 1.68-1.86 (m, 2 H), 2.24 (s, 3 H), 3.69-3.94 (m, 2 H), 4.61 (d, *J* = 17.5 Hz, 1 H), 4.84 (d, *J* = 17.3 Hz, 1 H), 5.97 (s, 1 H); 6.10 (s, 1 H), 6.73-6.81 (m, 1 H), 6.83-6.99 (m, 4 H), 7.04 (s, 1 H), 7.07-7.17 (m, 3 H), 7.20 (d, *J* = 7.5 Hz, 2 H), 7.59-7.63 (m, 1 H), 7.65 (td, *J* = 1.6 Hz, *J* = 7.5 Hz, 1 H), 7.81 (d, *J* = 7.6 Hz, 1 H), 8.01 (s, 1 H), 8.10-8.16 (m, 2 H), 8.17-8.22 (m, 1 H). ^{13}C NMR (125.8 MHz, $CDCl_3$): δ = 14.2 (CH_3), 22.7 (CH_3), 22.8 (CH_2), 26.9 (CH_2), 28.8 (CH_3), 31.7 (CH_2), 47.9 (CH_2), 51.0 (CH_2), 52.2 (C_{quat}), 62.4 (CH), 115.8 (CH), 122.8 (CH), 125.4 (C_{quat}), 125.7 (CH), 126.6 (CH), 126.86 (CH), 126.91 (CH), 127.3 (CH), 127.4 (CH), 127.5 (CH), 127.8 (CH), 128.9 (CH), 129.0 (CH), 133.0 (C_{quat}), 133.3 (C_{quat}), 133.4 (C_{quat}), 134.32 (CH), 134.34 (CH), 134.4 (CH), 134.44 (CH), 136.1 (C_{quat}), 138.8 (C_{quat}), 142.1 (C_{quat}), 144.9 (C_{quat}), 168.2 (C_{quat}),172.7 (C_{quat}), 182.5 (C_{quat}),182.8 (C_{quat}). IR (KBr) $\tilde{\nu}$ [cm^{-1}] = 3296 (w), 2927 (m), 1776 (w), 1736 (w), 1719 (w), 1671 (s), 1629 (s), 1594 (s), 1560 (m), 1509 (m), 1491 (m), 1460 (s), 1364 (m), 1325 (s), 1292 (s), 1249 (s), 1227 (s), 934 (m), 807 (m), 751 (m), 711 (m). UV–vis (CH_2Cl_2) λ_{max} (ε) [nm] = 258 (92100), 326 (18900). MALDI-MS: *m*/*z* = 749.3 ([M]$^+$). Anal. calcd. for $C_{47}H_{47}N_3O_4S$ (749.3): C 75.27, H 6.32, N 5.60; Found: C 75.27, H 6.59, N 5.57.

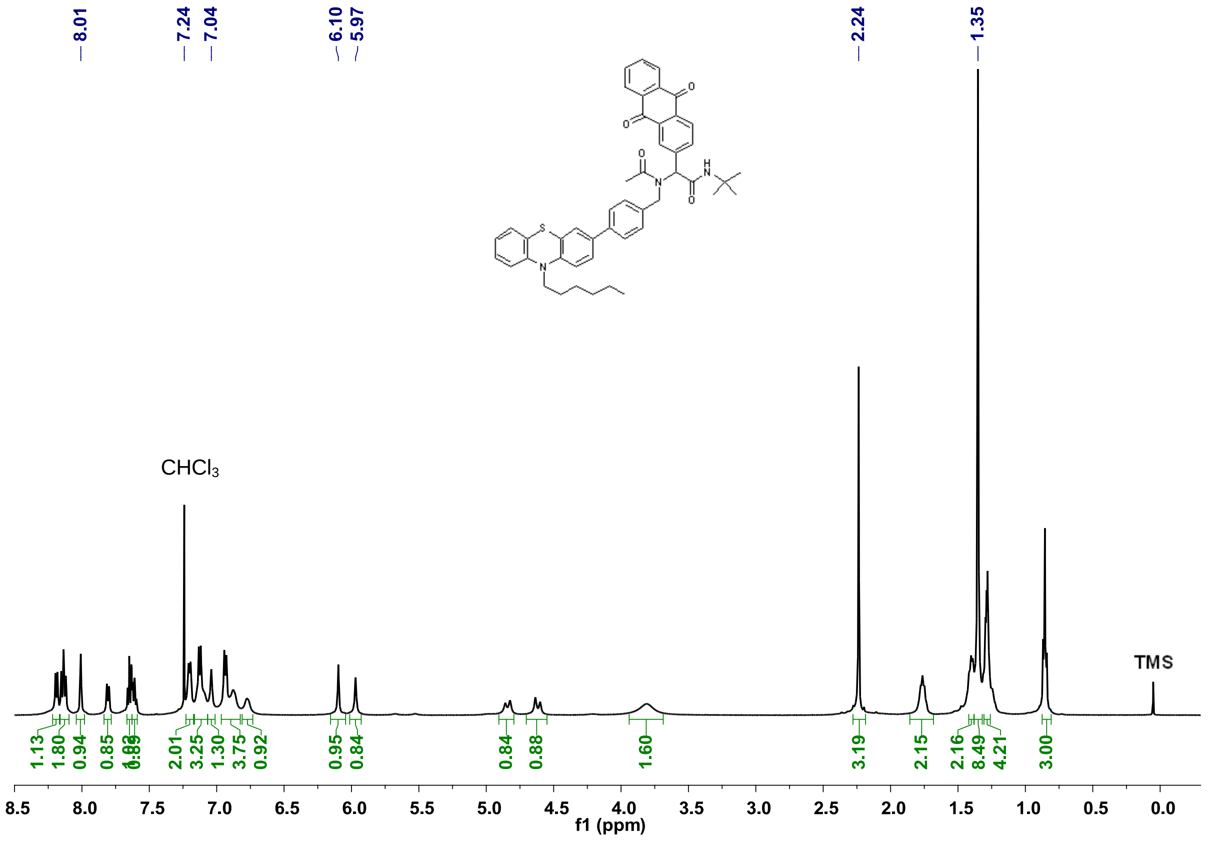

^{1}H NMR of **8c** ($CDCl_3$, 298 K, 500 MHz, δ in ppm).

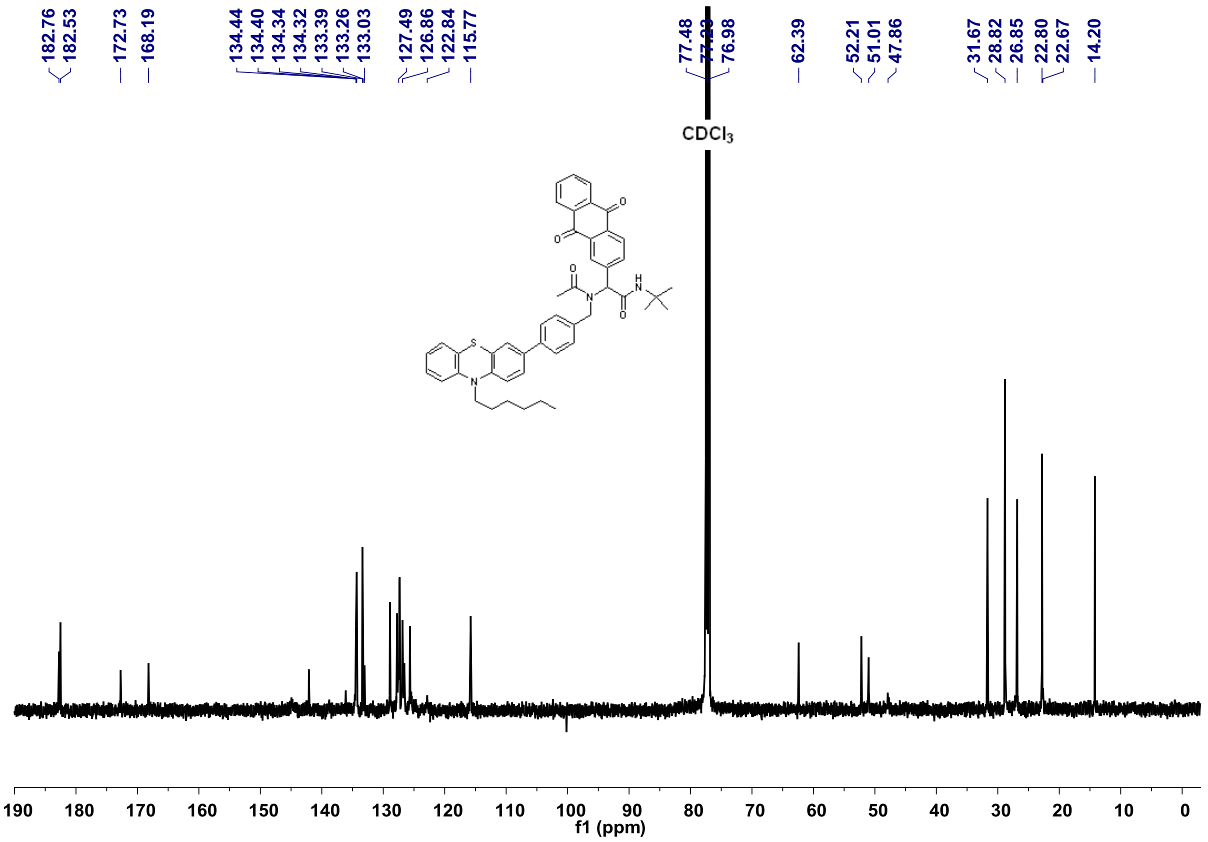

^{13}C NMR of **8c** ($CDCl_3$, 298 K, 126 MHz, δ in ppm).

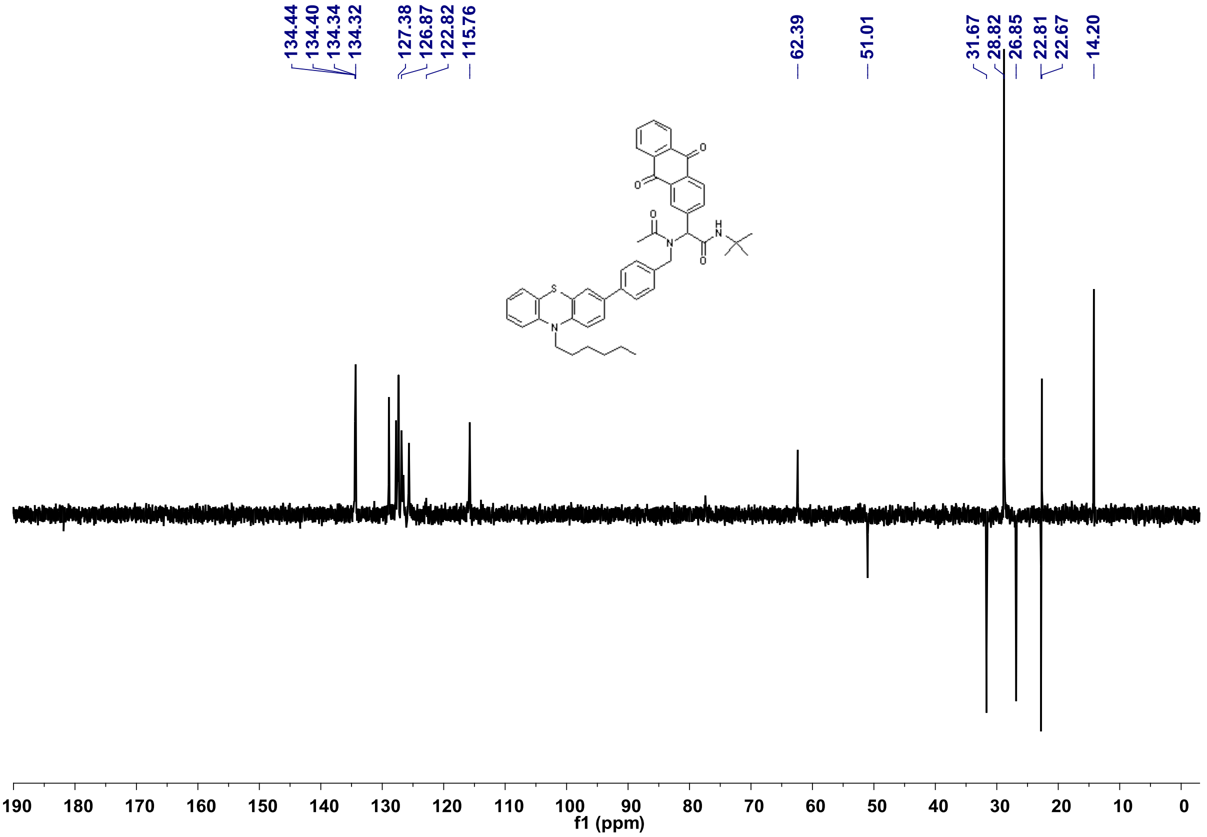

135-DEPT of **8c** ($CDCl_3$, 298 K, 126 MHz, δ in ppm).

3.4 *N*-(*tert*-Butyl)-2-(*N*-((10,10'-dihexyl-10*H*,10'*H*-[3,3'-biphenothiazin]-7-yl)methyl)acetamido)-2-(9,10-dioxo-9,10-dihydroanthracen-2-yl) acetamide (8d)

Purification by column chromatography on silica gel (*n*-hexane/ethyl acetate 2:1) gave 287 mg (60%) of compound **8d** as a brown solid.

Mp 138 °C. R_f (*n*-hexane/ethyl acetate 2:1) = 0.24. ^{1}H NMR (500 MHz, $CDCl_3$): δ = 0.82-0.88 (m, 6 H), 1.20-1.32 (m, 10 H), 1.34 (s, 9 H), 1.38-1.47 (m, 2 H), 1.54-1.63 (m, 2 H), 1.75-1.86 (m, 2 H), 2.22 (s, 3 H), 3.49-3.97 (m, 2 H), 3.76-3.92 (m, 2 H), 4.51 (d, *J* = 17.0 Hz, 1 H), 4.69 (d, *J* = 17.3 Hz, 1 H), 5.89 (s, 1 H), 6.05 (s, 1 H), 6.44 (s, 1 H), 6.49-6.66 (m, 2 H), 6.76 (d, *J* = 7.8 Hz, 1 H), 6.80-6.95 (m, 3 H), 7.01 (s, 1 H), 7.04-7.18 (m, 3 H), 7.19-7.31 (m, 2 H), 7.59 (td, *J* = 1.2 Hz, *J* = 7.4 Hz, 1 H), 7.61-7.65 (m, 1 H), 7.77 (d, *J* = 7.8 Hz, 1 H), 8.03 (s, 1 H), 8.07 (d, *J* = 7.4 Hz, 1 H), 8.11 (d, *J* = 7.5 Hz, 1 H), 8.14 (d, *J* = 8.0 Hz, 1 H). ^{13}C NMR (125.8 MHz, $CDCl_3$): δ = 14.2 (CH_3), 22.7 (CH_3), 22.8 (CH_2), 26.8 (CH_2), 26.9 (CH_2), 28.8 (CH_3), 31.6 (CH_2), 31.7 (CH_2), 47.8 (CH_2), 50.5 (CH_2), 52.2 (C_{quat}), 62.2 (CH), 115.06 (CH), 115.13 (CH), 115.3 (C_{quat}), 115.6 (CH), 115.8 (CH), 122.6 (CH), 124.1 (CH), 124.9 (CH), 125.1 (CH), 125.2 (CH), 125.3 (CH), 125.5 (CH), 127.3 (CH), 127.4 (CH), 127.5 (CH), 127.6 (CH), 127.7 (CH), 128.7 (CH), 131.3 (C_{quat}), 133.1 (C_{quat}), 133.2 (C_{quat}), 133.36 (C_{quat}), 133.39 (C_{quat}), 134.1 (CH), 134.2 (CH), 134.5 (CH), 142.2 (C_{quat}), 168.1 (C_{quat}), 172.5 (C_{quat}), 182.5 (C_{quat}), 182.6 (C_{quat}). IR (KBr) $\tilde{\nu}$ [cm^{-1}] = 2925 (m), 2855 (m), 2346 (w), 1774 (w), 1718 (w), 1676 (s), 1655 (s), 1638 (s), 1594 (s), 1560 (m), 1520 (m), 1509 (m), 1459 (s), 1364 (m), 1325 (m), 1292 (s), 1245 (m), 931 (w), 807 (w), 746 (w), 710 (m). UV–vis (CH_2Cl_2) λ_{max} (ε) [nm] = 259 (144600), 326 (40200). MALDI-MS: *m*/*z* = 954.4 ([M]$^+$). Anal. calcd. for $C_{59}H_{62}N_4O_4S_2$ (954.4): C 74.18, H 6.51, N 5.81; Found: C 74.05, H 6.49, N 5.73.

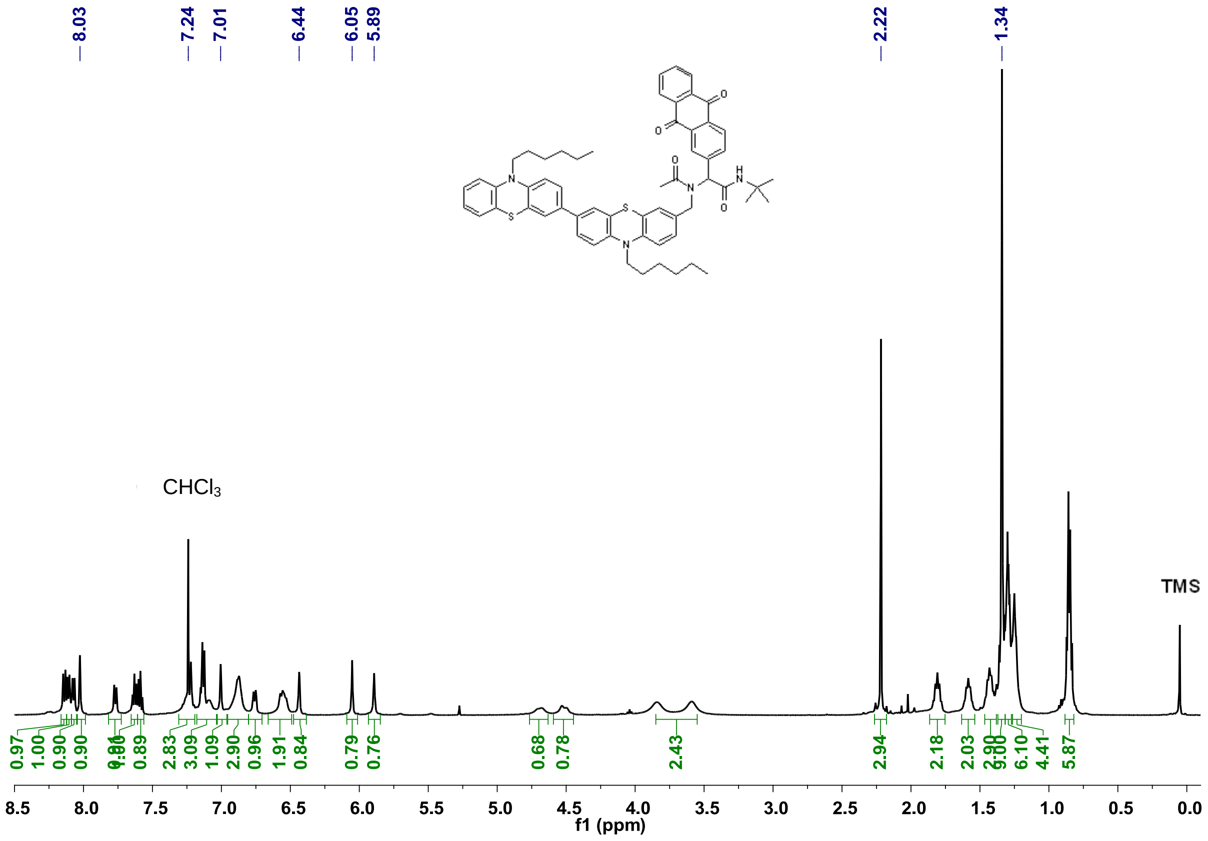

^{1}H NMR of **8d** ($CDCl_3$, 298 K, 500 MHz, δ in ppm).

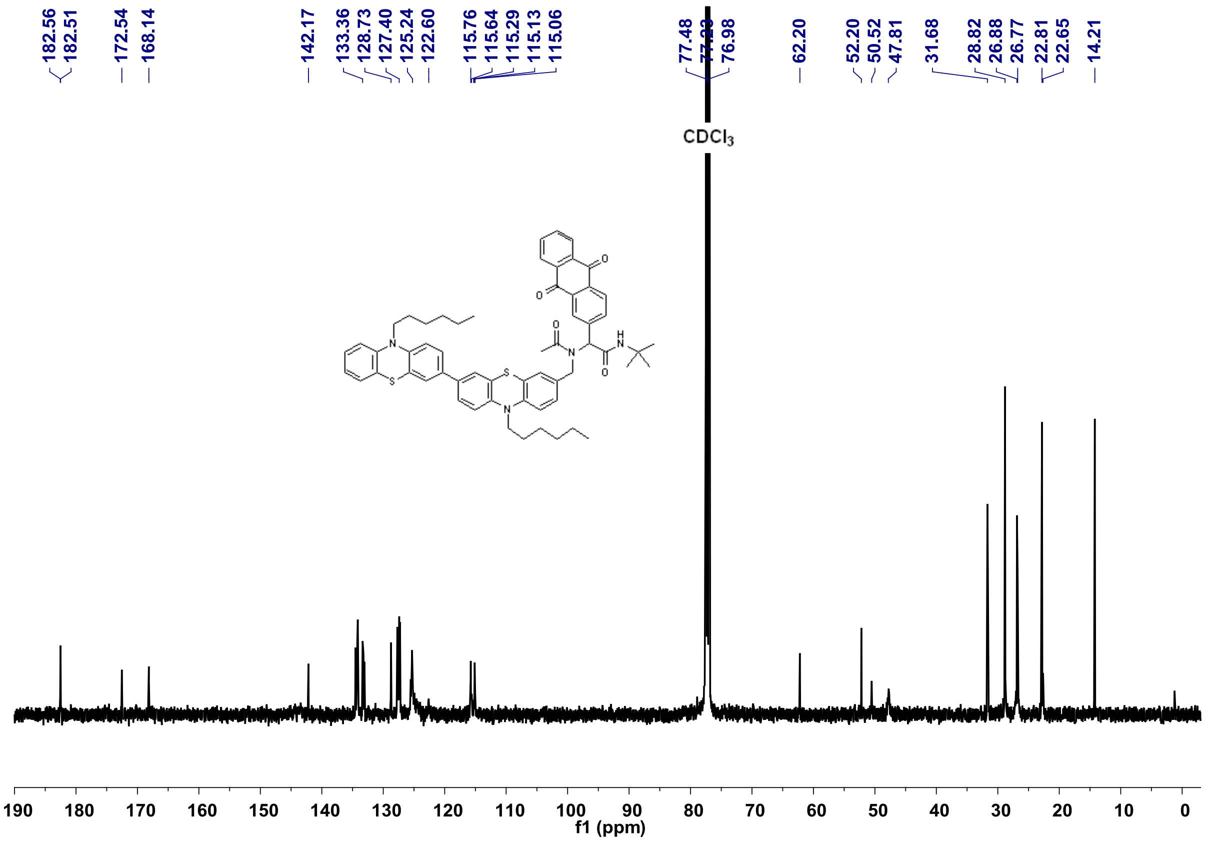

^{13}C NMR of **8d** ($CDCl_3$, 298 K, 126 MHz, δ in ppm).

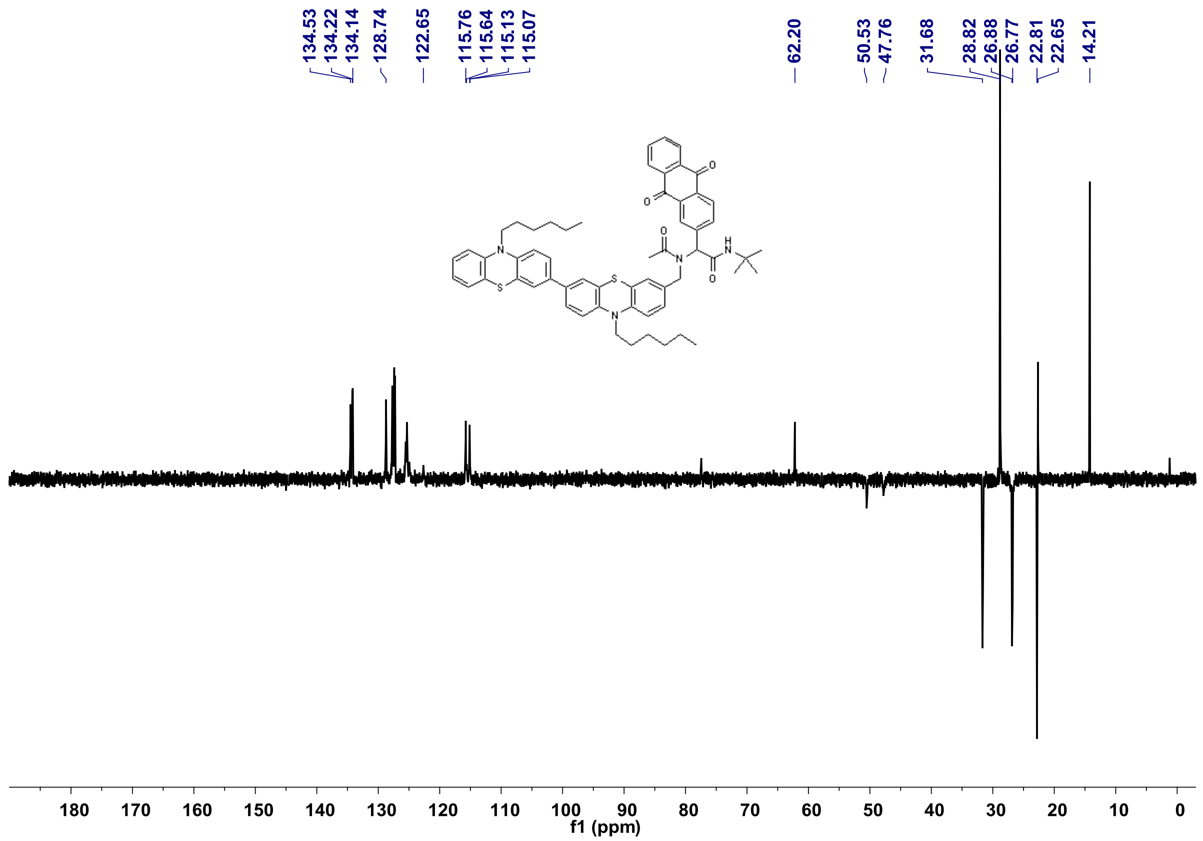

135-DEPT of **8d** ($CDCl_3$, 298 K, 126 MHz, δ in ppm).

3.5 *N*-(*tert*-Butyl)-2-(9,10-dioxo-9,10-dihydroanthracen-2-yl)-2-(*N*-((9-hexyl-9*H*-carbazol-3-yl)methyl)acetamido) acetamide (8e)

Purification by column chromatography on silica gel (*n*-hexane/ethyl acetate 3:1) gave 187 mg (55%) of compound **8e** as an orange solid.

Mp 214 °C. R_f (*n*-hexane/ethyl acetate 2:1) = 0.32. ^{1}H NMR (300 MHz, $CDCl_3$): δ = 0.82 (t, J = 6.6 Hz, 3 H), 1.15-1.28 (m, 6 H), 1.34 (s, 9 H), 1.61-1.73 (m, 2 H), 2.34 (s, 3 H), 3.88-4.10 (m, 2 H), 4.77 (d, J = 16.9 Hz, 1 H), 4.99 (d, J = 16.8 Hz, 1 H), 5.97 (s, 1 H), 6.01 (s, 1 H), 7.00-7.09 (m, 2 H), 7.12 (s, 2 H), 7.22-7.30 (m, 1 H), 7.38 (s, 1 H), 7.61-7.72 (m, 2 H), 7.78 (d, J = 7.9 Hz, 2 H), 7.92 (s, 1 H), 7.96-8.08 (m, 3 H). ^{13}C NMR (75.5 MHz, $CDCl_3$): δ = 14.2 (CH_3), 22.7 (CH_2), 22.9 (CH_3), 27.1 (CH_2), 28.8 (CH_3), 29.0 (CH_2), 31.7 (CH_2), 43.1 (CH_2), 52.1 (CH_2), 62.8 (CH), 108.6 (CH), 109.0 (CH), 118.7 (CH), 119.1 (CH), 120.4 (CH), 122.0 (C_{quat}), 122.8 (C_{quat}), 124.4 (CH), 126.0 (CH), 127.0 (CH), 127.2 (CH), 127.4 (CH), 127.5 (C_{quat}), 128.6 (CH), 132.8 (C_{quat}), 132.9 (C_{quat}), 133.2 (C_{quat}), 133.9 (CH), 134.0 (CH), 134.4 (CH), 139.5 (C_{quat}), 140.6 (C_{quat}), 142.3 (C_{quat}), 168.3 (C_{quat}), 172.6 (C_{quat}), 182.3 (2 C_{quat}). IR (KBr) $\tilde{\nu}$ [cm^{-1}] = 3294 (m), 2961 (m), 1775 (w), 1736 (w), 1719 (w), 1675 (s), 1655 (m), 1619 (s), 1594 (s), 1560 (s), 1509 (w), 1459 (m), 1413 (m), 1333 (s), 1292 (s), 1246 (m), 1224 (m), 1152 (m), 963 (w), 931 (w), 814 (w), 786 (w), 749 (w), 706 (m), 670 (w), 556 (w). UV–vis (CH_2Cl_2) λ_{max} (ε) [nm] = 250 (57600), 265 (69400), 298 (21200), 335 (10500). EI-MS: *m*/*z* = 641 ($[M]^+$, 0.3), 598 ($[M - COCH_3]^+$, 0.7), 321 ($[M - Cz(Hex) - CH_2 - {}^tBu]^+$, 100), 264 ($[Cz(Hex) - CH_2]^+$, 42), 57 ([^{t}Bu], 23), 44 ([$COCH_3$], 65). Anal. calcd. for $C_{41}H_{43}N_3O_4$ (641.3): C 76.73, H 6.75, N 6.55; Found: C 76.54, H 6.55, N 6.47.

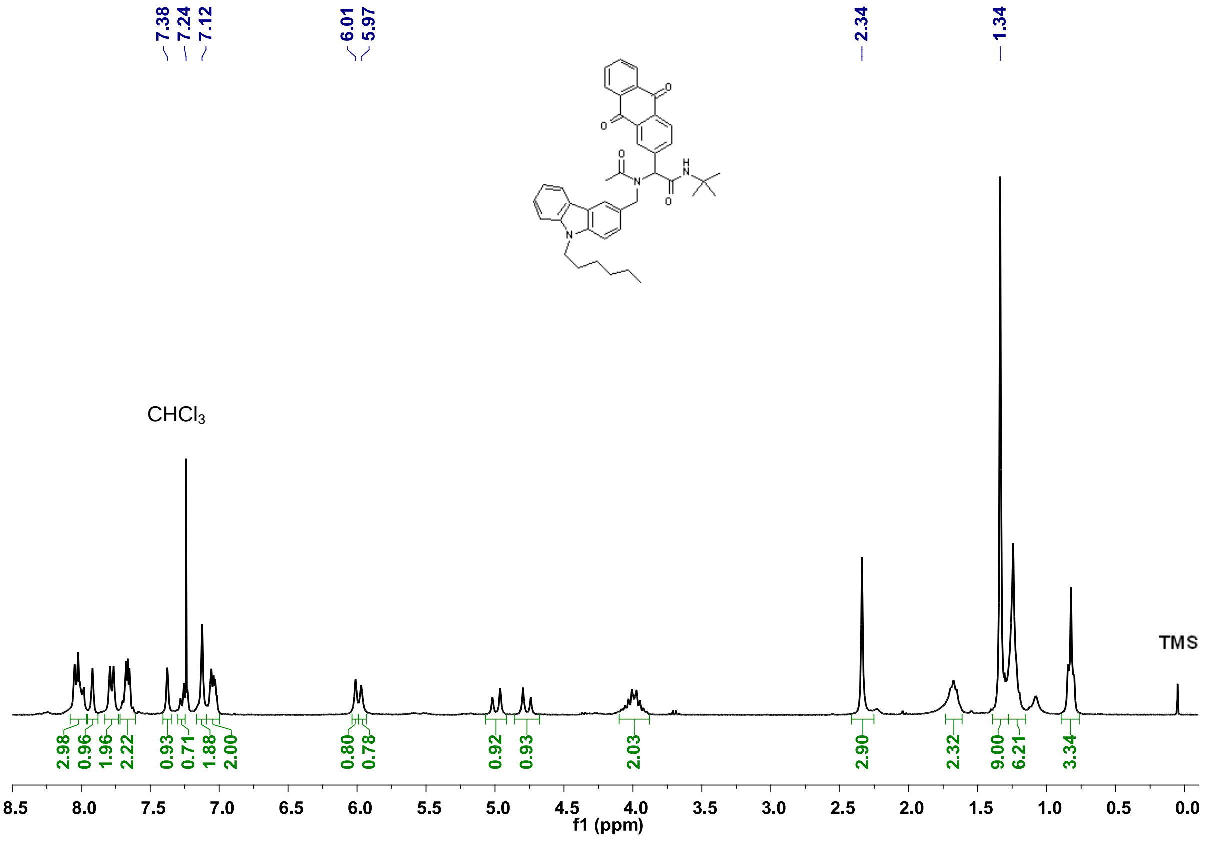

^{1}H NMR of **8e** ($CDCl_3$, 298 K, 300 MHz, δ in ppm).

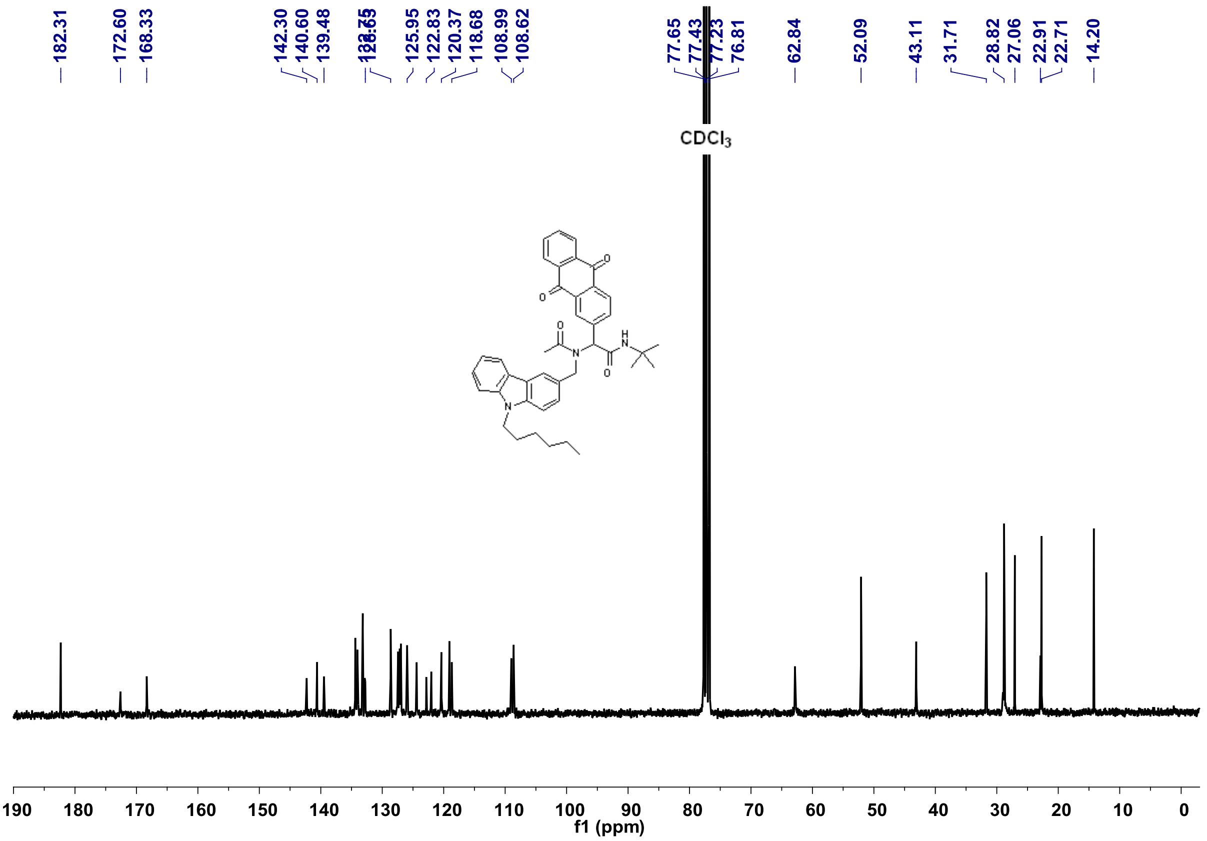

^{13}C NMR of **8e** ($CDCl_3$, 298 K, 76 MHz, δ in ppm).

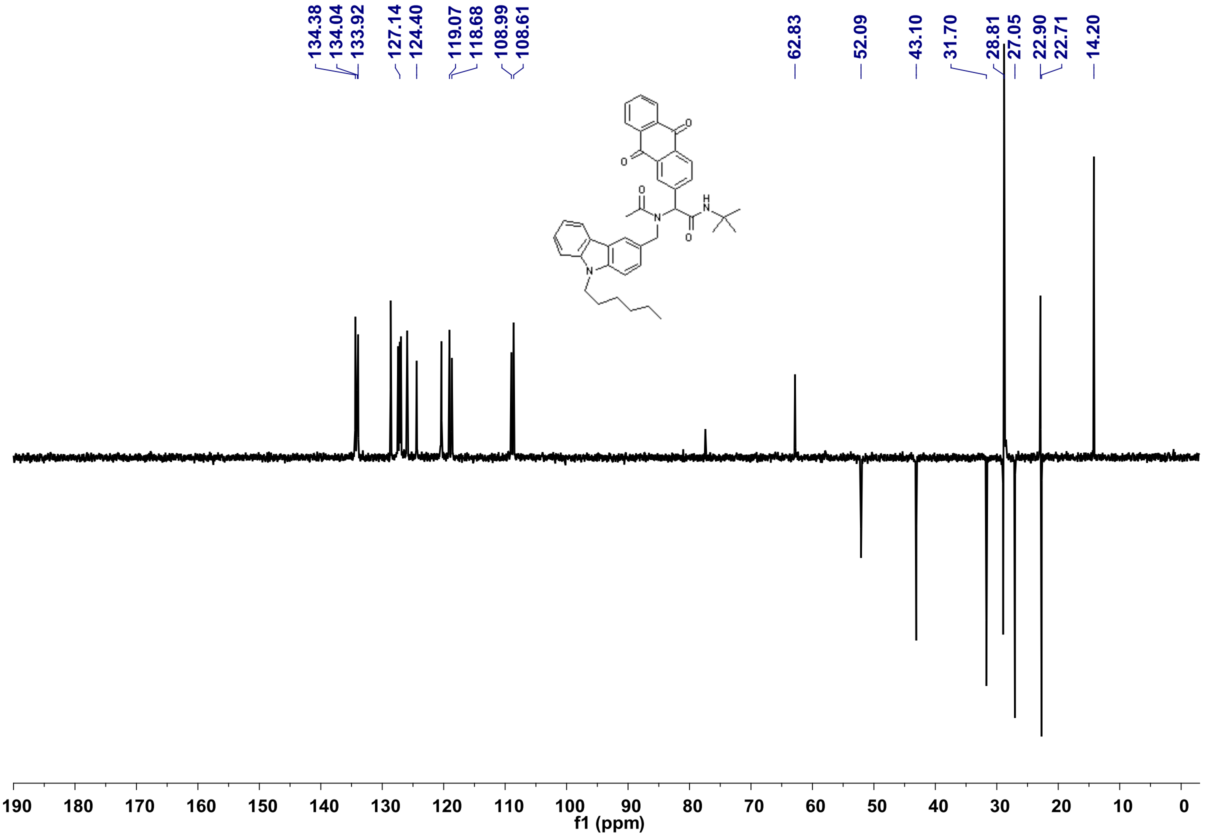

135-DEPT of **8e** ($CDCl_3$, 298 K, 76 MHz, δ in ppm).

3.6 *N*-(*tert*-Butyl)-2-(9,10-dioxo-9,10-dihydroanthracen-2-yl)-2-(*N*-(4-(9-hexyl-9*H*-carbazol-3-yl)benzyl)acetamido) acetamide (8f)

Purification by column chromatography on silica gel (*n*-hexane/ethyl acetate 2:1) gave 162 mg (45%) of compound **8f** as a yellow solid.

Mp 219 °C. R_f (*n*-hexane/ethyl acetate 1:1) = 0.32. ^{1}H NMR (300 MHz, $CDCl_3$): δ = 0.85 (t, J = 7.1 Hz, 3 H), 1.16-1.33 (m, 6 H), 1.37 (s, 9 H), 1.75-1.93 (m, 2 H), 2.27 (s, 3 H), 4.26 (t, J = 7.2 Hz, 2 H), 4.67 (d, J = 17.6 Hz, 1 H), 4.88 (d, J = 17.6 Hz, 1 H), 5.99 (s, 1 H), 6.10 (s, 1 H), 7.04 (d, J = 7.9 Hz, 2 H), 7.21 (d, J = 6.9 Hz, 1 H), 7.30 (d, J = 8.5 Hz, 1 H), 7.38 (d, J = 8.1 Hz, 1 H), 7.40-7.50 (m, 4 H), 7.51-7.64 (m, 2 H), 7.84 (d, J = 7.7 Hz, 1 H), 8.02 (s, 1 H), 8.05 (d, J = 7.8 Hz, 1 H), 8.10 (d, J = 1.5 Hz, 1 H), 8.13 (dd, J = 1.5 Hz, J = 7.5 Hz, 1 H), 8.18 (d, J = 7.7 Hz, 2 H). ^{13}C NMR (75.5 MHz, $CDCl_3$): δ = 14.2 (CH_3), 22.75 (CH_2), 22.77 (CH_3), 27.2 (CH_2), 28.8 (CH_3), 29.2 (CH_2), 31.8 (CH_2), 43.4 (CH_2), 51.2 (CH_2), 52.2 (C_{quat}), 62.7 (CH), 108.96 (CH), 108.98 (CH), 118.7 (CH), 119.1 (CH), 120.7 (CH), 123.1 (C_{quat}), 123.4 (C_{quat}), 124.9 (CH), 126.0 (CH), 126.9 (CH), 127.3 (CH), 127.37 (CH), 127.39 (CH), 127.8 (CH), 128.7 (CH), 131.3 (C_{quat}), 133.1 (C_{quat}), 133.4 (C_{quat}), 133.5 (C_{quat}), 134.18 (CH), 134.23 (CH), 134.6 (CH), 135.3 (C_{quat}), 140.1 (C_{quat}), 141.0 (C_{quat}), 141.2 (C_{quat}), 142.3 (C_{quat}), 142.9 (C_{quat}), 168.2 (C_{quat}), 172.8 (C_{quat}), 182.6 (C_{quat}), 182.8 (C_{quat}). IR (KBr) $\tilde{\nu}$ [cm^{-1}] = 3265 (w), 3063 (w), 2958 (w), 2926 (w), 1672 (s), 1630 (m), 1593 (m), 1557 (w), 1479 (m), 1468 (m) 1450 (m), 1433 (w), 1414 (m), 1404 (m), 1350 (w), 1325 (m), 1310 (m), 1290 (s), 1275 (m), 1246 (m), 1219 (m), 1200 (w), 1173 (w), 1155 (w), 1123 (w), 1069 (w), 1045 (w), 1016 (w), 991 (m), 883 (w), 858 (w), 812 (m), 788 (w), 731 (m), 710 (s), 669 (m), 637 (m), 617 (w). UV–vis (CH_2Cl_2) λ_{max} (ε) [nm] = 255 (84900), 285 (61700). MALDI-MS: m/z = 718.4 ($[M]^+$). Anal. calcd. for $C_{47}H_{47}N_3O_4$ (717.4): C 78.63, H 6.60, N 5.85; Found: C 78.44, H 6.60, N 5.68.

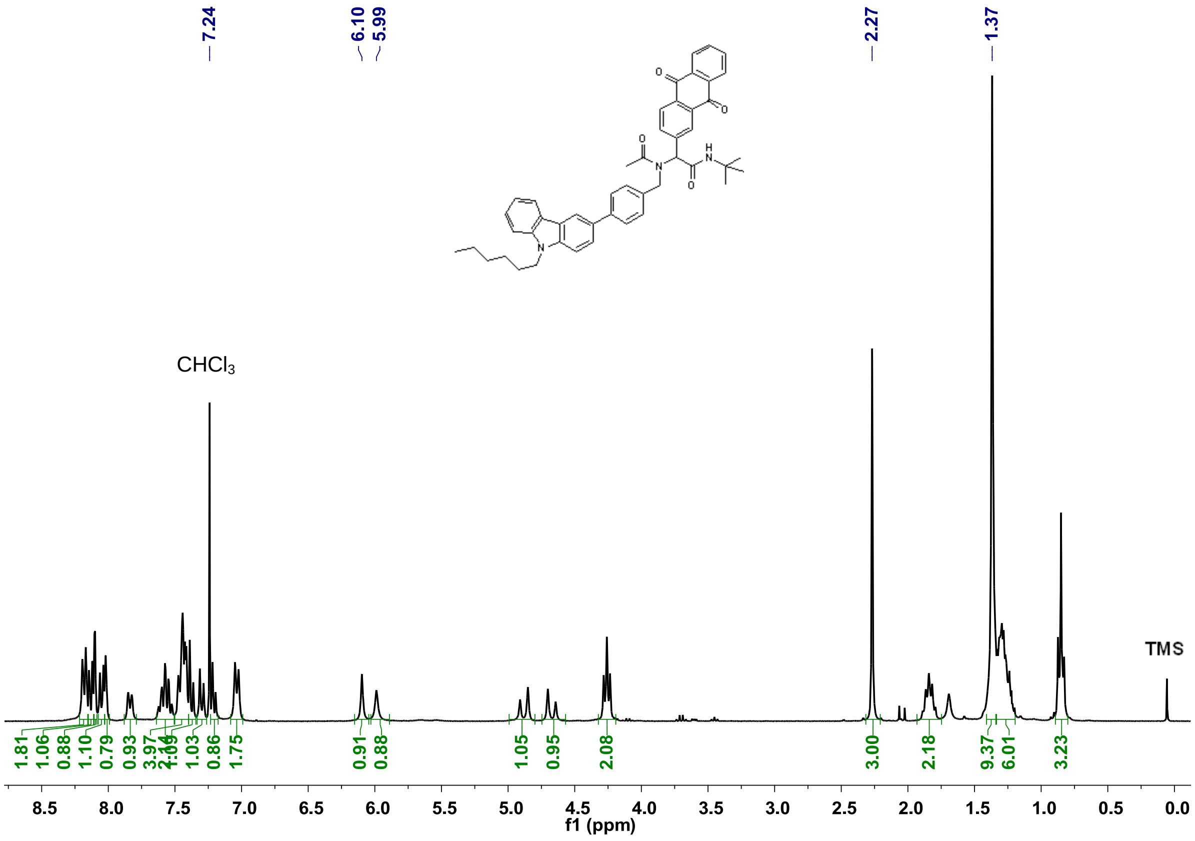

^{1}H NMR of **8f** ($CDCl_3$, 298 K, 300 MHz, δ in ppm).

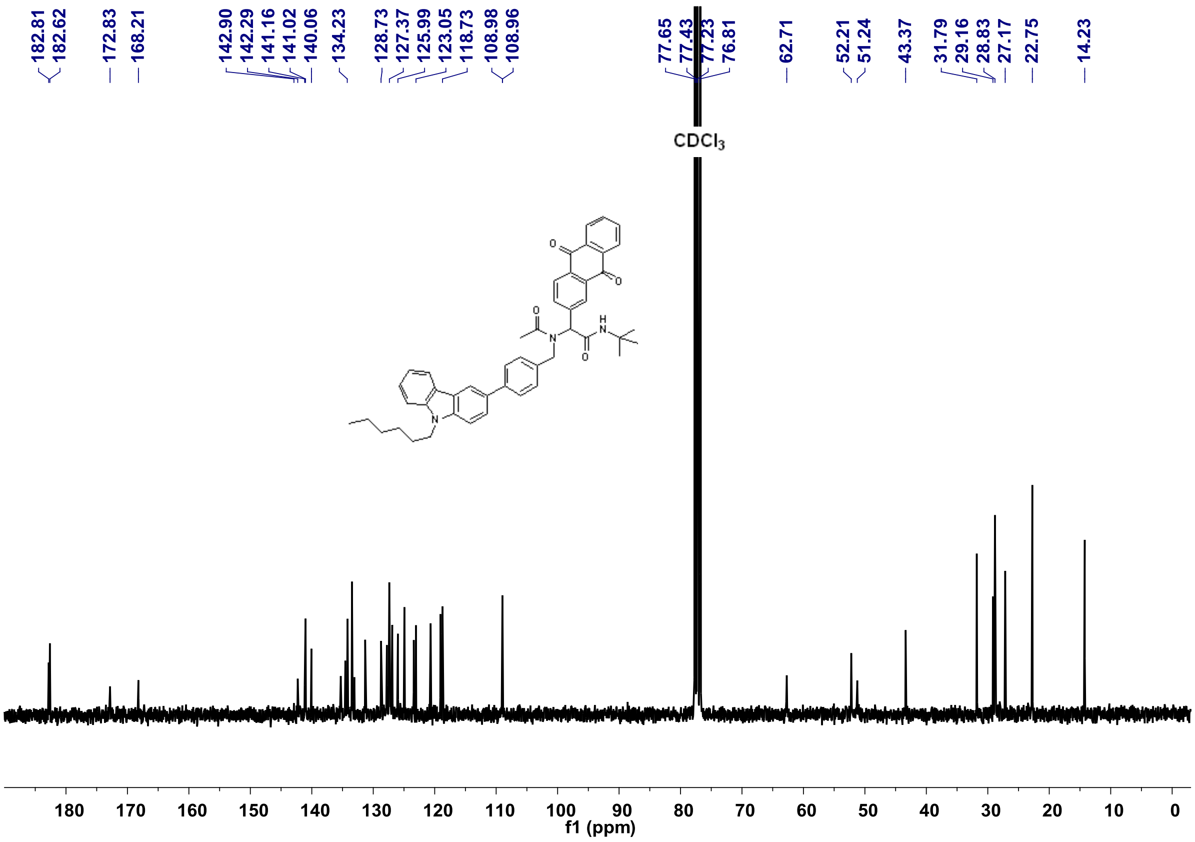

^{13}C NMR of **8f** ($CDCl_3$, 298 K, 76 MHz, δ in ppm).

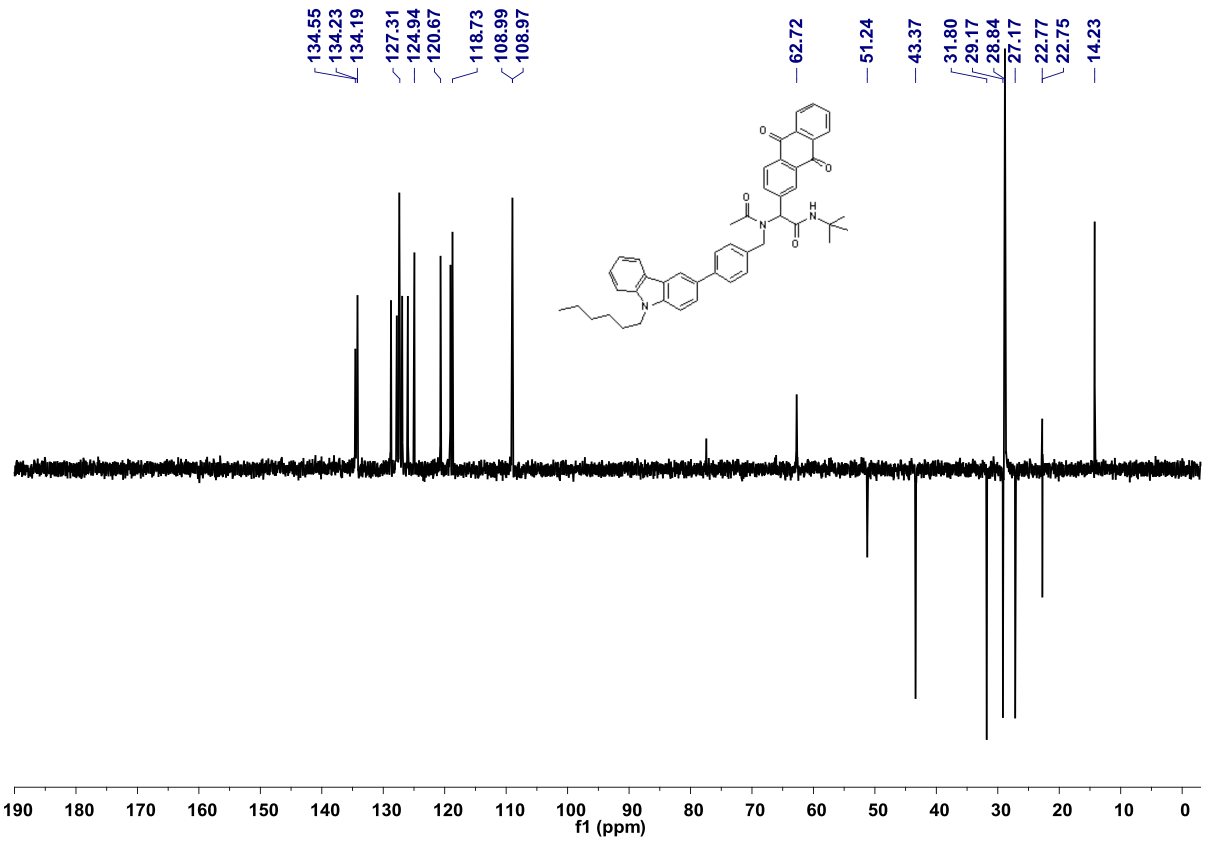

135-DEPT of **8f** ($CDCl_3$, 298 K, 76 MHz, δ in ppm).

3.7 *N*-(*tert*-Butyl)-2-(*N*-(4-(10-hexyl-10*H*-phenothiazin-3-yl)benzyl)-acetamido) propanamide (10a)

Purification by column chromatography on silica gel (*n*-hexane/acetone 6:1) gave 105 mg (35%) of compound **10a** as a yellow waxy solid.

R_f (*n*-hexane/acetone 6:1) = 0.22. ^{1}H NMR (500 MHz, CD_2Cl_2): δ = 0.88 (t, J = 7.1 Hz, 3 H), 1.23 (d, J = 6.9 Hz, 3 H), 1.26 (s, 9 H), 1.30-1.33 (m, 4 H), 1.42-1.48 (m, 2 H), 1.81 (quint, J = 7.6 Hz, 2 H), 2.07 (s, 3 H), 3.87 (t, J = 7.2 Hz, 2 H), 4.60 (q, J = 18.0 Hz, 2 H), 4.97 (q, J = 7.1 Hz, 1H), 6.20 (s, 1 N H), 6.88-6.96 (m, 3 H), 7.13 (dd, J = 1.4 Hz, J = 7.6 Hz, 1 H), 7.14-7.19 (m, 1 H), 7.26 (d, J = 8.0 Hz, 2 H), 7.36 (d, J = 1.8 Hz, 1 H), 7.39 (dd, J = 1.9 Hz, J = 8.4 Hz, 1 H), 7.52 (d, J = 8.1 Hz, 2H). ^{13}C NMR (125.8 MHz, CD_2Cl_2): δ = 14.3 (CH_3), 14.6 (CH_3), 22.7 (CH_3), 23.2 (CH_2), 27.1 (CH_2), 27.3 (CH_2), 28.9 (CH_2), 32.0 (CH_2), 48.0 (CH_2), 48.9 (CH_2), 51.3 (C_{quat}), 54.0 (CH), 116.0 (CH), 116.1 (CH), 122.9 (CH), 124.8 (C_{quat}), 125.7 (C_{quat}), 125.9 (CH), 126.3 (CH), 127.1 (CH), 127.2 (CH), 127.8 (CH), 127.9 (CH), 135.1 (C_{quat}), 137.3 (C_{quat}), 139.4 (C_{quat}),145.2 (C_{quat}),145.6 (C_{quat}),170.8 (C_{quat}), 173.0 (C_{quat}). IR (KBr) $\tilde{\nu}$ [cm^{-1}] = 2978 (m), 2970 (m), 2928 (w), 1680 (m), 1630 (m), 1601 (w), 1576 (w), 1523 (m), 1489 (m), 1460 (s), 1414 (m), 1391 (m), 1362 (m), 1333 (m), 1294 (m), 1252 (m), 1225 (m), 1190 (m), 1177 (m), 1138 (m), 1107 (m), 1078 (w), 1037 (w), 1015 (w), 951 (w), 883 (m), 831 (w), 806 (s), 787 (m), 748 (m), 735 (m), 708 (m), 685 (w), 610 (m). UV–vis (CH_2Cl_2) λ_{max} (ε) [nm] = 269 (61000), 320 (18000). MALDI-MS: m/z = 557.3 ([M]$^+$). Anal. calcd. for $C_{34}H_{43}N_3O_2S \cdot C_4H_8O_2$ (557.3 + 88.08): C 70.66, H 7.96, N 6.51; Found: C 70.53, H 7.98, N 6.53.

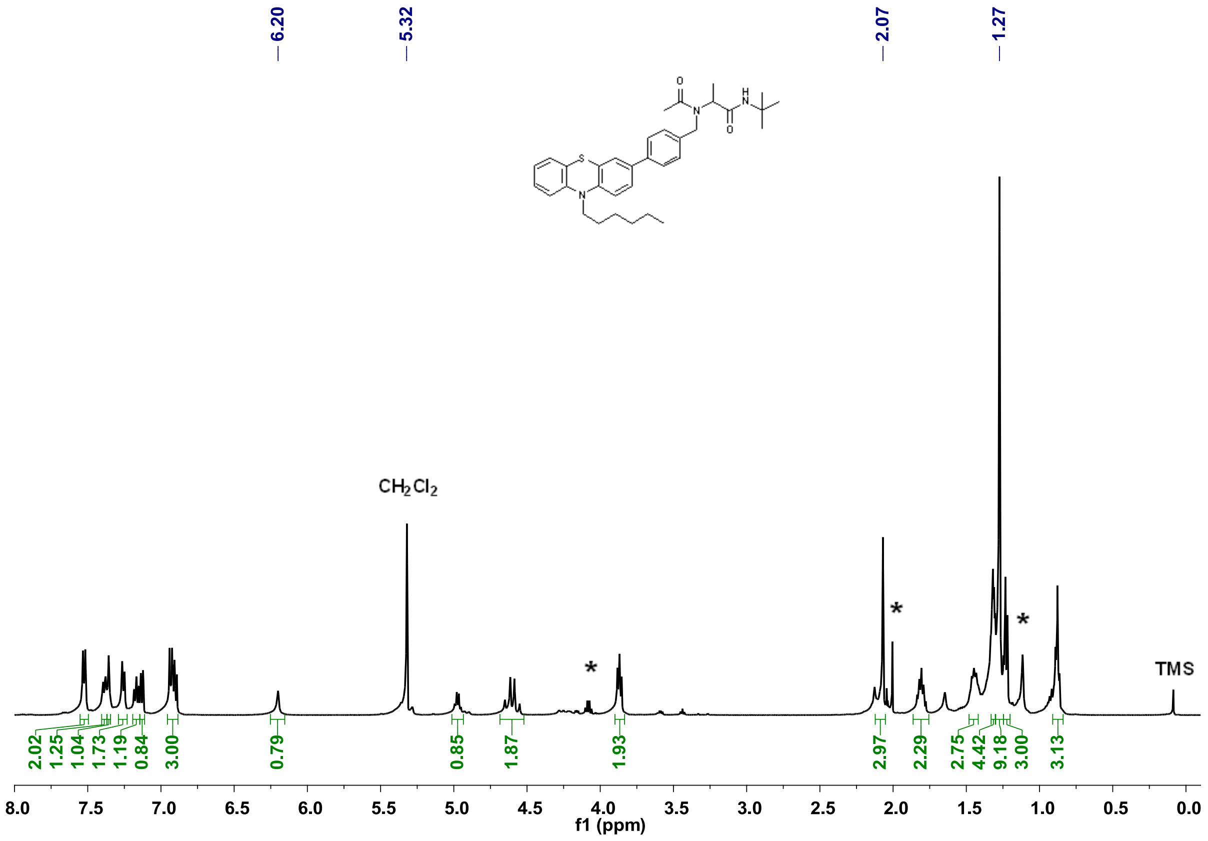

^{1}H NMR of **10a** (CD_2Cl_2, 298 K, 500 MHz, δ in ppm). * Impurities from residual solvents.

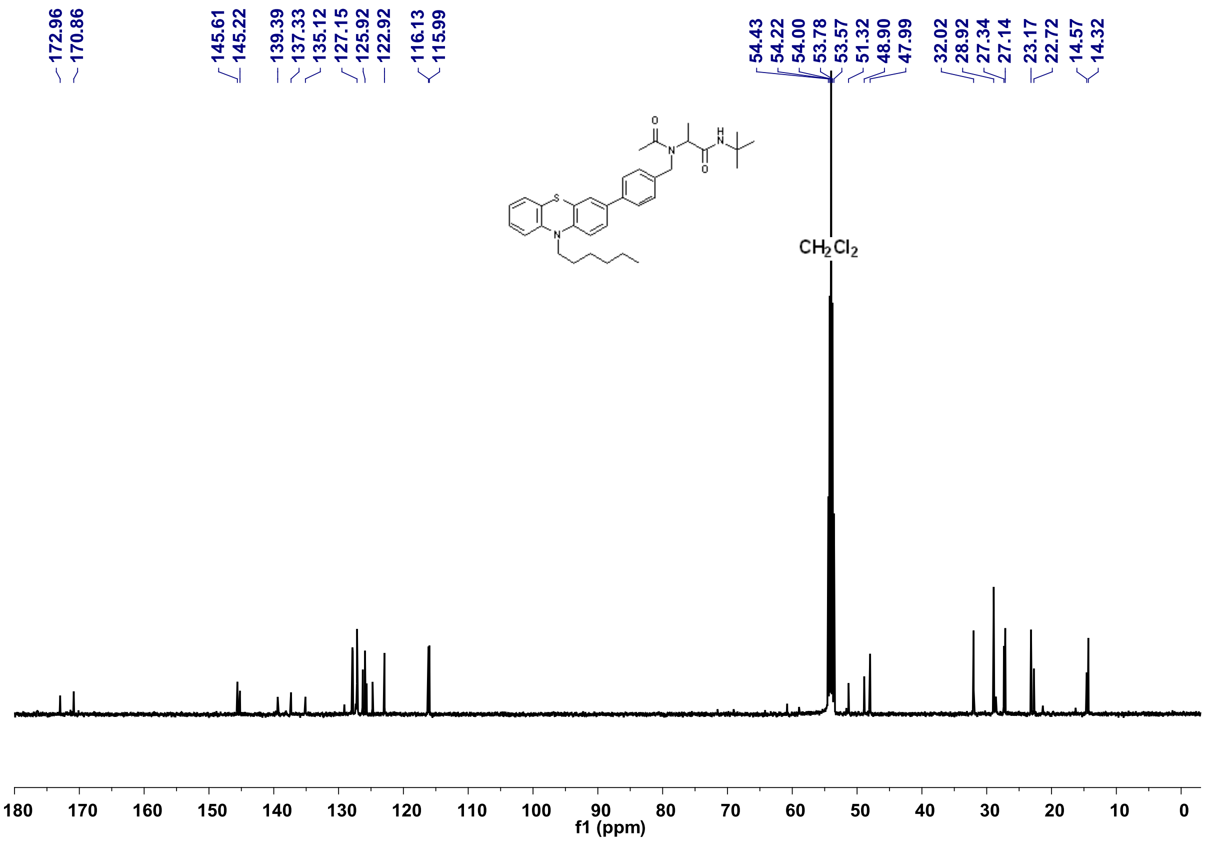

^{13}C NMR of **10a** (CD_2Cl_2, 298 K, 126 MHz, δ in ppm).

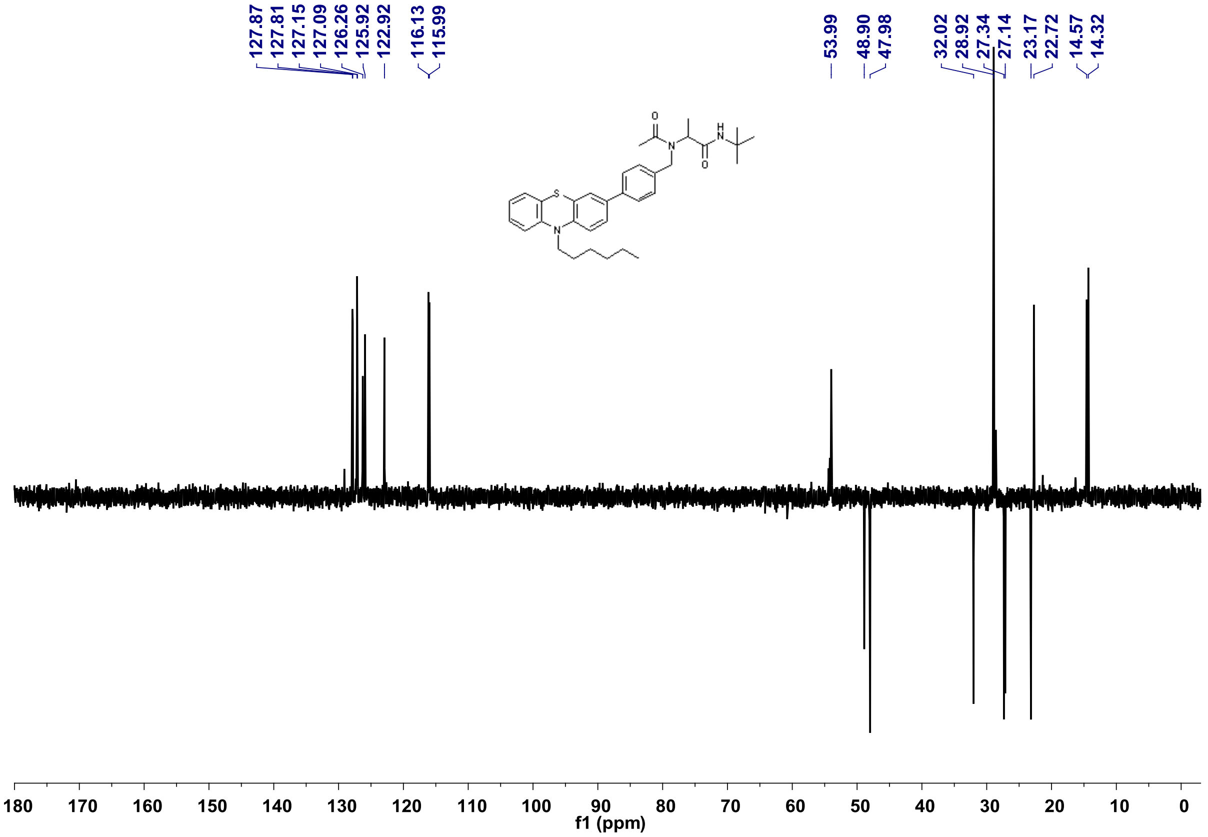

135-DEPT of **10a** (CD_2Cl_2, 298 K, 126 MHz, δ in ppm).

3.8 *N*-(*tert*-Butyl)-2-(*N*-((9-hexyl-9*H*-carbazol-3-yl)methyl)-acetamido) propanamide (10b)

Purification by column chromatography on silica gel (*n*-hexane/ethyl acetate 1:1) gave 162 mg (72%) of compound **10b** as a light yellow resin.

R_f (*n*-hexane/ethyl acetate 1:1) = 0.29. ^{1}H NMR (300 MHz, $CDCl_3$): δ = 0.84 (t, *J* = 7.2 Hz, 3H), 1.25 (d, *J* = 7.1 Hz, 3H), 1.28 (s, 9 H), 1.30-1.43 (m, 4 H), 1.44-1.60 (m, 2 H), 1.83 (quint, *J* = 7.2 Hz, 2 H), 2.16 (s, 3 H), 4.26 (t, *J* = 7.3 Hz, 2 H), 4.76 (s, 2 H), 5.06 (q, *J* = 7.1 Hz, 1 H), 6.25 (s, 1 NH), 7.18-7.24 (m, 1 H), 7.24-7.28 (m, 1 H), 7.32-7.41 (m, 2 H), 7.42-7.49 (m, 1 H), 7.84-7.89 (m, 1 H), 8.05 (d, *J* = 7.8 Hz, 1 H). ^{13}C NMR (75.5 MHz, $CDCl_3$): δ = 14.2 (CH_3), 14.5 (CH_3), 22.68 (CH_2), 22.70 (CH_3), 27.2 (CH_2), 28.9 (CH_2), 29.1 (CH_2), 31.8 (CH_2), 43.4 (CH_2), 49.3 (CH_2), 51.2 (C_{quat}), 53.9 (CH), 109.0 (CH), 109.2 (CH), 117.8 (CH), 119.1 (CH), 120.6 (CH), 122.5 (C_{quat}), 123.3 (C_{quat}), 123.9 (CH), 126.1 (CH), 128.1 (C_{quat}), 139.9 (C_{quat}), 141.0 (C_{quat}), 170.9 (C_{quat}), 173.2 (C_{quat}). IR $\tilde{\nu}$ [cm^{-1}] = 3309 (w), 3273 (w), 3049 (w), 2965 (w), 2926 (w), 2860 (w), 1674 (m), 1622 (s), 1553 (m), 1493 (m), 1472 (m), 1456 (m), 1418 (m), 1389 (w), 1360 (m), 1329 (m), 1258 (m), 1225 (m), 1209 (m), 1188 (m), 1152 (w), 1125 (w), 1090 (w), 1063 (w), 1038 (w), 1011 (w), 976 (w), 891 (w), 804 (m), 770 (w), 745 (s), 723 (m), 648 (w), 617 (w). UV–vis (CH_2Cl_2) λ_{max} (ε) [nm] = 240 (47000), 267 (29000), 298 (17000), 336 (4000), 352 (4000). MALDI-MS: *m*/*z* = 450.0 ([M]$^+$). Anal. calcd. for $C_{28}H_{39}N_3O_2 \cdot 0.25$ HCl (449.3 + 9.11): C 73.85, H 8.76, N 8.91; Found: C 74.09, H 8.60, N 8.91.

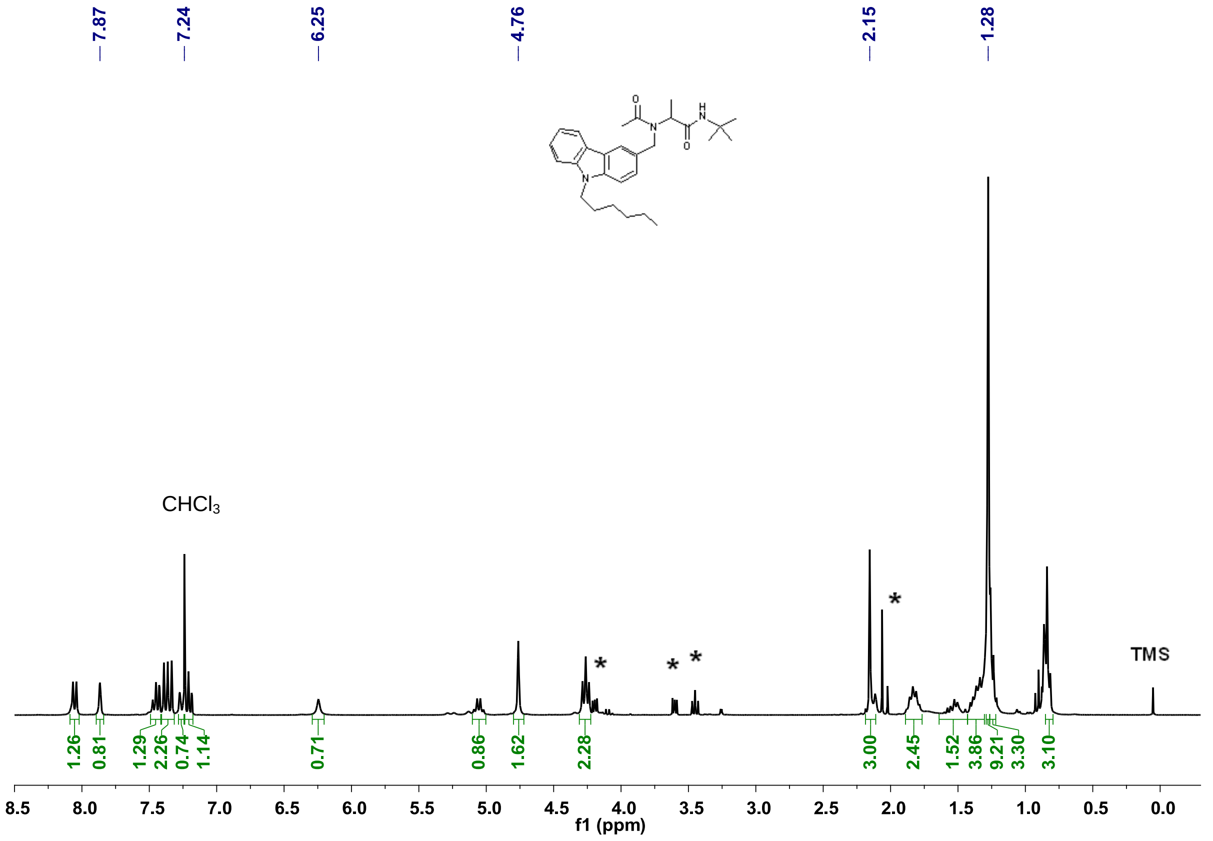

^{1}H NMR of **10b** ($CDCl_3$, 298 K, 300 MHz, δ in ppm). * Impurities from residual solvents.

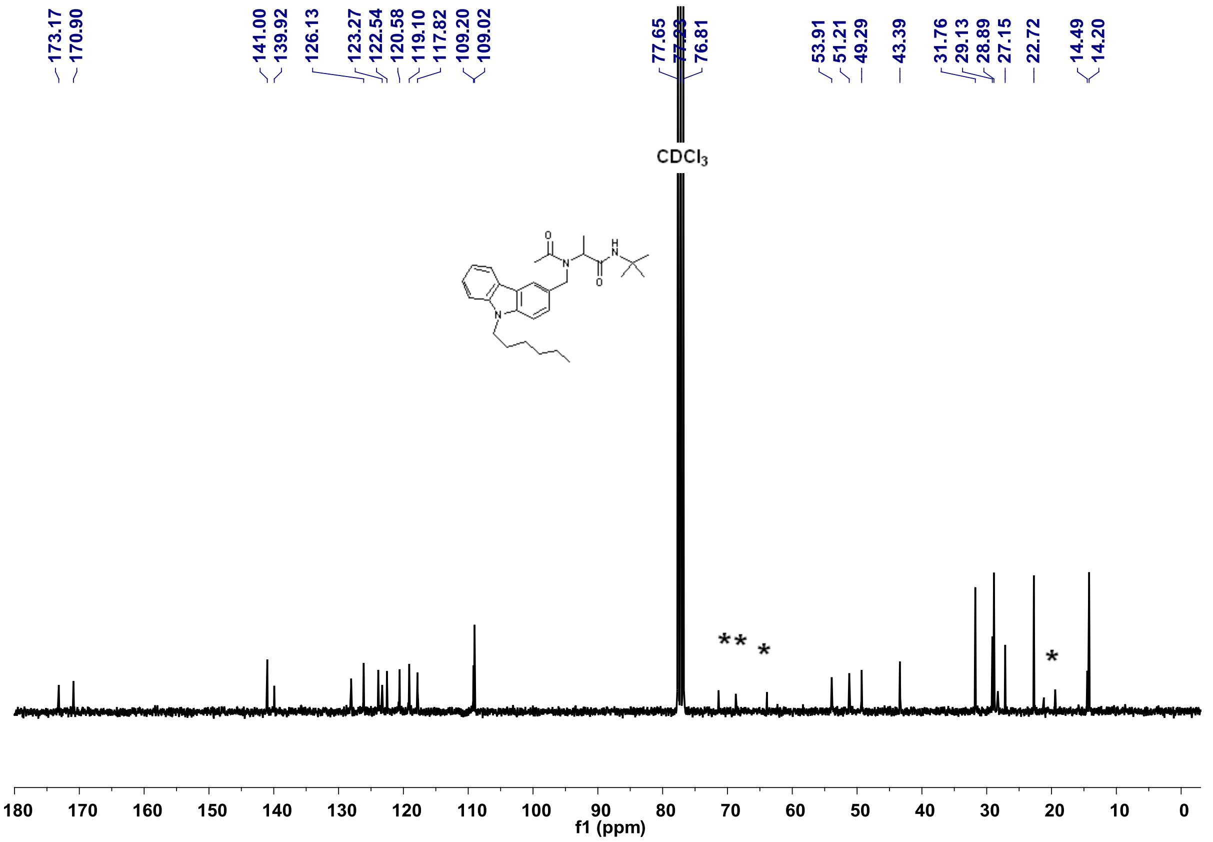

^{13}C NMR of **10b** ($CDCl_3$, 298 K, 76 MHz, δ in ppm). * Impurities from residual solvents.

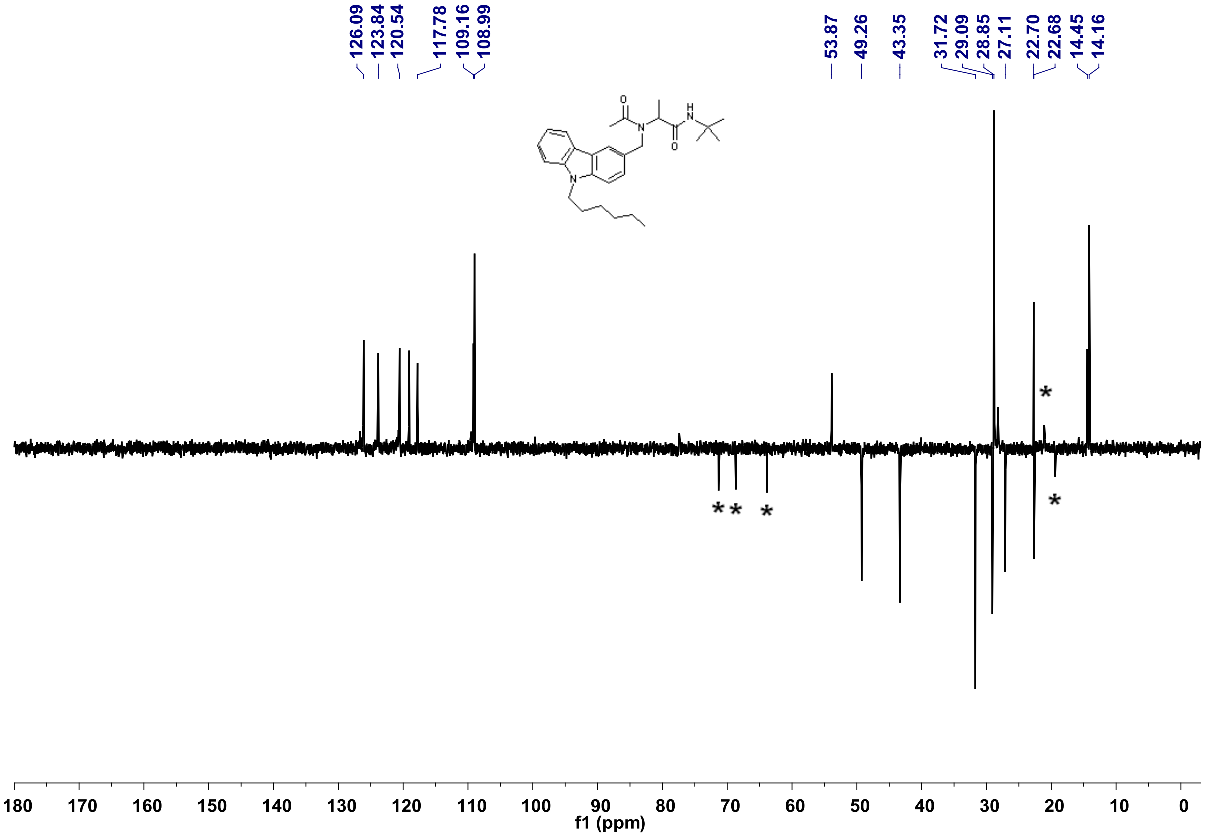

135-DEPT of **10b** ($CDCl_3$, 298 K, 76 MHz, δ in ppm).

4 Cyclic voltammetry of Ugi compounds 8 and 10

4.1 2-(*N*-(3-(10*H*-Phenothiazin-10-yl)propyl)acetamido)-*N*-(*tert*-butyl)-2-(9,10-dioxo-9,10-dihydroanthracen-2-yl) acetamide (8a)

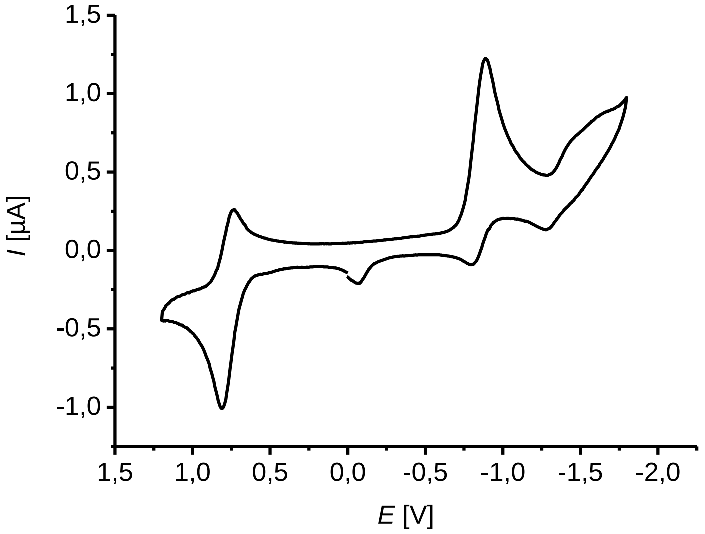

Cyclic voltammogram of dyad **8a** recorded in CH_2Cl_2, T = 298 K, c = 0.1 $molL^{-1}$, Pt working electrode, Pt counter electrode, Ag/AgCl reference electrode, electrolyte $N(n\text{-}Bu)_4PF_6$, scan rate of 100 mVs^{-1}.

4.2 2-(*N*-(4-((10*H*-Phenothiazin-10-yl)methyl)benzyl)acetamido)-*N*-(*tert*-butyl)-2-(9,10-dioxo-9,10-dihydroanthracen-2-yl) acetamide (8b)

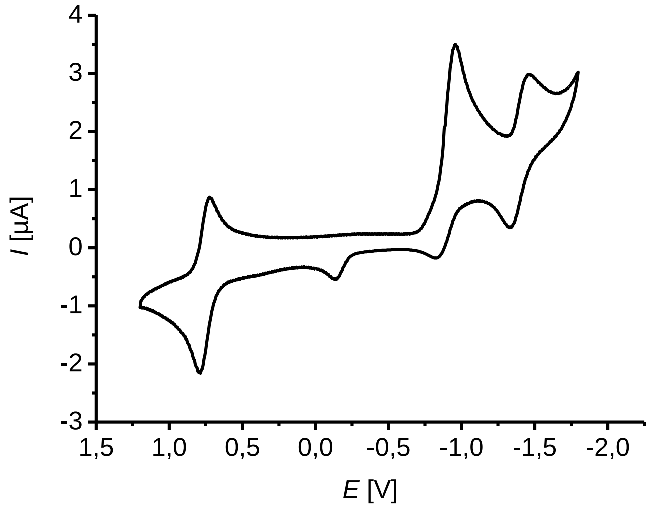

Cyclic voltammogram of dyad **8b** recorded in CH_2Cl_2, T = 298 K, c = 0.1 $molL^{-1}$, Pt working electrode, Pt counter electrode, Ag/AgCl reference electrode, electrolyte $N(n\text{-}Bu)_4PF_6$, scan rate of 250 mVs^{-1}.

4.3 *N*-(*tert*-Butyl)-2-(9,10-dioxo-9,10-dihydroanthracen-2-yl)-2-(*N*-(4-(10-hexyl-10*H*-phenothiazin-3-yl)benzyl)acetamido) acetamide (8c)

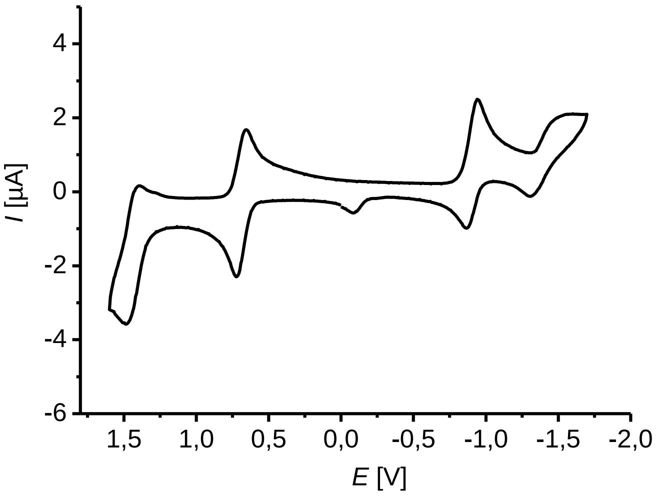

Cyclic voltammogram of dyad **8c** recorded in CH_2Cl_2, *T* = 298 K, *c* = 0.1 $molL^{-1}$, Pt working electrode, Pt counter electrode, Ag/AgCl reference electrode, electrolyte N(*n*-Bu)$_4$PF$_6$, scan rate of 250 mVs^{-1}.

4.4 *N*-(*tert*-Butyl)-2-(*N*-((10,10'-dihexyl-10*H*,10'*H*-[3,3'-biphenothiazin]-7-yl)methyl)acetamido)-2-(9,10-dioxo-9,10-dihydroanthracen-2-yl) acetamide (8d)

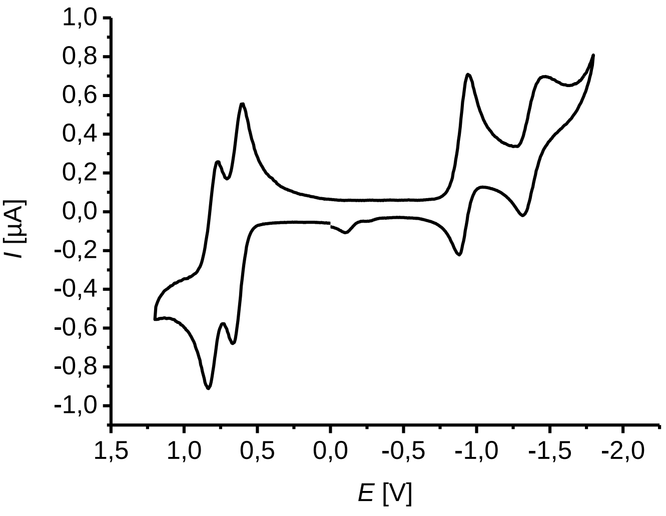

Cyclic voltammogram of dyad **8d** recorded in CH_2Cl_2, *T* = 298 K, *c* = 0.1 $molL^{-1}$, Pt working electrode, Pt counter electrode, Ag/AgCl reference electrode, electrolyte N(*n*-Bu)$_4$PF$_6$, scan rate of 250 mVs^{-1}.

4.5 *N*-(*tert*-Butyl)-2-(9,10-dioxo-9,10-dihydroanthracen-2-yl)-2-(*N*-((9-hexyl-9*H*-carbazol-3-yl)methyl)acetamido) acetamide (8e)

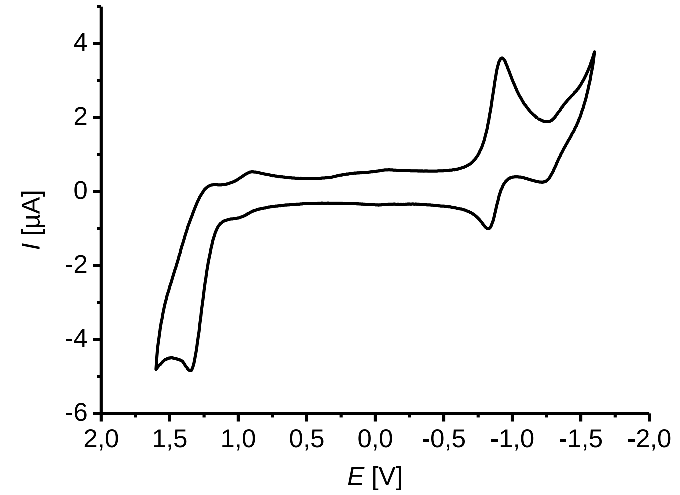

Cyclic voltammogram of dyad **8e** recorded in CH_2Cl_2, T = 298 K, c = 0.1 $molL^{-1}$, Pt working electrode, Pt counter electrode, Ag/AgCl reference electrode, electrolyte $N(n\text{-}Bu)_4PF_6$, scan rate of 100 mVs^{-1}.

4.6 *N*-(*tert*-Butyl)-2-(9,10-dioxo-9,10-dihydroanthracen-2-yl)-2-(*N*-(4-(9-hexyl-9*H*-carbazol-3-yl)benzyl)acetamido) acetamide (8f)

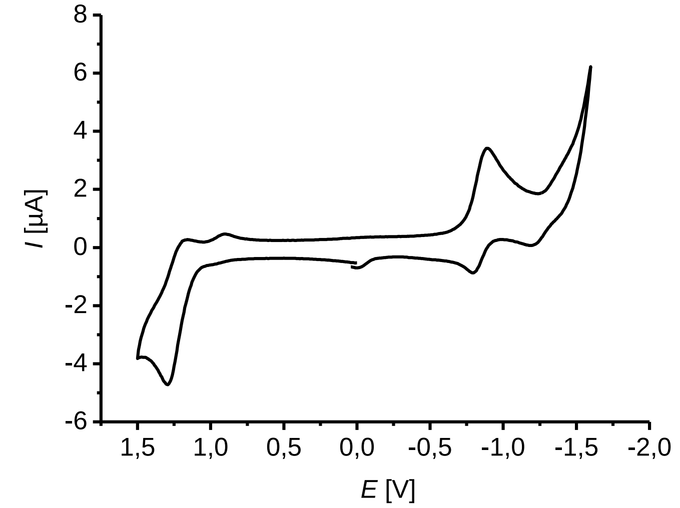

Cyclic voltammogram of dyad **8f** recorded in CH_2Cl_2, T = 298 K, c = 0.1 $molL^{-1}$, Pt working electrode, Pt counter electrode, Ag/AgCl reference electrode, electrolyte $N(n\text{-}Bu)_4PF_6$, scan rate of 100 mVs^{-1}.

4.7 ***N*-(*tert*-Butyl)-2-(*N*-(4-(10-hexyl-10*H*-phenothiazin-3-yl)benzyl)-acetamido) propanamide (10a)**

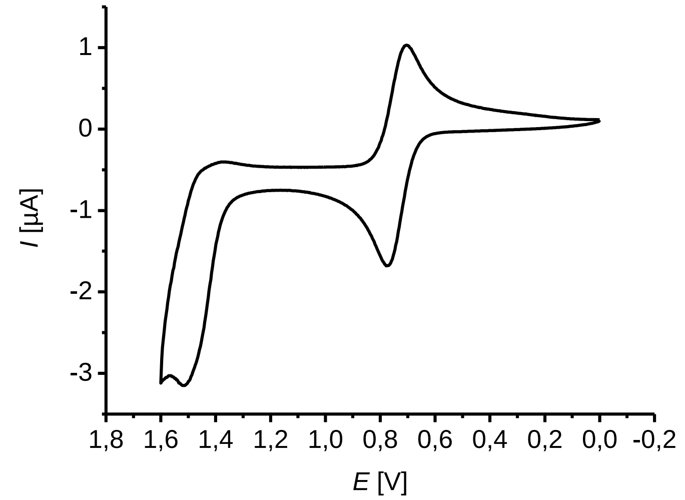

Cyclic voltammogram of dyad **10a** recorded in CH_2Cl_2, T = 298 K, c = 0.1 $molL^{-1}$, Pt working electrode, Pt counter electrode, Ag/AgCl reference electrode, electrolyte N(*n*-Bu)$_4$PF$_6$, scan rate of 100 mVs^{-1}.

4.8 ***N*-(*tert*-Butyl)-2-(*N*-((9-hexyl-9*H*-carbazol-3-yl)methyl)acetamido) propanamide (10b)**

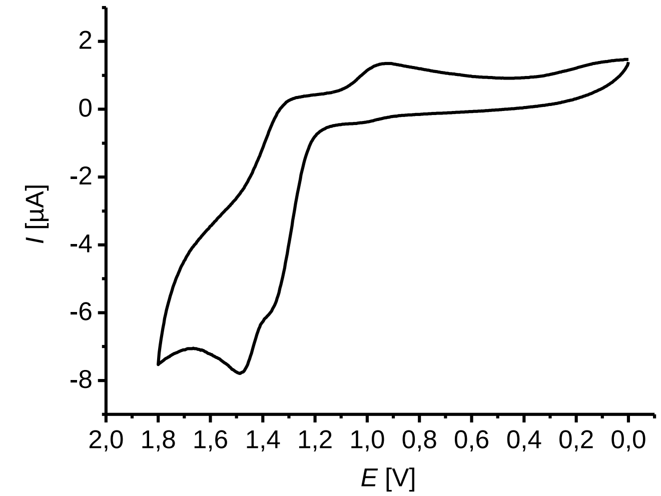

Cyclic voltammogram of dyad **10b** recorded in CH_2Cl_2, T = 298 K, c = 0.1 $molL^{-1}$, Pt working electrode, Pt counter electrode, Ag/AgCl reference electrode, electrolyte N(*n*-Bu)$_4$PF$_6$, scan rate of 100 mVs^{-1}.

5 Absorption spectroscopy of compounds 8 and 10

5.1 2-(*N*-(3-(10*H*-Phenothiazin-10-yl)propyl)acetamido)-*N*-(*tert*-butyl)-2-(9,10-dioxo-9,10-dihydroanthracen-2-yl) acetamide (8a)

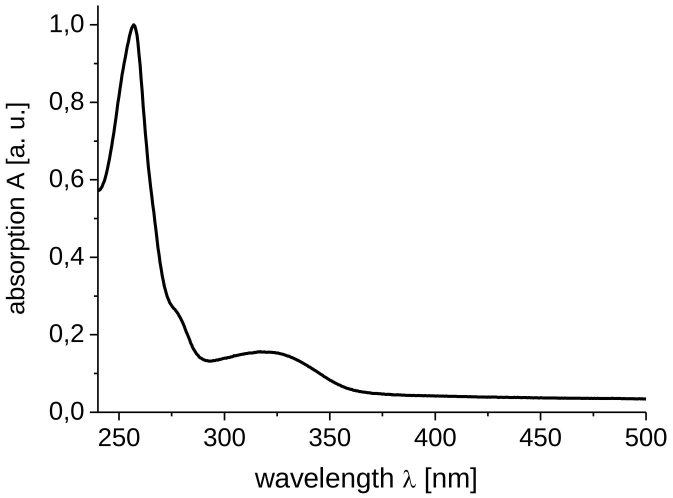

Normalized UV–vis spectrum of **8a** recorded in CH_2Cl_2, T = 298 K.

5.2 2-(*N*-(4-((10*H*-Phenothiazin-10-yl)methyl)benzyl)acetamido)-*N*-(*tert*-butyl)-2-(9,10-dioxo-9,10-dihydroanthracen-2-yl) acetamide (8b)

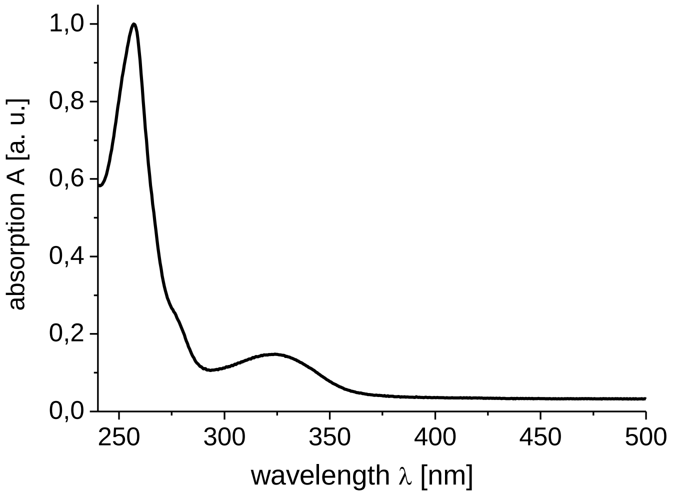

Normalized UV–vis spectrum of **8b** recorded in CH_2Cl_2, T = 298 K.

5.3 *N*-(*tert*-Butyl)-2-(9,10-dioxo-9,10-dihydroanthracen-2-yl)-2-(*N*-(4-(10-hexyl-10*H*-phenothiazin-3-yl)benzyl)acetamido) acetamide (8c)

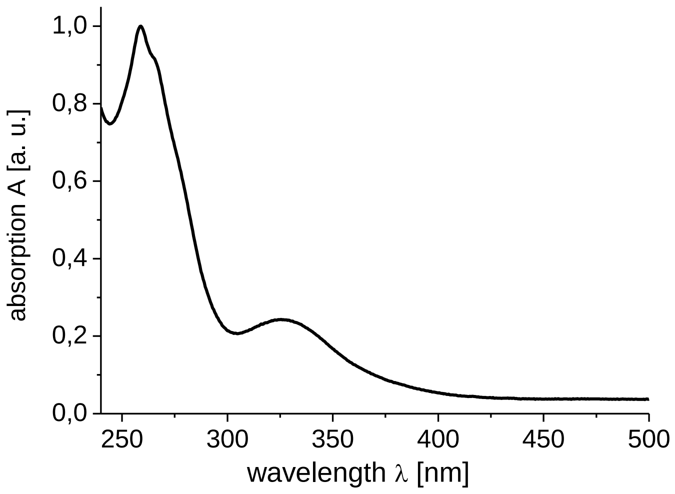

Normalized UV–vis spectrum of **8c** recorded in CH_2Cl_2, T = 298 K.

5.4 *N*-(*tert*-Butyl)-2-(*N*-((10,10'-dihexyl-10*H*,10'*H*-[3,3'-biphenothiazin]-7-yl)methyl)acetamido)-2-(9,10-dioxo-9,10-dihydroanthracen-2-yl) acetamide (8d)

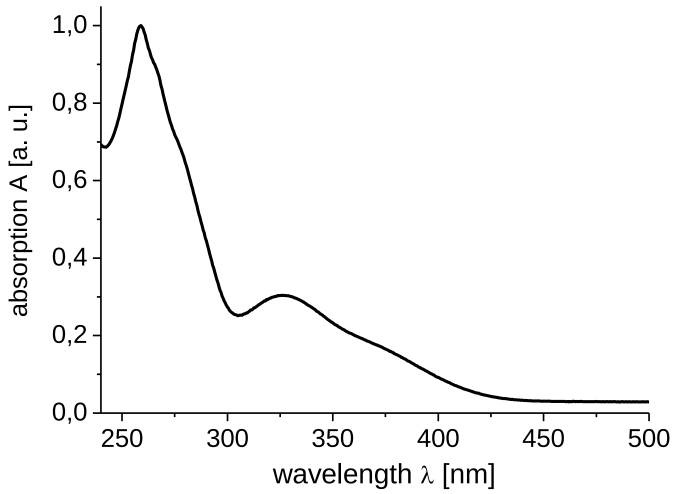

Normalized UV–vis spectrum of **8d** recorded in CH_2Cl_2, T = 298 K.

5.5 *N*-(*tert*-Butyl)-2-(9,10-dioxo-9,10-dihydroanthracen-2-yl)-2-(*N*-((9-hexyl-9*H*-carbazol-3-yl)methyl)acetamido) acetamide (8e)

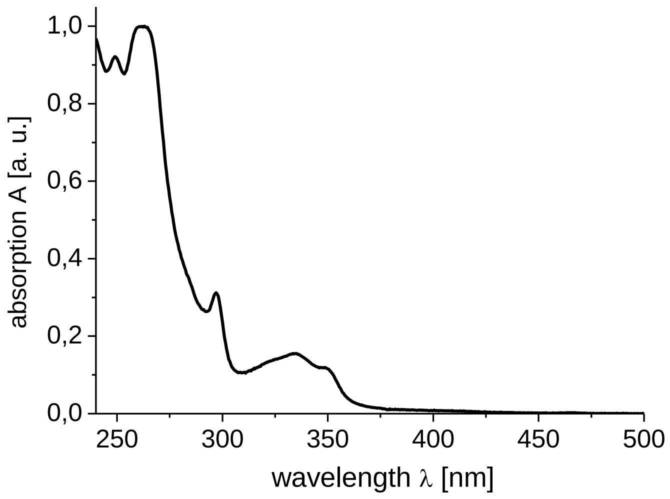

Normalized UV–vis spectrum of **8e** recorded in CH_2Cl_2, T = 298 K.

5.6 *N*-(*tert*-Butyl)-2-(9,10-dioxo-9,10-dihydroanthracen-2-yl)-2-(*N*-(4-(9-hexyl-9*H*-carbazol-3-yl)benzyl)acetamido) acetamide (8f)

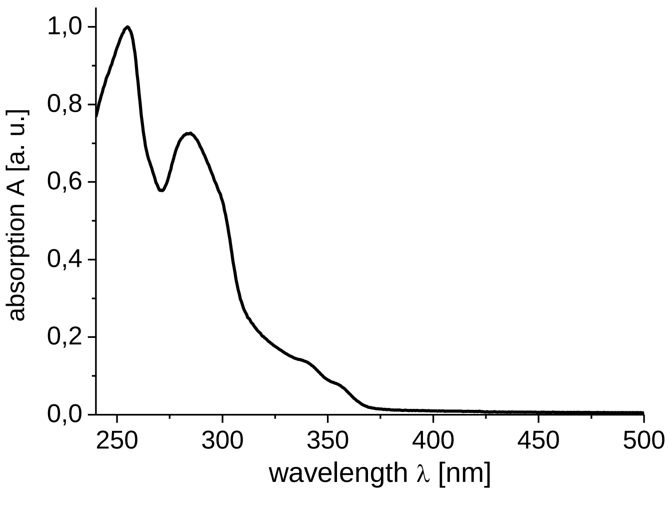

Normalized UV–vis spectrum of **8f** recorded in CH_2Cl_2, T = 298 K.

6 Emission Spectroscopy of 2, 10 and 8

6.1 *N*-(*tert*-Butyl)-2-(*N*-((10-hexyl-10*H*-phenothiazin-3-yl)methyl)-acetamido) propanamide (2)

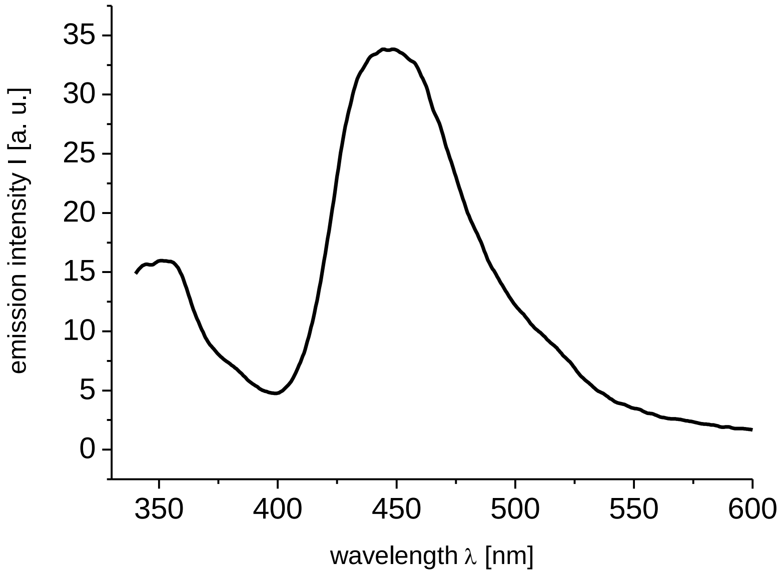

Emission spectrum of **2** recorded in CH_2Cl_2, T = 298 K, c = 2.86 · 10^{-6} $molL^{-1}$.

6.2 2-(*N*-(3-(10*H*-Phenothiazin-10-yl)propyl)acetamido)-*N*-(*tert*-butyl)-2-(9,10-dioxo-9,10-dihydroanthracen-2-yl) acetamide (8a)

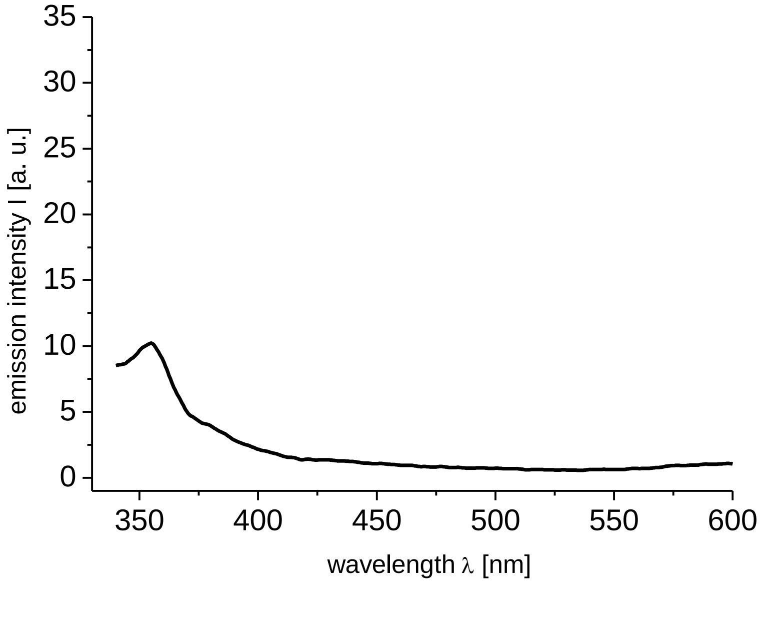

Emission spectrum of **8a** recorded in CH_2Cl_2, T = 298 K, c = 1.97 · 10^{-6} $molL^{-1}$.

6.3 2-(*N*-(4-((10*H*-Phenothiazin-10-yl)methyl)benzyl)acetamido)-*N*-(*tert*-butyl)-2-(9,10-dioxo-9,10-dihydroanthracen-2-yl) acetamide (8b)

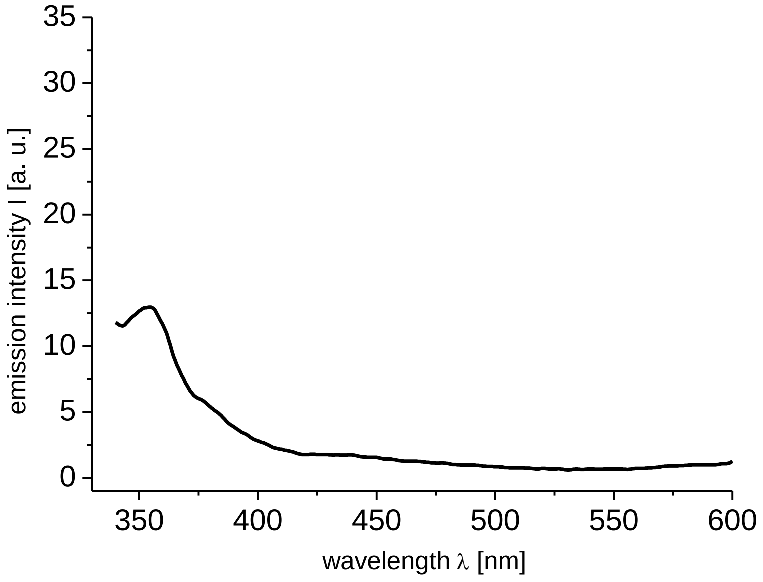

Emission spectrum of **8a** recorded in CH_2Cl_2, T = 298 K, c = 2.27 · 10^{-6} $molL^{-1}$.

6.4 *N*-(*tert*-Butyl)-2-(*N*-(4-(10-hexyl-10*H*-phenothiazin-3-yl)benzyl)-acetamido) propanamide (10a)

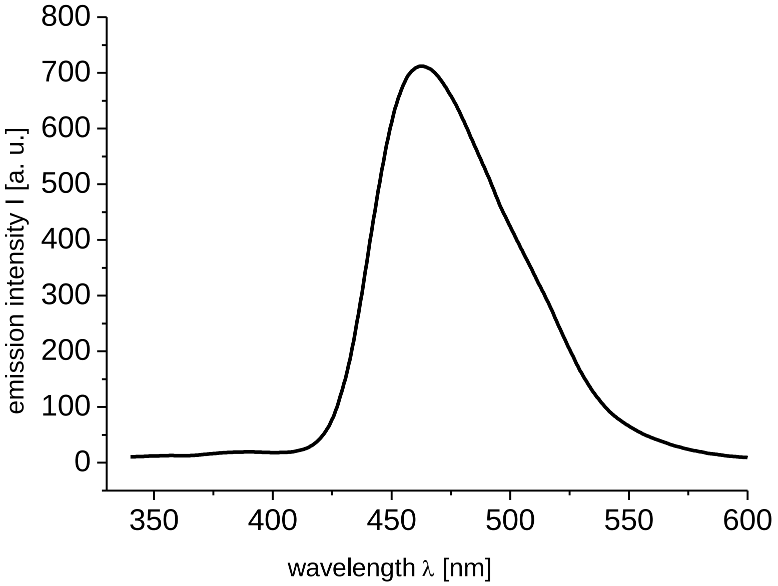

Emission spectrum of **10a** recorded in CH_2Cl_2, T = 298 K, c = 1.09 · 10^{-6} $molL^{-1}$.

6.5 *N*-(*tert*-Butyl)-2-(9,10-dioxo-9,10-dihydroanthracen-2-yl)-2-(*N*-(4-(10-hexyl-10*H*-phenothiazin-3-yl)benzyl)acetamido) acetamide (8c)

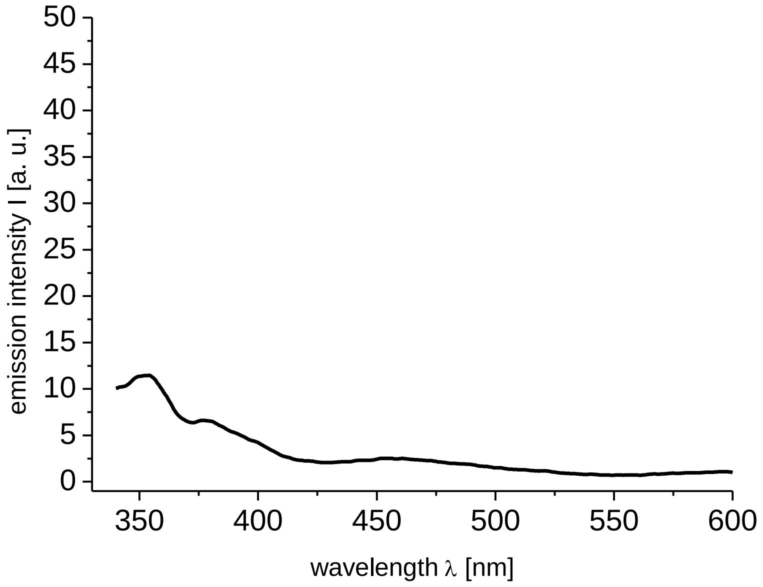

Emission spectrum of **8c** recorded in CH_2Cl_2, T = 298 K, c = 1.89 · 10^{-6} $molL^{-1}$.

6.6 *N*-(*tert*-Butyl)-2-(*N*-((10,10'-dihexyl-10*H*,10'*H*-[3,3'-biphenothiazin]-7-yl)methyl)acetamido)-2-(9,10-dioxo-9,10-dihydroanthracen-2-yl) acetamide (8d)

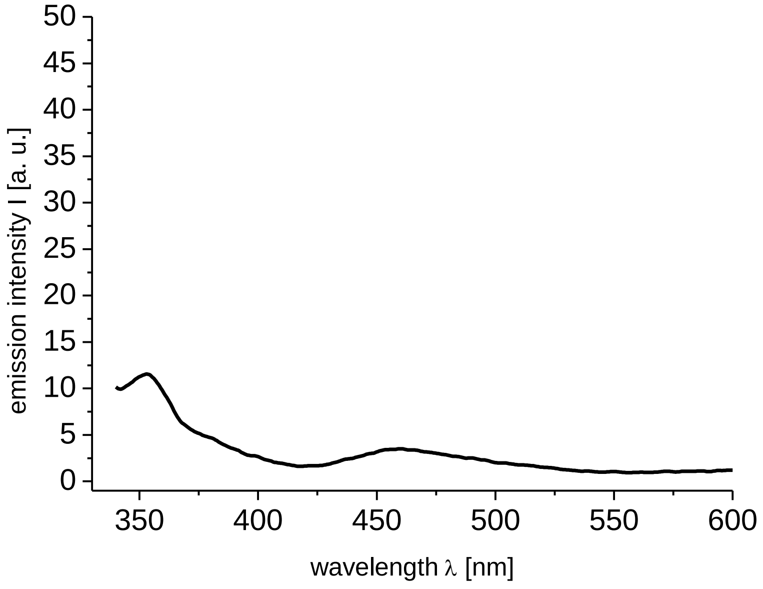

Emission spectrum of **8d** recorded in CH_2Cl_2, T = 298 K, c = 6.7 · 10^{-7} $molL^{-1}$.

6.7 *N*-(*tert*-Butyl)-2-(*N*-((9-hexyl-9*H*-carbazol-3-yl)methyl)acetamido) propanamide (10b)

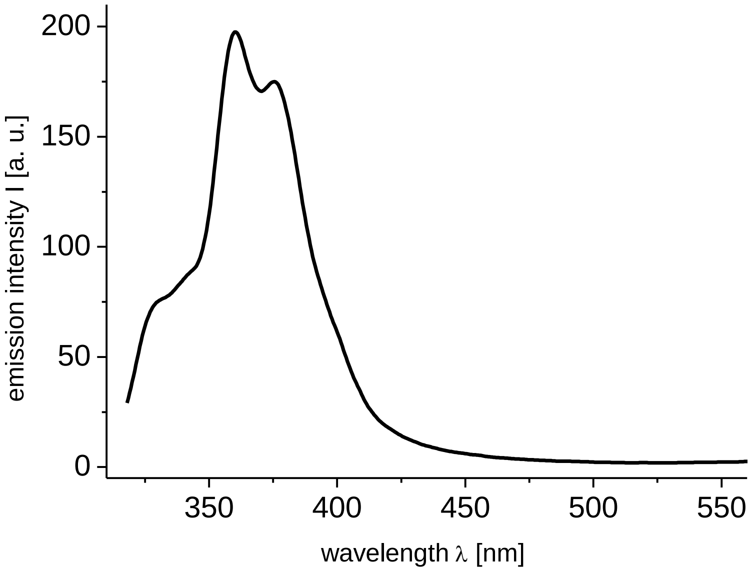

Emission spectrum of **10b** recorded in CH_2Cl_2, T = 298 K, c = 6.77 · 10^{-8} $molL^{-1}$.

6.8 *N*-(*tert*-Butyl)-2-(9,10-dioxo-9,10-dihydroanthracen-2-yl)-2-(*N*-((9-hexyl-9*H*-carbazol-3-yl)methyl)acetamido) acetamide (*8e*)

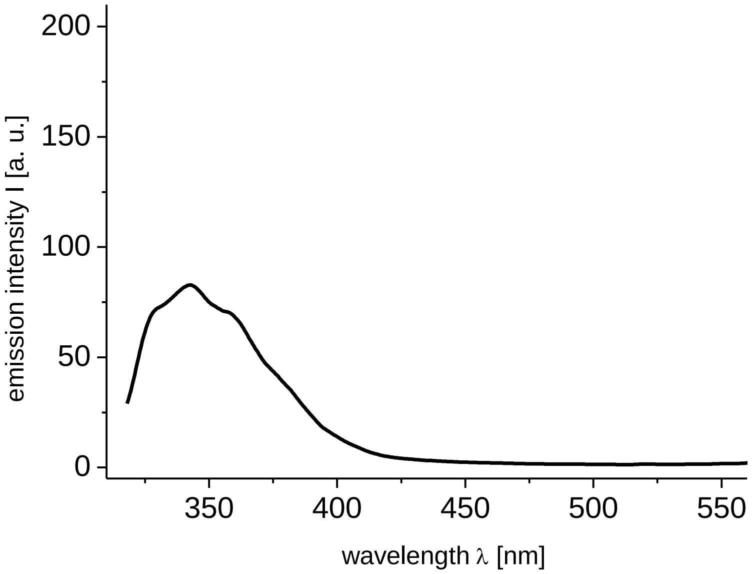

Emission spectrum of **8e** recorded in CH_2Cl_2, T = 298 K, c = 1.75 · 10^{-6} $molL^{-1}$.

6.9 *N*-(*tert*-Butyl)-2-(9,10-dioxo-9,10-dihydroanthracen-2-yl)-2-(*N*-(4-(9-hexyl-9*H*-carbazol-3-yl)benzyl)acetamido) acetamide (8f)

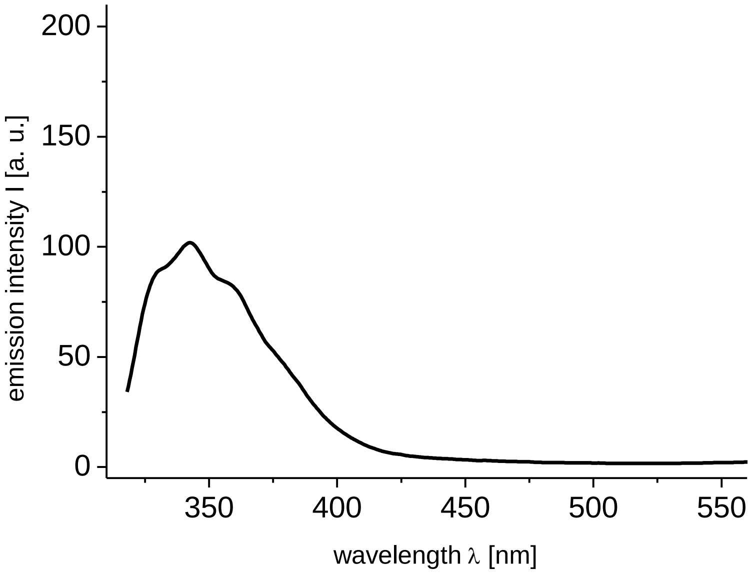

Emission spectrum of **8f** recorded in CH_2Cl_2, T = 298 K, c = 1.45 · 10^{-6} $molL^{-1}$.

7 Crystallographic Data of S(O)-1

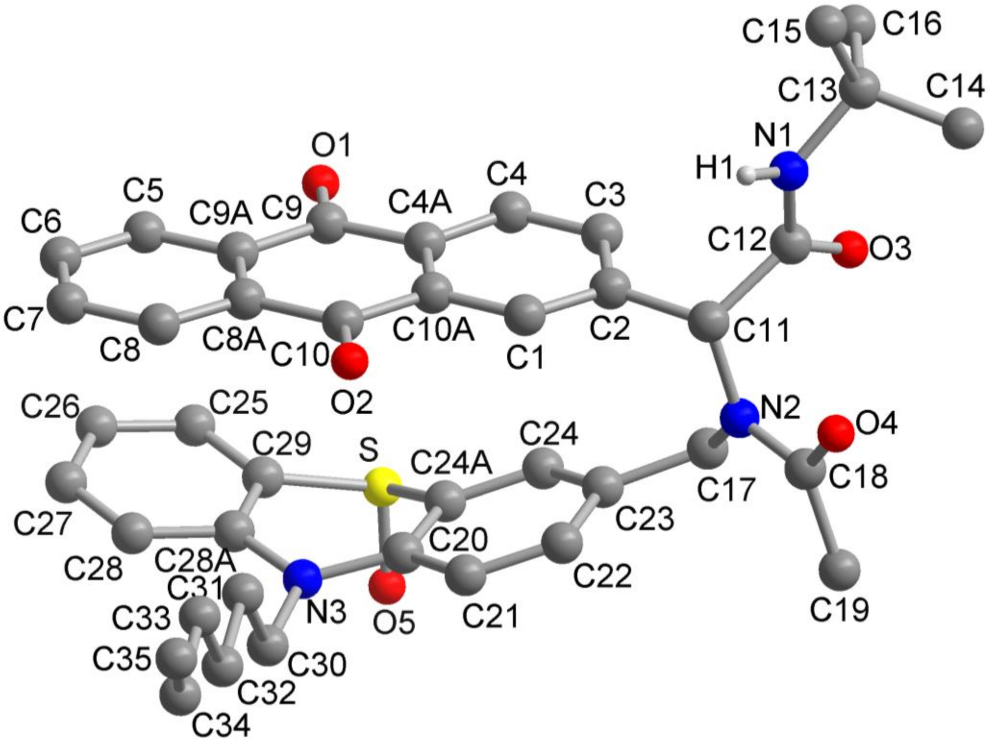

Table 1 Crystal data and structure refinement.

Empirical formula	$C_{41}H_{43}N_3O_5S$·0.88(O)	
Formula weight	703.84	
Temperature	293 K	
Wavelength	0.71073 Å [Mo-K_α]	
Crystal system	monoclinic	
Space group	$P2_1/c$	
Formula units per unit cell	Z = 4	
Unit cell dimensions	a = 15.3923(18) Å	α = 90 °
	b = 16.3424(19) Å	β = 105.855(7) °
	c = 15.8269(19) Å	γ = 90 °
Volume	3829.8(8) $Å^3$	
Density (calculated)	1.221 g/cm^3	
Absorption coefficient μ	0.13 mm^{-1}	
F (000)	1492	
Crystal habit	Block, brown	
Theta range for data collection	1.8 to 25.2°	
Limiting indices	-18≤h≤18, -19≤k≤19, -18≤l≤18	
Completeness to theta = 25.2	99.1%	
Reflections collected	33676	
Independent reflections collected	6817 (R(int) = 0.033)	
Observed reflections	5038 (I > 2σ(I))	
Refinement method	Full-matrix least-squares on F^2	
Data/restraints/parameters	6817 / 1 / 479	
Goodness-of-fit an F^2	1.02	
Final R indices (I > 2σ(I))	R1 = 0.068, wR2 = 0.139	
Largest difference peak and hole	0.30 $e^-/Å^3$ und -0.39 $e^-/Å^3$	

Table 2 Fractional atomic coordinates and isotropic or equivalent isotropic displacement parameters (Å^2).

	x	*y*	*z*	U_{iso}*/U_{eq}	**Occ. (<1)**
C1	0.55019 (13)	0.51990 (12)	0.26585 (13)	0.0439 (5)	
H1A	0.5310	0.4793	0.2237	0.053*	
C2	0.62643 (13)	0.56490 (12)	0.26672 (13)	0.0450 (5)	
C3	0.65684 (15)	0.62286 (13)	0.33262 (15)	0.0564 (6)	
H3	0.7099	0.6515	0.3360	0.068*	
C4	0.60913 (17)	0.63793 (14)	0.39249 (15)	0.0613 (6)	
H4	0.6299	0.6772	0.4359	0.074*	
C4A	0.53031 (15)	0.59550 (13)	0.38924 (14)	0.0532 (5)	
C5	0.3465 (2)	0.5812 (2)	0.50777 (18)	0.0802 (8)	
H5	0.3635	0.6235	0.5483	0.096*	
C6	0.2722 (2)	0.5343 (2)	0.5068 (2)	0.0933 (10)	
H6A	0.2394	0.5449	0.5470	0.112*	
C7	0.2457 (2)	0.4720 (2)	0.4473 (2)	0.0916 (9)	
H7	0.1949	0.4411	0.4471	0.110*	
C8	0.29407 (18)	0.45476 (18)	0.38742 (18)	0.0735 (7)	
H8	0.2763	0.4122	0.3474	0.088*	
C8A	0.36960 (16)	0.50172 (15)	0.38773 (14)	0.0583 (6)	
C9	0.47713 (18)	0.61519 (16)	0.45149 (16)	0.0660 (6)	
O1	0.49976 (16)	0.67105 (15)	0.50363 (15)	0.1086 (8)	
C9A	0.39631 (17)	0.56562 (15)	0.44820 (15)	0.0626 (6)	
C10	0.42145 (15)	0.48288 (14)	0.32338 (14)	0.0524 (5)	
O2	0.39915 (12)	0.42711 (11)	0.27074 (11)	0.0716 (5)	
C10A	0.50185 (14)	0.53388 (12)	0.32624 (13)	0.0458 (5)	
C11	0.67210 (13)	0.55057 (12)	0.19488 (14)	0.0458 (5)	
H11	0.6586	0.4942	0.1743	0.055*	
C12	0.77563 (15)	0.55815 (13)	0.22821 (15)	0.0531 (5)	
O3	0.81481 (12)	0.62099 (10)	0.21852 (14)	0.0807 (6)	
N1	0.81549 (12)	0.49123 (12)	0.26850 (14)	0.0601 (5)	
H1	0.782 (2)	0.4501 (18)	0.2695 (19)	0.090*	
C13	0.91342 (17)	0.47763 (18)	0.3038 (2)	0.0821 (9)	
C14	0.9593 (2)	0.4865 (3)	0.2314 (3)	0.1376 (17)	
H14A	0.9361	0.4460	0.1869	0.206*	
H14B	1.0232	0.4790	0.2550	0.206*	
H14C	0.9477	0.5401	0.2060	0.206*	
C15	0.9248 (2)	0.3921 (2)	0.3426 (3)	0.1389 (18)	
H15A	0.8900	0.3871	0.3843	0.208*	
H15B	0.9873	0.3825	0.3716	0.208*	
H15C	0.9041	0.3527	0.2966	0.208*	

C16	0.9513 (2)	0.5396 (3)	0.3765 (3)	0.1345 (16)
H16A	0.9424	0.5939	0.3527	0.202*
H16B	1.0147	0.5298	0.4012	0.202*
H16C	0.9207	0.5340	0.4215	0.202*
C17	0.60009 (14)	0.68542 (12)	0.13155 (15)	0.0502 (5)
H17A	0.6003	0.7197	0.0816	0.060*
H17B	0.6395	0.7107	0.1835	0.060*
N2	0.63651 (12)	0.60469 (10)	0.11902 (11)	0.0494 (4)
C18	0.64856 (16)	0.57786 (15)	0.04209 (15)	0.0588 (6)
O4	0.68790 (13)	0.51362 (11)	0.03928 (12)	0.0782 (5)
C19	0.6123 (2)	0.62849 (19)	-0.03990 (18)	0.0895 (9)
H19A	0.6510	0.6747	-0.0388	0.134*
H19B	0.5525	0.6472	-0.0423	0.134*
H19C	0.6102	0.5957	-0.0907	0.134*
C20	0.32958 (13)	0.67269 (12)	0.16556 (13)	0.0459 (5)
C21	0.35587 (14)	0.62186 (12)	0.10631 (14)	0.0494 (5)
H21	0.3152	0.5839	0.0739	0.059*
C22	0.44109 (15)	0.62705 (12)	0.09510 (14)	0.0496 (5)
H22	0.4564	0.5923	0.0549	0.060*
C23	0.50551 (14)	0.68232 (12)	0.14155 (13)	0.0460 (5)
C24	0.47905 (14)	0.73523 (12)	0.19744 (14)	0.0491 (5)
H24	0.5196	0.7742	0.2281	0.059*
C24A	0.39253 (14)	0.73137 (12)	0.20890 (14)	0.0473 (5)
C25	0.2716 (2)	0.78047 (18)	0.3887 (2)	0.0831 (8)
H25	0.3099	0.8197	0.4218	0.100*
C26	0.2031 (3)	0.7497 (2)	0.4182 (2)	0.1052 (12)
H26	0.1950	0.7666	0.4717	0.126*
C27	0.1459 (2)	0.6931 (2)	0.3673 (2)	0.0976 (11)
H27	0.0977	0.6731	0.3861	0.117*
C28	0.15802 (19)	0.66526 (16)	0.2898 (2)	0.0777 (8)
H28	0.1183	0.6267	0.2572	0.093*
C28A	0.22948 (15)	0.69417 (13)	0.25897 (16)	0.0560 (6)
S	0.36239 (4)	0.81010 (3)	0.27023 (4)	0.06028 (19)
O5	0.30794 (13)	0.86993 (10)	0.20487 (14)	0.0809 (6)
C29	0.28493 (16)	0.75389 (14)	0.30989 (16)	0.0594 (6)
N3	0.24520 (12)	0.66420 (11)	0.18221 (12)	0.0531 (4)
C30	0.17848 (15)	0.60977 (15)	0.12509 (16)	0.0620 (6)
H30A	0.1828	0.6155	0.0654	0.074*
H30B	0.1186	0.6275	0.1258	0.074*
C31	0.18893 (18)	0.51977 (15)	0.15028 (18)	0.0682 (7)
H31A	0.2512	0.5035	0.1578	0.082*
H31B	0.1752	0.5122	0.2061	0.082*

C32	0.1283 (2)	0.4661 (2)	0.0826 (2)	0.1046 (11)	
H32A	0.1411	0.4748	0.0266	0.126*	
H32B	0.0660	0.4819	0.0760	0.126*	
C33	0.1390 (3)	0.3761 (2)	0.1054 (4)	0.158 (2)	
H33A	0.1252	0.3666	0.1609	0.190*	
H33B	0.2011	0.3596	0.1121	0.190*	
C34	0.0755 (7)	0.3245 (4)	0.0329 (7)	0.313 (6)	
H34A	0.0135	0.3342	0.0336	0.376*	
H34B	0.0815	0.3419	-0.0238	0.376*	
C35	0.0916 (8)	0.2510 (6)	0.0420 (8)	0.398 (8)	
H35A	0.1169	0.2389	0.1031	0.597*	
H35B	0.1340	0.2360	0.0100	0.597*	
H35C	0.0367	0.2206	0.0199	0.597*	
O6A	0.8088 (15)	0.7619 (10)	0.1311 (15)	0.255 (12)	0.49 (3)
O6B	0.886 (3)	0.7738 (13)	0.174 (2)	0.355 (18)	0.39 (3)

Table 3 Atomic displacement parameters (Å^2)

	U^{11}	U^{22}	U^{33}	U^{12}	U^{13}	U^{23}
C1	0.0485 (12)	0.0389 (10)	0.0424 (11)	0.0014 (9)	0.0094 (9)	-0.0017 (8)
C2	0.0455 (11)	0.0395 (11)	0.0482 (12)	0.0030 (9)	0.0100 (9)	0.0012 (9)
C3	0.0531 (13)	0.0518 (13)	0.0613 (14)	-0.0057 (10)	0.0105 (11)	-0.0084 (10)
C4	0.0695 (16)	0.0552 (13)	0.0546 (14)	-0.0028 (12)	0.0093 (12)	-0.0161 (11)
C4A	0.0596 (14)	0.0517 (12)	0.0458 (12)	0.0086 (11)	0.0105 (10)	-0.0026 (10)
C5	0.088 (2)	0.098 (2)	0.0632 (16)	0.0246 (17)	0.0339 (15)	0.0041 (15)
C6	0.095 (2)	0.121 (3)	0.081 (2)	0.029 (2)	0.0536 (18)	0.015 (2)
C7	0.0765 (19)	0.118 (3)	0.093 (2)	0.0103 (18)	0.0447 (17)	0.024 (2)
C8	0.0655 (16)	0.0915 (19)	0.0695 (16)	0.0034 (14)	0.0285 (13)	0.0118 (14)
C8A	0.0564 (14)	0.0704 (15)	0.0503 (13)	0.0088 (11)	0.0184 (11)	0.0119 (11)
C9	0.0732 (16)	0.0704 (16)	0.0543 (14)	0.0106 (13)	0.0173 (12)	-0.0111 (12)
O1	0.1183 (17)	0.1175 (17)	0.1019 (16)	-0.0175 (14)	0.0504 (14)	-0.0636 (14)
C9A	0.0692 (16)	0.0708 (16)	0.0507 (13)	0.0182 (13)	0.0215 (12)	0.0040 (11)
C10	0.0557 (13)	0.0551 (13)	0.0472 (12)	0.0024 (10)	0.0156 (10)	0.0024 (10)
O2	0.0720 (11)	0.0755 (11)	0.0737 (11)	-0.0207 (9)	0.0310 (9)	-0.0195 (9)
C10A	0.0481 (12)	0.0462 (11)	0.0418 (11)	0.0050 (9)	0.0100 (9)	0.0017 (9)
C11	0.0443 (11)	0.0365 (10)	0.0564 (12)	0.0015 (8)	0.0135 (9)	0.0001 (9)
C12	0.0474 (12)	0.0467 (12)	0.0661 (14)	-0.0030 (10)	0.0169 (10)	0.0045 (10)
O3	0.0616 (11)	0.0571 (10)	0.1186 (15)	-0.0150 (8)	0.0164 (10)	0.0193 (10)
N1	0.0393 (10)	0.0536 (11)	0.0859 (14)	0.0007 (8)	0.0147 (10)	0.0155 (10)
C13	0.0419 (14)	0.0861 (19)	0.113 (2)	0.0059 (13)	0.0117 (14)	0.0293 (17)
C14	0.071 (2)	0.183 (4)	0.180 (4)	0.030 (2)	0.069 (3)	0.044 (3)
C15	0.070 (2)	0.109 (3)	0.225 (5)	0.0291 (19)	0.018 (3)	0.078 (3)

C16	0.079 (2)	0.150 (4)	0.140 (3)	-0.017 (2)	-0.027 (2)	0.019 (3)
C17	0.0524 (12)	0.0402 (11)	0.0614 (13)	0.0063 (9)	0.0211 (10)	0.0028 (9)
N2	0.0528 (10)	0.0445 (9)	0.0537 (10)	0.0092 (8)	0.0194 (8)	-0.0005 (8)
C18	0.0624 (14)	0.0594 (14)	0.0562 (14)	0.0034 (11)	0.0188 (11)	-0.0084 (11)
O4	0.1015 (14)	0.0643 (11)	0.0729 (12)	0.0189 (10)	0.0306 (10)	-0.0162 (9)
C19	0.118 (3)	0.094 (2)	0.0593 (16)	0.0213 (18)	0.0273 (16)	0.0036 (15)
C20	0.0440 (11)	0.0422 (11)	0.0492 (12)	0.0019 (9)	0.0089 (9)	-0.0007 (9)
C21	0.0501 (12)	0.0465 (12)	0.0482 (12)	-0.0024 (9)	0.0075 (10)	-0.0075 (9)
C22	0.0584 (13)	0.0435 (11)	0.0474 (12)	0.0063 (10)	0.0152 (10)	-0.0031 (9)
C23	0.0485 (12)	0.0379 (10)	0.0528 (12)	0.0066 (9)	0.0158 (9)	0.0034 (9)
C24	0.0478 (12)	0.0388 (11)	0.0598 (13)	-0.0001 (9)	0.0134 (10)	-0.0053 (9)
C24A	0.0466 (12)	0.0393 (11)	0.0569 (13)	0.0028 (9)	0.0157 (10)	-0.0065 (9)
C25	0.091 (2)	0.0817 (19)	0.089 (2)	-0.0163 (16)	0.0454 (17)	-0.0317 (16)
C26	0.128 (3)	0.101 (2)	0.113 (3)	-0.023 (2)	0.079 (2)	-0.037 (2)
C27	0.107 (2)	0.090 (2)	0.124 (3)	-0.0231 (18)	0.080 (2)	-0.024 (2)
C28	0.0762 (18)	0.0653 (16)	0.104 (2)	-0.0171 (13)	0.0465 (16)	-0.0185 (15)
C28A	0.0524 (13)	0.0486 (12)	0.0709 (15)	0.0009 (10)	0.0235 (11)	-0.0036 (11)
S	0.0571 (4)	0.0474 (3)	0.0830 (4)	-0.0073 (3)	0.0305 (3)	-0.0223 (3)
O5	0.0911 (13)	0.0434 (9)	0.1238 (16)	0.0124 (9)	0.0560 (12)	0.0020 (9)
C29	0.0585 (14)	0.0550 (13)	0.0695 (15)	-0.0031 (11)	0.0257 (12)	-0.0135 (11)
N3	0.0449 (10)	0.0526 (10)	0.0609 (11)	-0.0046 (8)	0.0131 (8)	-0.0080 (9)
C30	0.0466 (13)	0.0689 (15)	0.0645 (15)	-0.0093 (11)	0.0050 (11)	-0.0072 (12)
C31	0.0656 (15)	0.0650 (15)	0.0752 (16)	-0.0150 (12)	0.0214 (13)	-0.0108 (13)
C32	0.102 (2)	0.097 (2)	0.115 (3)	-0.0368 (19)	0.029 (2)	-0.045 (2)
C33	0.153 (4)	0.091 (3)	0.233 (6)	-0.044 (3)	0.055 (4)	-0.064 (3)
C34	0.333 (11)	0.105 (4)	0.410 (14)	-0.022 (6)	-0.056 (10)	-0.094 (7)
C35	0.414 (16)	0.202 (9)	0.486 (19)	-0.033 (11)	-0.031 (14)	-0.136 (12)
O6A	0.202 (15)	0.160 (11)	0.44 (2)	0.032 (9)	0.155 (15)	0.156 (12)
O6B	0.34 (4)	0.227 (17)	0.53 (4)	-0.159 (19)	0.17 (3)	0.027 (18)

Table 4 Bond length (Å) and angles (°)

C1—C10A	1.381 (3)	N2—C18	1.354 (3)
C1—C2	1.382 (3)	C18—O4	1.219 (3)
C1—H1A	0.9300	C18—C19	1.511 (4)
C2—C3	1.392 (3)	C19—H19A	0.9600
C2—C11	1.509 (3)	C19—H19B	0.9600
C3—C4	1.370 (3)	C19—H19C	0.9600
C3—H3	0.9300	C20—C21	1.393 (3)
C4—C4A	1.386 (3)	C20—N3	1.401 (3)
C4—H4	0.9300	C20—C24A	1.402 (3)
C4A—C10A	1.401 (3)	C21—C22	1.373 (3)

C4A—C9	1.478 (3)	C21—H21	0.9300
C5—C6	1.374 (5)	C22—C23	1.393 (3)
C5—C9A	1.392 (3)	C22—H22	0.9300
C5—H5	0.9300	C23—C24	1.376 (3)
C6—C7	1.371 (5)	C24—C24A	1.394 (3)
C6—H6A	0.9300	C24—H24	0.9300
C7—C8	1.384 (4)	C24A—S	1.749 (2)
C7—H7	0.9300	C25—C26	1.360 (4)
C8—C8A	1.392 (3)	C25—C29	1.389 (3)
C8—H8	0.9300	C25—H25	0.9300
C8A—C9A	1.400 (3)	C26—C27	1.376 (4)
C8A—C10	1.488 (3)	C26—H26	0.9300
C9—O1	1.216 (3)	C27—C28	1.368 (4)
C9—C9A	1.473 (4)	C27—H27	0.9300
C10—O2	1.219 (3)	C28—C28A	1.402 (3)
C10—C10A	1.482 (3)	C28—H28	0.9300
C11—N2	1.471 (3)	C28A—N3	1.391 (3)
C11—C12	1.541 (3)	C28A—C29	1.398 (3)
C11—H11	0.9800	S—O5	1.502 (2)
C12—O3	1.222 (3)	S—C29	1.752 (2)
C12—N1	1.329 (3)	N3—C30	1.468 (3)
N1—C13	1.474 (3)	C30—C31	1.521 (3)
N1—H1	0.85 (3)	C30—H30A	0.9700
C13—C14	1.508 (5)	C30—H30B	0.9700
C13—C15	1.517 (4)	C31—C32	1.498 (4)
C13—C16	1.525 (5)	C31—H31A	0.9700
C14—H14A	0.9600	C31—H31B	0.9700
C14—H14B	0.9600	C32—C33	1.513 (5)
C14—H14C	0.9600	C32—H32A	0.9700
C15—H15A	0.9600	C32—H32B	0.9700
C15—H15B	0.9600	C33—C34	1.540 (8)
C15—H15C	0.9600	C33—H33A	0.9700
C16—H16A	0.9600	C33—H33B	0.9700
C16—H16B	0.9600	C34—C35	1.227 (10)
C16—H16C	0.9600	C34—H34A	0.9700
C17—N2	1.468 (2)	C34—H34B	0.9700
C17—C23	1.508 (3)	C35—H35A	0.9600
C17—H17A	0.9700	C35—H35B	0.9600
C17—H17B	0.9700	C35—H35C	0.9600
C10A—C1—C2	121.45 (19)	C17—N2—C11	120.06 (16)
C10A—C1—H1A	119.3	O4—C18—N2	120.2 (2)

C2—C1—H1A	119.3	O4—C18—C19	120.7 (2)
C1—C2—C3	118.71 (19)	N2—C18—C19	119.1 (2)
C1—C2—C11	118.62 (18)	C18—C19—H19A	109.5
C3—C2—C11	122.65 (19)	C18—C19—H19B	109.5
C4—C3—C2	120.4 (2)	H19A—C19—H19B	109.5
C4—C3—H3	119.8	C18—C19—H19C	109.5
C2—C3—H3	119.8	H19A—C19—H19C	109.5
C3—C4—C4A	120.9 (2)	H19B—C19—H19C	109.5
C3—C4—H4	119.6	C21—C20—N3	121.49 (18)
C4A—C4—H4	119.6	C21—C20—C24A	116.63 (19)
C4—C4A—C10A	119.1 (2)	N3—C20—C24A	121.87 (18)
C4—C4A—C9	120.3 (2)	C22—C21—C20	120.89 (19)
C10A—C4A—C9	120.6 (2)	C22—C21—H21	119.6
C6—C5—C9A	120.1 (3)	C20—C21—H21	119.6
C6—C5—H5	120.0	C21—C22—C23	122.61 (19)
C9A—C5—H5	120.0	C21—C22—H22	118.7
C7—C6—C5	120.8 (3)	C23—C22—H22	118.7
C7—C6—H6A	119.6	C24—C23—C22	116.98 (19)
C5—C6—H6A	119.6	C24—C23—C17	120.69 (19)
C6—C7—C8	120.4 (3)	C22—C23—C17	122.33 (18)
C6—C7—H7	119.8	C23—C24—C24A	121.10 (19)
C8—C7—H7	119.8	C23—C24—H24	119.5
C7—C8—C8A	119.4 (3)	C24A—C24—H24	119.5
C7—C8—H8	120.3	C24—C24A—C20	121.63 (18)
C8A—C8—H8	120.3	C24—C24A—S	116.54 (15)
C8—C8A—C9A	120.2 (2)	C20—C24A—S	121.47 (15)
C8—C8A—C10	119.2 (2)	C26—C25—C29	120.7 (3)
C9A—C8A—C10	120.7 (2)	C26—C25—H25	119.6
O1—C9—C9A	121.3 (2)	C29—C25—H25	119.6
O1—C9—C4A	120.5 (3)	C25—C26—C27	118.7 (3)
C9A—C9—C4A	118.2 (2)	C25—C26—H26	120.7
C5—C9A—C8A	119.1 (3)	C27—C26—H26	120.7
C5—C9A—C9	119.4 (2)	C28—C27—C26	121.8 (3)
C8A—C9A—C9	121.4 (2)	C28—C27—H27	119.1
O2—C10—C10A	121.2 (2)	C26—C27—H27	119.1
O2—C10—C8A	121.3 (2)	C27—C28—C28A	120.8 (3)
C10A—C10—C8A	117.5 (2)	C27—C28—H28	119.6
C1—C10A—C4A	119.25 (19)	C28A—C28—H28	119.6
C1—C10A—C10	119.27 (18)	N3—C28A—C29	121.6 (2)
C4A—C10A—C10	121.48 (19)	N3—C28A—C28	121.7 (2)
N2—C11—C2	112.04 (16)	C29—C28A—C28	116.6 (2)
N2—C11—C12	110.53 (16)	O5—S—C24A	106.22 (10)

C2—C11—C12	112.37 (17)	O5—S—C29	106.37 (11)
N2—C11—H11	107.2	C24A—S—C29	97.06 (10)
C2—C11—H11	107.2	C25—C29—C28A	121.3 (2)
C12—C11—H11	107.2	C25—C29—S	116.12 (19)
O3—C12—N1	125.0 (2)	C28A—C29—S	121.85 (18)
O3—C12—C11	121.64 (19)	C28A—N3—C20	121.39 (18)
N1—C12—C11	113.38 (18)	C28A—N3—C30	119.46 (18)
C12—N1—C13	126.6 (2)	C20—N3—C30	118.20 (18)
C12—N1—H1	117 (2)	N3—C30—C31	114.73 (19)
C13—N1—H1	116 (2)	N3—C30—H30A	108.6
N1—C13—C14	109.9 (3)	C31—C30—H30A	108.6
N1—C13—C15	106.5 (2)	N3—C30—H30B	108.6
C14—C13—C15	111.8 (3)	C31—C30—H30B	108.6
N1—C13—C16	109.1 (3)	H30A—C30—H30B	107.6
C14—C13—C16	110.4 (3)	C32—C31—C30	112.3 (2)
C15—C13—C16	109.1 (3)	C32—C31—H31A	109.2
C13—C14—H14A	109.5	C30—C31—H31A	109.2
C13—C14—H14B	109.5	C32—C31—H31B	109.2
H14A—C14—H14B	109.5	C30—C31—H31B	109.2
C13—C14—H14C	109.5	H31A—C31—H31B	107.9
H14A—C14—H14C	109.5	C31—C32—C33	113.1 (3)
H14B—C14—H14C	109.5	C31—C32—H32A	109.0
C13—C15—H15A	109.5	C33—C32—H32A	109.0
C13—C15—H15B	109.5	C31—C32—H32B	109.0
H15A—C15—H15B	109.5	C33—C32—H32B	109.0
C13—C15—H15C	109.5	H32A—C32—H32B	107.8
H15A—C15—H15C	109.5	C32—C33—C34	110.4 (5)
H15B—C15—H15C	109.5	C32—C33—H33A	109.6
C13—C16—H16A	109.5	C34—C33—H33A	109.6
C13—C16—H16B	109.5	C32—C33—H33B	109.6
H16A—C16—H16B	109.5	C34—C33—H33B	109.6
C13—C16—H16C	109.5	H33A—C33—H33B	108.1
H16A—C16—H16C	109.5	C35—C34—C33	112.4 (9)
H16B—C16—H16C	109.5	C35—C34—H34A	109.1
N2—C17—C23	113.54 (17)	C33—C34—H34A	109.1
N2—C17—H17A	108.9	C35—C34—H34B	109.1
C23—C17—H17A	108.9	C33—C34—H34B	109.1
N2—C17—H17B	108.9	H34A—C34—H34B	107.9
C23—C17—H17B	108.9	C34—C35—H35A	109.5
H17A—C17—H17B	107.7	C34—C35—H35B	109.5
C18—N2—C17	124.32 (18)	C34—C35—H35C	109.5
C18—N2—C11	115.30 (17)		

Table 5 Torsion angles (°)

C10A—C1—C2—C3	2.9 (3)	C12—C11—N2—C18	76.4 (2)
C10A—C1—C2—C11	-175.66 (18)	C2—C11—N2—C17	28.8 (2)
C1—C2—C3—C4	-3.5 (3)	C12—C11—N2—C17	-97.3 (2)
C11—C2—C3—C4	175.0 (2)	C17—N2—C18—O4	172.3 (2)
C2—C3—C4—C4A	0.6 (4)	C11—N2—C18—O4	-1.2 (3)
C3—C4—C4A—C10A	3.0 (3)	C17—N2—C18—C19	-8.0 (3)
C3—C4—C4A—C9	-177.0 (2)	C11—N2—C18—C19	178.6 (2)
C9A—C5—C6—C7	-0.3 (5)	N3—C20—C21—C22	-175.43 (19)
C5—C6—C7—C8	0.6 (5)	C24A—C20—C21—C22	3.2 (3)
C6—C7—C8—C8A	-0.5 (4)	C20—C21—C22—C23	0.1 (3)
C7—C8—C8A—C9A	0.1 (4)	C21—C22—C23—C24	-2.9 (3)
C7—C8—C8A—C10	179.9 (2)	C21—C22—C23—C17	177.5 (2)
C4—C4A—C9—O1	3.2 (4)	N2—C17—C23—C24	144.1 (2)
C10A—C4A—C9—O1	-176.8 (2)	N2—C17—C23—C22	-36.3 (3)
C4—C4A—C9—C9A	-177.0 (2)	C22—C23—C24—C24A	2.1 (3)
C10A—C4A—C9—C9A	3.0 (3)	C17—C23—C24—C24A	-178.26 (19)
C6—C5—C9A—C8A	0.0 (4)	C23—C24—C24A—C20	1.3 (3)
C6—C5—C9A—C9	-178.5 (3)	C23—C24—C24A—S	-171.90 (16)
C8—C8A—C9A—C5	0.1 (4)	C21—C20—C24A—C24	-3.9 (3)
C10—C8A—C9A—C5	-179.6 (2)	N3—C20—C24A—C24	174.72 (19)
C8—C8A—C9A—C9	178.6 (2)	C21—C20—C24A—S	168.91 (16)
C10—C8A—C9A—C9	-1.2 (3)	N3—C20—C24A—S	-12.5 (3)
O1—C9—C9A—C5	-2.1 (4)	C29—C25—C26—C27	-1.5 (5)
C4A—C9—C9A—C5	178.1 (2)	C25—C26—C27—C28	2.1 (6)
O1—C9—C9A—C8A	179.4 (3)	C26—C27—C28—C28A	-0.4 (5)
C4A—C9—C9A—C8A	-0.4 (3)	C27—C28—C28A—N3	177.3 (3)
C8—C8A—C10—O2	-0.2 (3)	C27—C28—C28A—C29	-1.9 (4)
C9A—C8A—C10—O2	179.6 (2)	C24—C24A—S—O5	98.66 (18)
C8—C8A—C10—C10A	-179.5 (2)	C20—C24A—S—O5	-74.5 (2)
C9A—C8A—C10—C10A	0.2 (3)	C24—C24A—S—C29	-151.96 (18)
C2—C1—C10A—C4A	0.6 (3)	C20—C24A—S—C29	34.9 (2)
C2—C1—C10A—C10	-178.94 (18)	C26—C25—C29—C28A	-0.9 (5)
C4—C4A—C10A—C1	-3.5 (3)	C26—C25—C29—S	169.5 (3)
C9—C4A—C10A—C1	176.5 (2)	N3—C28A—C29—C25	-176.7 (2)
C4—C4A—C10A—C10	176.0 (2)	C28—C28A—C29—C25	2.5 (4)
C9—C4A—C10A—C10	-4.0 (3)	N3—C28A—C29—S	13.5 (3)
O2—C10—C10A—C1	2.6 (3)	C28—C28A—C29—S	-167.3 (2)
C8A—C10—C10A—C1	-178.06 (18)	O5—S—C29—C25	-96.6 (2)
O2—C10—C10A—C4A	-176.9 (2)	C24A—S—C29—C25	154.1 (2)

C8A—C10—C10A—C4A	2.4 (3)	O5—S—C29—C28A	73.7 (2)
C1—C2—C11—N2	90.4 (2)	C24A—S—C29—C28A	-35.6 (2)
C3—C2—C11—N2	-88.1 (2)	C29—C28A—N3—C20	19.4 (3)
C1—C2—C11—C12	-144.41 (18)	C28—C28A—N3—C20	-159.7 (2)
C3—C2—C11—C12	37.1 (3)	C29—C28A—N3—C30	-172.0 (2)
N2—C11—C12—O3	27.8 (3)	C28—C28A—N3—C30	8.9 (3)
C2—C11—C12—O3	-98.2 (3)	C21—C20—N3—C28A	158.6 (2)
N2—C11—C12—N1	-153.23 (19)	C24A—C20—N3—C28A	-20.0 (3)
C2—C11—C12—N1	80.8 (2)	C21—C20—N3—C30	-10.2 (3)
O3—C12—N1—C13	-4.2 (4)	C24A—C20—N3—C30	171.2 (2)
C11—C12—N1—C13	176.8 (2)	C28A—N3—C30—C31	-85.4 (3)
C12—N1—C13—C14	-58.4 (4)	C20—N3—C30—C31	83.6 (3)
C12—N1—C13—C15	-179.6 (3)	N3—C30—C31—C32	-171.5 (2)
C12—N1—C13—C16	62.8 (4)	C30—C31—C32—C33	178.8 (3)
C23—C17—N2—C18	107.8 (2)	C31—C32—C33—C34	-179.5 (6)
C23—C17—N2—C11	-79.0 (2)	C32—C33—C34—C35	169.6 (12)
C2—C11—N2—C18	-157.42 (18)		

Table 6 Hydrogen-bond geometry (Å, º)

D—H···*A*	*D*—H	H···*A*	*D*···*A*	*D*—H···*A*
N1—H1···O5^{i}	0.85 (3)	2.02 (3)	2.856 (2)	165 (3)

Symmetry code: (i) -*x*+1, *y*-1/2, -*z*+1/2.

π–π Stacking interactions – intramolecular and between an inversion symmetry related pair

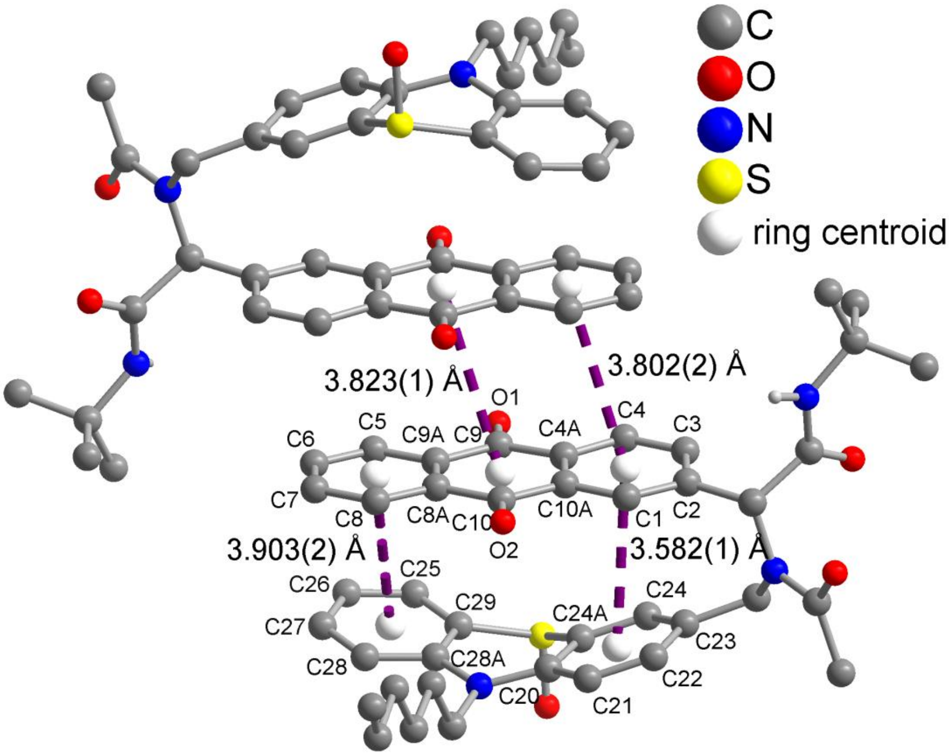
C
O
N
S
ring centroid
3.823(1) Å
3.802(2) Å
3.903(2) Å
3.582(1) Å
O1
C6
C5
C9A
C9
C4A
C4
C3
C7
C8
C8A
C10
C10A
C1
C2
O2
C26
C25
C29
C24A
C24
C27
C23
C28
C28A
C20
C22
C21

Table 7 Analysis of Short Ring-π-π Interactions with Cg-Cg Distances < 6.0 Angstrom and Beta < 60.0 Deg.

==
==

- Cg(I) = Plane number I (= ring number in () above)
- Alpha = Dihedral Angle between Planes I and J (Deg)
- Beta = Angle Cg(I)-->Cg(J) or Cg(I)-->Me vector and normal to plane I (Deg)
- Gamma = Angle Cg(I)-->Cg(J) vector and normal to plane J (Deg)
- Cg-Cg = Distance between ring Centroids (Ang.)
- CgI_Perp = Perpendicular distance of Cg(I) on ring J (Ang.)
- CgJ_Perp = Perpendicular distance of Cg(J) on ring I (Ang.)
- Slippage = Distance between Cg(I) and Perpendicular Projection of Cg(J) on Ring I (Ang).
- P,Q,R,S = J-Plane Parameters for Carth. Coord. (Xo, Yo, Zo)

Cg(I)	Res(I)	Cg(J)	ARU(J)	Cg-Cg	Alpha	Beta	Gamma	CgI_Perp	CgJ_Perp	Slippage
Cg(2)	[1]	Cg(3)	3666.01	5.0783(14)	3.11(10)	47.80	46.54	-3.4931(9)	-3.4116(9)	
Cg(2)	[1]	Cg(4)	3666.01	3.8019(15)	3.79(12)	26.62	24.32	-3.4647(9)	-3.3989(12)	
Cg(2)	[1]	Cg(5)	1555.01	3.5820(13)	17.04(10)	16.29	23.49	3.2852(9)	3.4382(8)	
Cg(3)	[1]	Cg(2)	3666.01	5.0783(14)	3.11(10)	46.54	47.80	-3.4115(9)	-3.4930(9)	
Cg(3)	[1]	Cg(3)	3666.01	3.8225(14)	0	23.95	23.95	-3.4935(9)	-3.4935(9)	1.551
Cg(3)	[1]	Cg(4)	3666.01	3.9352(16)	1.17(12)	28.10	26.95	-3.5080(9)	-3.4713(12)	
Cg(3)	[1]	Cg(5)	1555.01	4.1909(13)	16.29(10)	33.76	18.44	3.9757(9)	3.4841(8)	
Cg(3)	[1]	Cg(6)	1555.01	4.4827(17)	11.32(12)	34.96	25.10	-4.0592(10)	3.6738(12)	
Cg(4)	[1]	Cg(2)	3666.01	3.8019(15)	3.79(12)	24.32	26.62	-3.3989(12)	-3.4646(9)	
Cg(4)	[1]	Cg(3)	3666.01	3.9353(16)	1.17(12)	26.95	28.10	-3.4713(12)	-3.5081(9)	
Cg(4)	[1]	Cg(4)	3666.01	5.3445(18)	0	48.68	48.68	-3.5287(12)	-3.5287(12)	4.014
Cg(4)	[1]	Cg(5)	1555.01	5.8871(16)	17.24(12)	54.33	37.23	4.6874(12)	3.4330(8)	
Cg(4)	[1]	Cg(6)	1555.01	3.9027(17)	11.29(14)	19.52	13.54	-3.7943(12)	3.6783(12)	
Cg(5)	[1]	Cg(2)	1555.01	3.5821(13)	17.04(10)	23.49	16.29	3.4382(8)	3.2853(9)	
Cg(5)	[1]	Cg(3)	1555.01	4.1909(13)	16.29(10)	18.44	33.76	3.4841(8)	3.9756(9)	
Cg(5)	[1]	Cg(4)	1555.01	5.8872(16)	17.24(12)	37.23	54.33	3.4330(8)	4.6873(12)	
Cg(5)	[1]	Cg(4)	4564.01	5.8877(16)	83.23(12)	16.12	81.51	0.8694(8)	-5.6562(11)	
Cg(5)	[1]	Cg(6)	4564.01	5.3360(16)	86.90(12)	25.13	67.97	-2.0019(8)	-4.8308(12)	
Cg(6)	[1]	Cg(3)	1555.01	4.4826(17)	11.32(12)	25.10	34.96	3.6738(12)	-4.0592(10)	
Cg(6)	[1]	Cg(4)	1555.01	3.9026(17)	11.29(14)	13.54	19.52	3.6783(12)	-3.7942(12)	
				Min or Max 3.582	0.00	13.54	81.51	-4.059	-5.656	

inter-molecular between centrosymmetric pair

intra-molecular

[3666] = 1-X,1-Y,1-Z

[1555] = X,Y,Z

[4564] = X,3/2-Y,-1/2+Z

Ring with centroid (Cg)

Cg(2) = C1-C2-C3-C4-C4a-C10a

Cg(3) = C4a-C8a-C9-C9a-C10-C10a

Cg(4) = C5-C6-C7-C8-C8a-C9a

Cg(5) = C20-C21-C22-C23-C24-C24a

Cg(6) = C25-C26-C27-C28-C28a-C29

8 XYZ-coordinates and FMO energies of compound 1

A single point calculation on the geometry of structure **1** based upon the X-ray structure of S(O)-**1** was performed on the DFT level of theory (Gaussian03, B3LYP/6-311G*).[1]

XYZ-coordinates:

O	-0.471	-1.8793	4.0335
O	-1.014	2.1147	0.4955
O	5.61	-1.0523	-0.3045
N	-3.003	-1.7603	-0.8525
O	4.433	0.7007	-3.0325
N	3.297	-0.7883	-1.8175
N	5.4	1.0667	0.4575
H	4.838	1.7107	0.5485
C	-3.995	0.3787	4.0795
H	-4.672	0.2107	4.6925
C	-2.845	-0.3993	4.1005
H	-2.759	-1.0873	4.7205
C	-1.827	-0.1493	3.1945
C	-0.6	-0.9593	3.2465
C	0.489	-0.6343	2.2975
C	1.699	-1.3323	2.3435
H	1.833	-1.9723	3.0045
C	2.681	-1.0853	1.4345
H	3.483	-1.5543	1.4845
C	2.502	-0.1393	0.4325
C	3.511	0.0957	-0.6605
H	3.393	1.0177	-0.9735
C	2.676	-2.1063	-1.6335
H	3.059	-2.5253	-0.8465
H	2.89	-2.6643	-2.3975
C	1.178	-2.0573	-1.4745
C	0.532	-2.9223	-0.6255
H	1.025	-3.5593	-0.1605
C	-0.849	-2.8603	-0.4455
C	-1.632	-1.8973	-1.1075
C	-3.787	-0.8693	-1.7245
H	-4.712	-1.1553	-1.7095
H	-3.465	-0.9663	-2.6345
C	-3.725	0.6007	-1.3455
H	-4.175	0.7307	-0.4945
H	-2.799	0.8657	-1.2355
C	-4.368	1.4707	-2.3775

[1] Frisch, M. J.; Trucks, G. W.; Schlegel, H. B.; Scuseria, G. E.; Robb, M. A.; Cheeseman, J. R.; Scalmani, G.; Barone, V.; Mennucci, B.; Petersson, G. A.; Nakatsuji, H.; Caricato, M.; Li, X.;. Hratchian, H. P; Izmaylov, A. F.; Bloino, J.; Zheng, G.; Sonnenberg, J. L.; Hada, M.; Ehara, M.; Toyota, K.; Fukuda, R.; Hasegawa, J.; Ishida, M.; Nakajima, T.; Honda, Y.; Kitao, O.; Nakai, H.; Vreven, T.; Montgomery Jr., J. A.; Peralta, J. E.; Ogliaro, F.; Bearpark, M.; Heyd, J. J.; Brothers, E.; Kudin, K. N.; Staroverov, V. N.; Kobayashi, R.; Normand, J.; Raghavachari, K.; Rendell, A.; Burant, J. C.; Iyengar, S. S.; Tomasi, J.; Cossi, M.; Rega, N.; Millam, J. M.; Klene, M.; Knox, J. E.; Cross, J. B.; Bakken, V.; Adamo, C.; Jaramillo, J.; Gomperts, R.; Stratmann, R. E.; Yazyev, O.; Austin, A. J.; Cammi, R.; Pomelli, C.; Ochterski, J. W.; Martin, R. L.; Morokuma, K.; Zakrzewski, V. G.; Voth, G. A.; Salvador, P.; Dannenberg, J. J.; Dapprich, S.; Daniels, A. D.; Farkas, O.; Foresman, J. B.; Ortiz, J. V.; Cioslowski, J.; Fox, D. J. GAUSSIAN 09 (Revision A.02) Gaussian, Inc., Wallingford CT, **2009**.

H	-5.299	1.2127	-2.4765
H	-3.927	1.3317	-3.2305
C	-4.292	2.9497	-2.0045
H	-3.369	3.2157	-1.8755
H	-4.777	3.1127	-1.1805
C	-2.947	-3.2263	1.0905
C	-3.489	-3.6673	2.2825
H	-3.043	-4.3163	2.7795
C	-4.673	-3.1623	2.7435
H	-5.026	-3.4403	3.5585
C	-5.332	-2.2263	1.9695
H	-6.15	-1.8913	2.2585
C	-4.807	-1.7803	0.7845
H	-5.278	-1.1543	0.2835
C	-3.578	-2.2493	0.3155
C	-4.143	1.3787	3.1775
H	-4.927	1.8787	3.1715
C	-4.955	3.7977	-3.2225
H	-4.448	3.7517	-4.0475
H	-5.889	3.5867	-3.3795
C	-3.141	1.6667	2.2655
H	-3.242	2.3627	1.6565
C	-1.973	0.8957	2.2735
C	-0.897	1.1997	1.2915
C	0.326	0.3727	1.3375
C	1.332	0.5977	0.4175
H	1.221	1.2597	-0.2265
C	4.963	-0.0313	-0.1565
C	6.76	1.2877	0.9965
C	7.773	1.1587	-0.1045
H	7.651	1.8727	-0.7365
H	7.657	0.3147	-0.5495
H	8.657	1.2077	0.2655
C	-4.793	4.8147	-2.6225
H	-4.264	4.6427	-1.8405
H	-5.646	5.1707	-2.3605
H	-4.339	5.4487	-3.1835
C	6.759	2.6997	1.5915
H	5.972	2.8187	2.1285
H	6.763	3.3447	0.8805
H	7.54	2.8167	2.1365
C	7.022	0.2777	2.0985
H	7.028	-0.6063	1.7265
H	6.333	0.3427	2.7635
H	7.874	0.4607	2.5035
C	3.814	-0.3513	-2.9885
C	3.613	-1.1753	-4.2415
H	4.192	-1.9403	-4.2165
H	3.819	-0.6413	-5.0115
H	2.699	-1.4663	-4.2885
C	0.39	-1.1493	-2.1825
H	0.801	-0.5803	-2.7945
C	-0.974	-1.0653	-2.0105
H	-1.461	-0.4463	-2.5035

SCF Done: E(RB+HF-LYP) = -2451.26104085   A.U. after   11 cycles

LUMO	-2.982 eV
HOMO	-4.865 eV
